# Triggered Polymersome Fusion

**DOI:** 10.1021/jacs.2c13049

**Published:** 2023-03-06

**Authors:** Stephen D. P. Fielden, Matthew J. Derry, Alisha J. Miller, Paul D. Topham, Rachel K. O’Reilly

**Affiliations:** † School of Chemistry, University of Birmingham, Edgbaston, Birmingham B15 2TT, UK; ‡ Aston Advanced Materials Research Centre, Aston University, Birmingham B4 7ET, UK

## Abstract

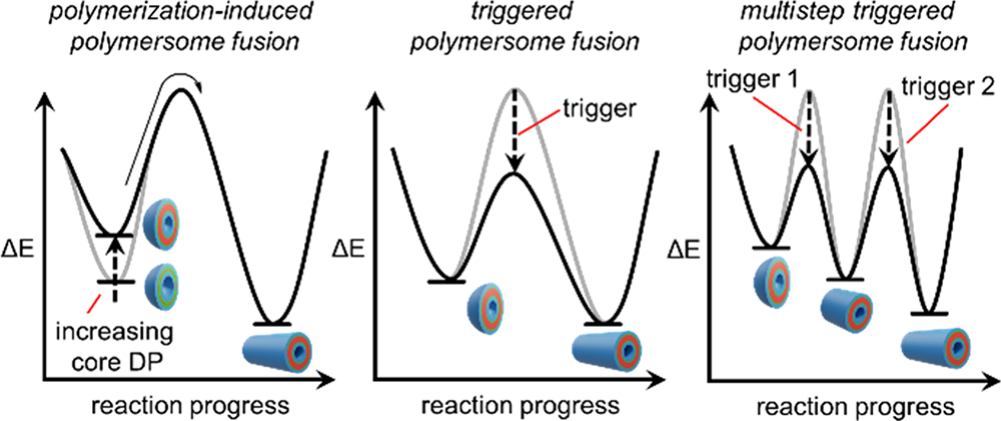

The contents of biological cells are retained within compartments formed of phospholipid membranes. The movement of material within and between cells is often mediated by the fusion of phospholipid membranes, which allows mixing of contents or excretion of material into the surrounding environment. Biological membrane fusion is a highly regulated process that is catalyzed by proteins and often triggered by cellular signaling. In contrast, the controlled fusion of polymer-based membranes is largely unexplored, despite the potential application of this process in nanomedicine, smart materials, and reagent trafficking. Here, we demonstrate triggered polymersome fusion. Out-of-equilibrium polymersomes were formed by ring-opening metathesis polymerization-induced self-assembly and persist until a specific chemical signal (pH change) triggers their fusion. Characterization of polymersomes was performed by a variety of techniques, including dynamic light scattering, dry-state/cryogenic-transmission electron microscopy, and small-angle X-ray scattering (SAXS). The fusion process was followed by time-resolved SAXS analysis. Developing elementary methods of communication between polymersomes, such as fusion, will prove essential for emulating life-like behaviors in synthetic nanotechnology.

## Introduction

**Figure 1:**
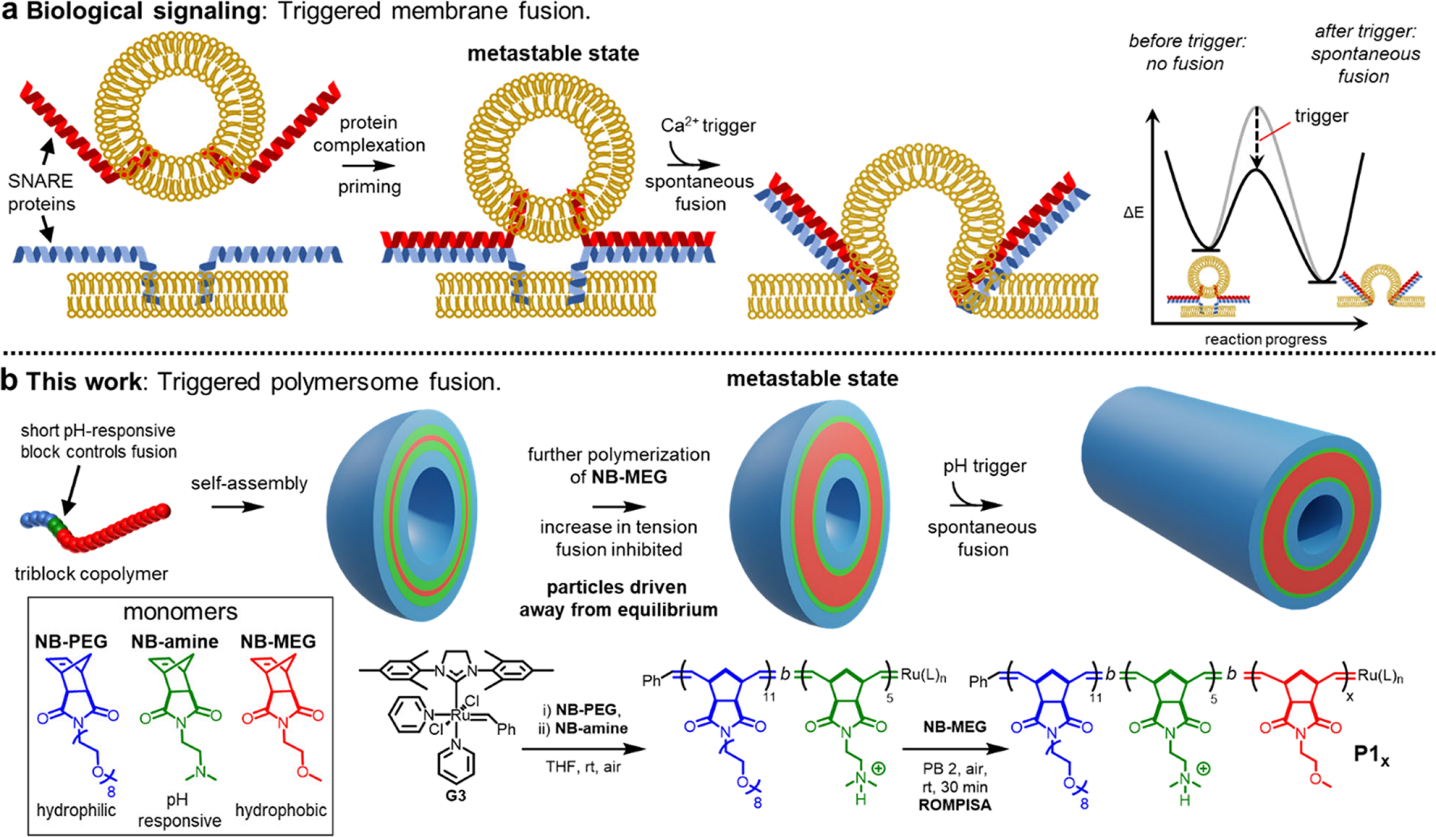
(a) Triggered biological membrane fusion proceeds over two steps. First, membranes are brought into close contact by the complementary interactions of SNARE proteins to generate a metastable state (with respect to the final fused product). Next, an influx of Ca^2+^ promotes protein rearrangement, which triggers fusion of the two membranes. (b) In this work, polymersomes are produced in a persistent metastable state. They then undergo rapid fusion on application of a trigger. Relative thicknesses of layers in polymersome cartoons are not to scale.

Phospholipid membrane-delineated compartments are present in biological cells to control the diffusion of material and allow incompatible processes to occur simultaneously.^1,2^ The fusion of phospholipid membranes results in the merging of compartments and is therefore a fundamental mechanism for controlling the movement of material within and between cells.^3−7^ Many regulated biological signaling processes, such as immune responses,^8^ hormone release,^9^ and nerve propagation,^10^ are coordinated by phospholipid membrane fusion. This is possible because the fusion of phospholipid membranes is not normally spontaneous; it carries a large energetic barrier due to the need to increase phospholipid membrane curvature/tension and overcome charge repulsion from approaching lipid headgroups.^11^ This means that fusion only occurs when additional biological machinery is employed, permitting regulation of the process. Specifically, phospholipid membrane fusion is catalyzed over a two-step process by a family of membrane-bound proteins, situated on the two phospholipid membranes undergoing fusion.^12−16^ These proteins, termed SNAREs (soluble *N*-ethylmaleimide-sensitive factor attachment protein receptors), first form complementary interactions with each other that tether two phospholipid membranes in a metastable state. While this also provides the driving force required to overcome the energetic barrier (Figure 1a), a further stimulus is then often required to trigger the collapse of this intermediate and thus promote phospholipid membrane fusion. Directing membrane fusion through a two-step process permits rapid and coordinated fusion in response to triggering.^17,18^

The need for a trigger also allows membrane fusion to occur in response to environmental changes, thus allowing it to be coupled to other cellular processes. Such regulated fusion therefore provides an important mechanism for mediating cellular communication. For example, when a nerve signal reaches a synapse, an influx of Ca^2+^ triggers the exocytosis (i.e., fusion with the outer cell membrane) of synaptic vesicles, resulting in the secretion of neurotransmitters and signal propagation.^19−21^ The Ca^2+^ trigger functions by lowering the activation energy to fusion. Scientists have applied this biological machinery to direct the fusion of liposomes, artificial vesicles formed of phospholipids.^22^ It has also been possible to use synthetic chemistry to direct liposome fusion via molecular recognition as a driving force.^23−26^

While phospholipid membrane fusion is a key process found in biological cells, the fusion of polymersomes remains an underexplored process.^27−31^ Of the limited number of examples of polymersome fusion,^32−45^ none occur via a triggered two-step mechanism as seen in biological signaling. The generation of a metastable intermediate that remains kinetically inert until a trigger is present is essential for developing responsive and/or stepwise fusion sequences. This biomimetic approach to polymer membrane fusion would find a wide array of applications, such as sequence control over reagent trafficking and catalysis. These could not be effectively coordinated using previously reported methods of spontaneous polymersome fusion.

Here, we demonstrate a polymersome system that undergoes fusion due to the action of a trigger (Figure 1b). This method exploits the ability to form polymer nanoparticles with high membrane curvature in an out-of-equilibrium state. This is achieved using ring-opening metathesis polymerization-induced self-assembly (ROMPISA) of substituted norbornene monomers.^39,46−54^ ROMP has previously been used^55,56^ to access a variety of nanoscopic assemblies, such as bottlebrushes,^57^ dendrimers,^58^ porous materials,^59^ and complex phase separated networks.^60^ In aqueous ROMPISA, rapid polymerization and self-assembly occur simultaneously.^61,62^ As a hydrophilic macroinitiator, derived of P(**NB-PEG**) and **G3**, is chain extended using hydrophobic **NB-MEG**, the resulting amphiphilic diblock copolymer self-assembles in aqueous solution.

Initially, self-assembly results in the formation of small spherical polymersomes. The hydrophobic portions of the constituent polymer chains adopt a rod-like conformation to give a glassy membrane.^63,64^ Continued polymerization of **NB-MEG** after polymersome self-assembly initially serves to drive the system away from thermodynamic equilibrium. This is because the polymersomes are unable to undergo further morphological transformation to accommodate the growing polymer chains, meaning that the free energy released from polymerization becomes stored as membrane tension rather than being dissipated.^65−67^ The release of this tension provides a driving force for fusion, circumventing the need for additional machinery to impart local membrane deformations.^68^ In contrast, lipid-based membranes, as found in biological systems, behave with fluid-like behavior. This is because the constituent lipid chains have a low molecular weight that precludes chain entanglement, permitting the resultant membranes to rapidly rearrange to minimize curvature or in response to a shear force. This means that lipid membranes cannot store free energy as tension or persist in a out-of-equilibrium anisotropic morphology. This necessitates the presence of SNARE proteins to induce the unfavorable membrane geometries required for fusion. Conversely, polymersomes generally possess a significantly greater bending rigidity and lysis tension than lipid vesicles, meaning that they can be subjected to stretching forces without membrane rupture.^11^

It was previously found that as **NB-MEG** polymerization proceeds further, the membrane tension reaches a critical value that causes the isotropic polymersomes to spontaneously fuse together to produce linear tube-like particles (tubesomes).^69−71^ This results in uncontrolled release of stored free energy over the course of several minutes, until polymerization is complete.^39^ The fusion process displays step-growth kinetics; short tubesomes initially form that increase in length as the **NB-MEG** degree of polymerization (DP) increases, and further fusion occurs. Here, we show that the incorporation of stimuli-responsive monomers into ROMPISA polymers permits temporal control over fusion. The on-demand release of free energy stored in the polymersomes is triggered by a pH change, which alters the chemical structure of the membrane corona and consequently lowers the energetic barrier to fusion. This process is rapid (the onset of anisotropy occurs within 5 s), meaning that the fusion of particles is coordinated rather than stochastic. The development of synthetic analogues to triggered biological membrane fusion permits temporal control over the dynamics between polymer nanoparticles. ROMPISA provides the ideal platform for this as it occurs under mild reaction conditions (room temperature, air atmosphere, and aqueous solution).

## Results and Discussion

Previous work has shown that the fusion of ROMPISA polymersomes is inhibited at pH 2 when the corona is formed of P(**NB-amine**·H)^+^.^39^ Such a corona adopts a coil-like conformation in an aqueous environment and provides a barrier to fusion through a combination of charge repulsion and steric hindrance.^64,72^ We therefore reasoned that forming a corona from both **NB-PEG** and **NB-amine** would produce polymersomes that do not fuse at pH 2 but would fuse at higher pH when **NB-amine** becomes deprotonated. This is because **NB-amine** is not hydrophilic at high pH, causing a reduction in corona bulk and removing charge repulsion. The change in hydrophilicity of a short P(**NB-amine**)_5_ block upon deprotonation can be determined *in silico* using our reported method that normalizes the partition coefficient, Log P_oct_, of an oligomer by the solvent-accessible surface area, SA (Log P_oct_/SA).^50,73^ We previously determined the Log P/SA of protonated P(**NB-amine**·H)^+^_5_ to be approximately −0.003 Å^–2^ (i.e., hydrophilic). The calculated value for deprotonated P(**NB-amine**)_5_ is +0.004 (see the Supporting Information, Section S2 for details). This indicates that such a polymer block would indeed be corona forming at low pH and core forming at high pH. If a change in pH occurred quickly, then a “frozen” glassy membrane core could not rearrange into a more stable state before inelastic collisions between particles promotes the loss of membrane tension via fusion.

**Figure 2:**
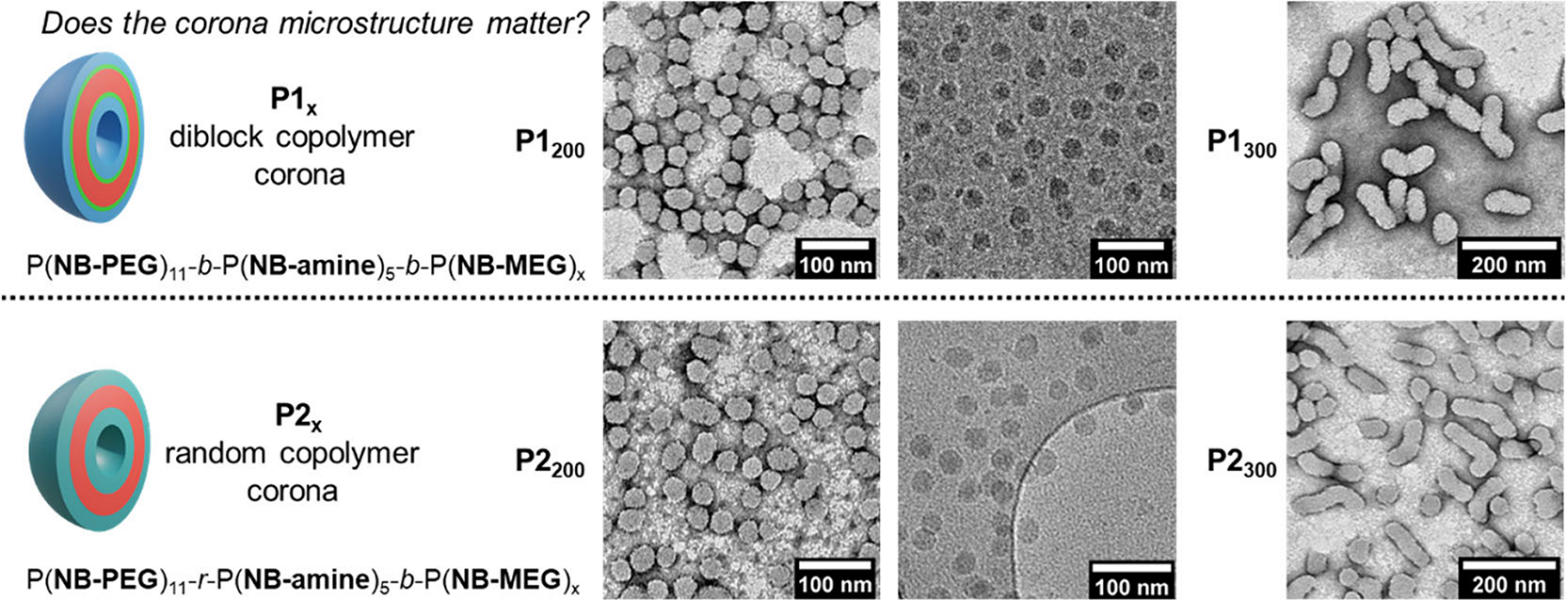
TEM analysis of **P1_*x*_** and **P2_*x*_** nano-object products when **NB-MEG** DP = 200 (unfused) or 300 (fused). **P1_*x*_** particles contain a corona formed of a diblock copolymer (blue and green layers in cartoon). **P2_*x*_** particles contain a corona formed of a random copolymer (aquamarine layer in cartoon). Hydrophobic layers in cartoons are red. Dry-state TEM samples were stained with 1% uranyl acetate solution prior to imaging.

To explore whether triggered polymersome fusion was possible, the pH-responsive amphiphilic block copolymer, **P1_*x*_**, P(**NB-PEG**)_11_-*b*-P(**NB-amine**)_5_-*b*-P(**NB-MEG**)_*x*_, containing a corona formed from discrete blocks of **NB-PEG** with **NB-amine** and a core of **NB-MEG** (DP = *x*) was accessed via ROMPISA (Supporting Information, Section S4). This was performed analogously to a previously reported method of polymersome synthesis.^39^ A second polymer, **P2_*x*_**, P(**NB-PEG**)_11_-*r*-P(**NB-amine**)_5_-*b*-P(**NB-MEG**)_*x*_, containing a corona formed of a random copolymer of **NB-amine** and **NB-MEG** was also investigated (Supporting Information, Section S5) to determine whether the corona microstructure had an effect on the fusion process (Figure 2). This is the first investigation of the effect on the ROMPISA process of altering the microstructure of a corona block containing multiple monomers. To form nano-objects by ROMPISA, the corona was first synthesized by reacting the **G3** initiator with the hydrophilic monomers (sequentially, first, **NB-PEG** and then **NB-amine**, for **P1_*x*_** or simultaneously for **P2_*x*_**) in tetrahydrofuran (THF). The resulting water-soluble macroinitiator was then chain-extended upon addition to a solution of **NB-MEG** dissolved in 100 mM phosphate buffer adjusted to pH 2 (PB2), to give a final solvent composition of THF/PB2 of 1:9 v/v. Consumption of **NB-MEG** was complete within 30 min, as judged by proton nuclear magnetic resonance (^1^H NMR) to give a 1 wt % polymersome dispersion. When a **NB-MEG** DP of 200 (*x* = 200) was targeted, analysis by gel permeation chromatography (GPC; Supporting Information, Figures S2 and S9) indicated controlled polymerization to give both **P1_200_** and **P2_200_** with low dispersity (*Đ* ≤ 1.1). These polymers both self-assembled to give narrowly disperse (polydispersity, PD < 0.10) spherical nano-objects, as determined by dry state/cryo-TEM (Figure 2) and DLS (Supporting Information, Figures S3 and S10). **P1_200_** and **P2_200_** particles had number-average diameters of 36 and 37 nm, respectively, as determined by dry-state TEM (Supporting Information, Figures S4 and S11). DLS measurement gave a *Z*_avg_ of 49 nm for **P1_200_** and 47 nm for **P2_200_**.

Lengthening the core block further increased membrane tension to a level that overcame the threshold for spontaneous fusion to occur.^39^ When a **NB-MEG** DP of 300 was targeted, control over polymerization was retained (*Đ* ≤ 1.1), but the corresponding **P1_300_** and **P2_300_** particles spontaneously fused to give short tubes (Figure 2) with number-average lengths (as determined by dry-state TEM; Supporting Information, Figures S6 and S12) of 97 and 81 nm, respectively (*Z*_avg_ = 98 and 80 nm, respectively, as measured by DLS; Supporting Information, Figures S3 and S10) As both **P1_300_** and **P2_300_** particles underwent spontaneous fusion at a similar core DP, it was concluded that the nature of the corona microstructure did not strongly influence the outcome of uncontrolled fusion at pH 2.

**Figure 3:**
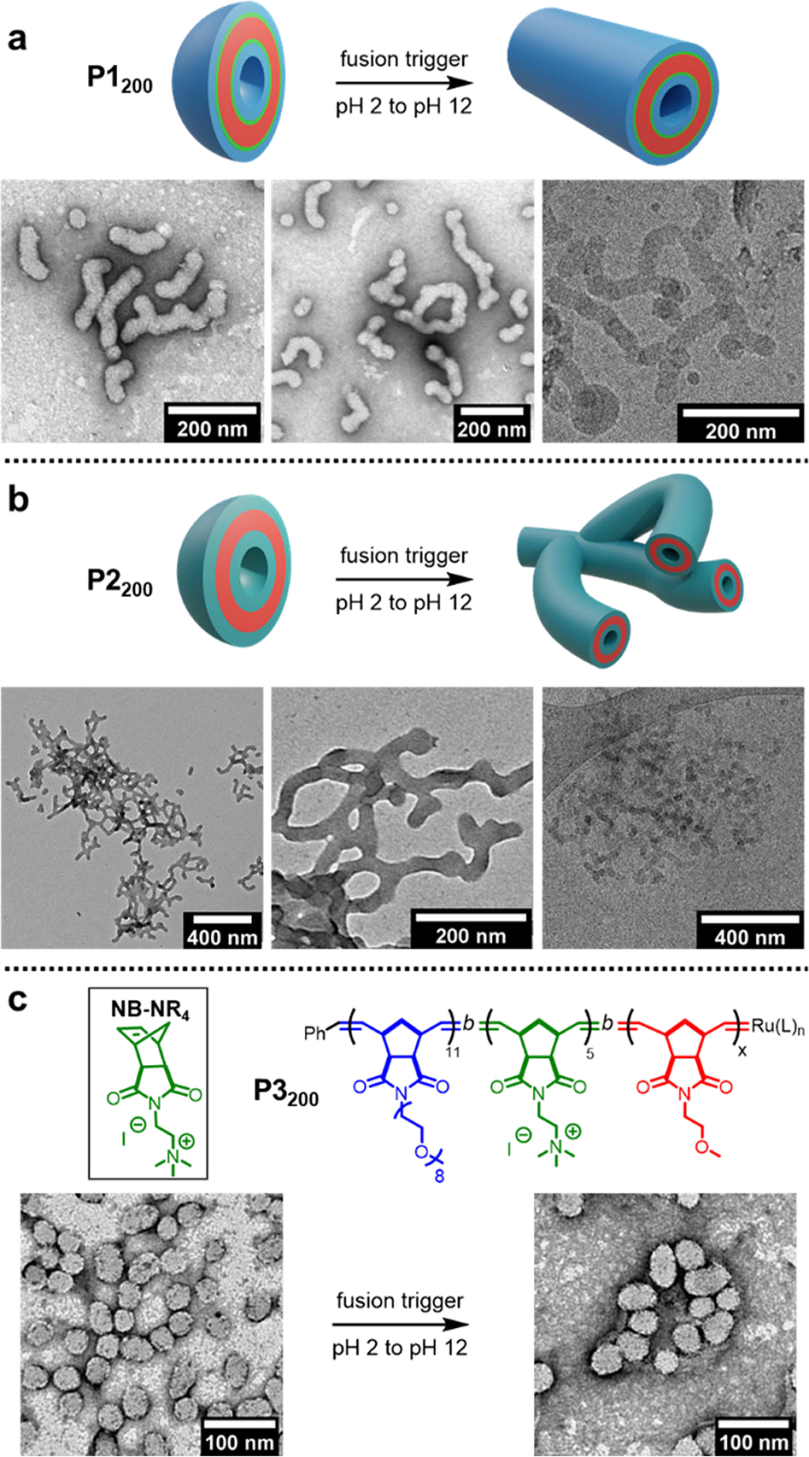
Triggered polymersome fusion by a pH switch. Continuous phase at pH 2: 90 vol % 100 mM PB2 + 10 vol % THF. Continuous phase at pH 12: 90 vol % (25 mM PB2 + 75 mM NaOH) + 10 vol % THF. TEM analysis (dry-state and cryo-) of (a) fused **P1_200_** particles and (b) fused and aggregated **P2_200_** particles. (c) Structure and attempted fusion of **P3_200_**, which contains pH unresponsive P(**NB-NR_4_**) rather than P(**NB-amine**). Dry-state TEM samples were stained with 1% uranyl acetate solution prior to imaging.

Triggered fusion was therefore optimized using particles formed from **P1_200_** or **P2_200_** because these did not undergo spontaneous fusion at pH 2 but should still possess significant membrane tension (Supporting Information, Section S6). In other words, **P1_200_** or **P2_200_** particles formed at pH 2 are metastable structures, similar to an untriggered but tethered SNARE complex. Addition of three volume equivalents of an aqueous NaOH solution (100 mM + 10 vol % THF) to **P1_200_** and **P2_200_** particles (suspended in 100 mM PB2 + 10 vol % THF) switched the pH from 2 to 12 (Figure 3). Triggered fusion was observed only to occur at pH 12 or above due to the high basicity of the amine side chains. For both particles, switching to pH 12 resulted in a rapid increase (<5 s) in turbidity. Analysis of the resultant particles by DLS (Supporting Information, Figure S15) showed both an increase in particle size (*Z*_avg_ = 166 nm for **P1_200_** particles and 303 nm for **P2_200_** particles) and polydispersity (PD = 0.18 for **P1_200_** particles and 0.24 for **P2_200_** particles), indicating an increase in particle size and size distribution, as expected for fusion.^39^ Analysis by dry-state and cryo-TEM showed morphological changes for both samples—discrete, tube-like fused particles (mean length = 99 nm by dry-state TEM; Supporting Information, Figure S16) were formed with **P1_200_** (Figure 3a), and large (>0.5 μm diameter) aggregates of fused particles (Supporting Information, Figure S17) were observed for **P2_200_** (Figure 3b). Maintaining 10 vol % THF upon addition of NaOH was crucial for fusion to occur; if aqueous NaOH containing no THF was added, then no change in morphology of **P1_200_** occurred. THF presumably acts as a plasticizer and facilitates chain rearrangement upon fusion.^74^

We attribute the varying fusion behavior between **P1_200_** and **P2_200_** to the difference in the corona microstructure after deprotonation of P(**NB-amine**·H). For **P1_200_**, the hydrophobic P(**NB-amine**)_5_ block can become buried within the membrane core at pH 12. However, this is not possible for **P2_200_** because the **NB-amine** units are randomly distributed throughout the corona block. Therefore, upon deprotonation, the corona of **P2_200_** contains interspersed hydrophobic patches, which presumably promote higher order aggregation during the fusion process. Similar “patchy” behavior has been previously observed to control aggregation of both synthetic nanoparticles^75,76^ and proteins.^77^ Analysis of dry **P1_200_** and **P2_200_** by differential scanning calorimetry (Supporting Information, Figure S20) allowed *T*_g_ values to be determined for each polymer. The *T*_g_ of **P1_200_** (83 °C) is 8 °C higher than for **P2_200_** (75 °C), suggesting that the latter has greater chain mobility. This greater mobility may also allow fused **P2_200_** particles to form extended aggregates upon pH triggering. Further studies therefore focused on **P1_200_** particles as these fused to give discrete structures, simplifying the analysis of the fusion process.

To demonstrate that triggered fusion is an irreversible process, fused **P1_200_** particles at pH 12 were reacidified back to pH 2. No change in morphology, including fission to reform isotropic particles, was observed by TEM (Supporting Information, Figure S22). This indicates that fusion is irreversible, and fused particles are more thermodynamically stable than unfused particles at both pH values. The pH trigger controls the kinetics of fusion (i.e., the energy maximum), rather than altering the favored morphology (i.e., the energy minimum). If these transformations were instead occurring under thermodynamic control, then reversible morphological changes would be expected on pH toggling.^41^

Despite residing in a metastable state, no change in the morphology of unfused **P1**_200_ particles was observed by TEM for at least 3 months at pH 2 (Supporting Information, Figure S5). We propose that the glassy dynamics of the membrane core hinder chain rearrangement; instead, the tension within the membrane can be harnessed as an energy source to drive fusion when a trigger is applied. No significant change is observed by GPC analysis of **P1_200_** before and after fusion (Supporting Information, Figures S2 and S14), further evidencing that the driving force for fusion is the release of tension, rather than a change in the polymer backbone structure.

It was also important to prove that the pH trigger acted as a specific signal for particle fusion by deprotonating the P(**NB-amine**·H)^+^ units. It was also possible that a pH switch may instead trigger fusion due to an extrinsic change in reaction conditions (e.g., due to a change in salt composition, on dilution, or upon agitation). To rule this out, a polymer analogous to **P1_200_** that was formed using quaternary ammonium monomer **NB-NR_4_**, rather than **NB-amine**, was synthesized by ROMPISA at pH 2 (Supporting Information, Section S7). This polymer, **P3_200_**, P(**NB-PEG**)_11_-*b*-P(**NB-NR_4_**)_5_-*b*-P(**NB-MEG**)_200_, self-assembled to give particles similar to those formed from **P1_200_**, as judged by DLS (*Z*_avg_ = 57 nm; Supporting Information, Figure S24) and dry-state TEM (number average diameter = 42 nm; Supporting Information, Figure S25). Upon switching the pH from 2 to 12, only a small change in mean particle length (to 46 nm; Supporting Information, Figure S27) was observed by dry-state TEM, with a narrow distribution (from 42 ± 9 nm at pH 2 to 46 ± 10 nm at pH 12) of particle lengths being retained. This confirms that a specific pH-responsive functional group is indeed required to facilitate triggering of fusion. This is reminiscent of fusion catalyzed by SNARE proteins, where programmed interactions between SNARE pairs direct the fusion of two specified membranes.^16^ This therefore opens the possibility of using a variety of orthogonal stimuli (e.g., redox switching and host–guest recognition) to choose which polymersomes fuse together within a mixture.

**Figure 4:**
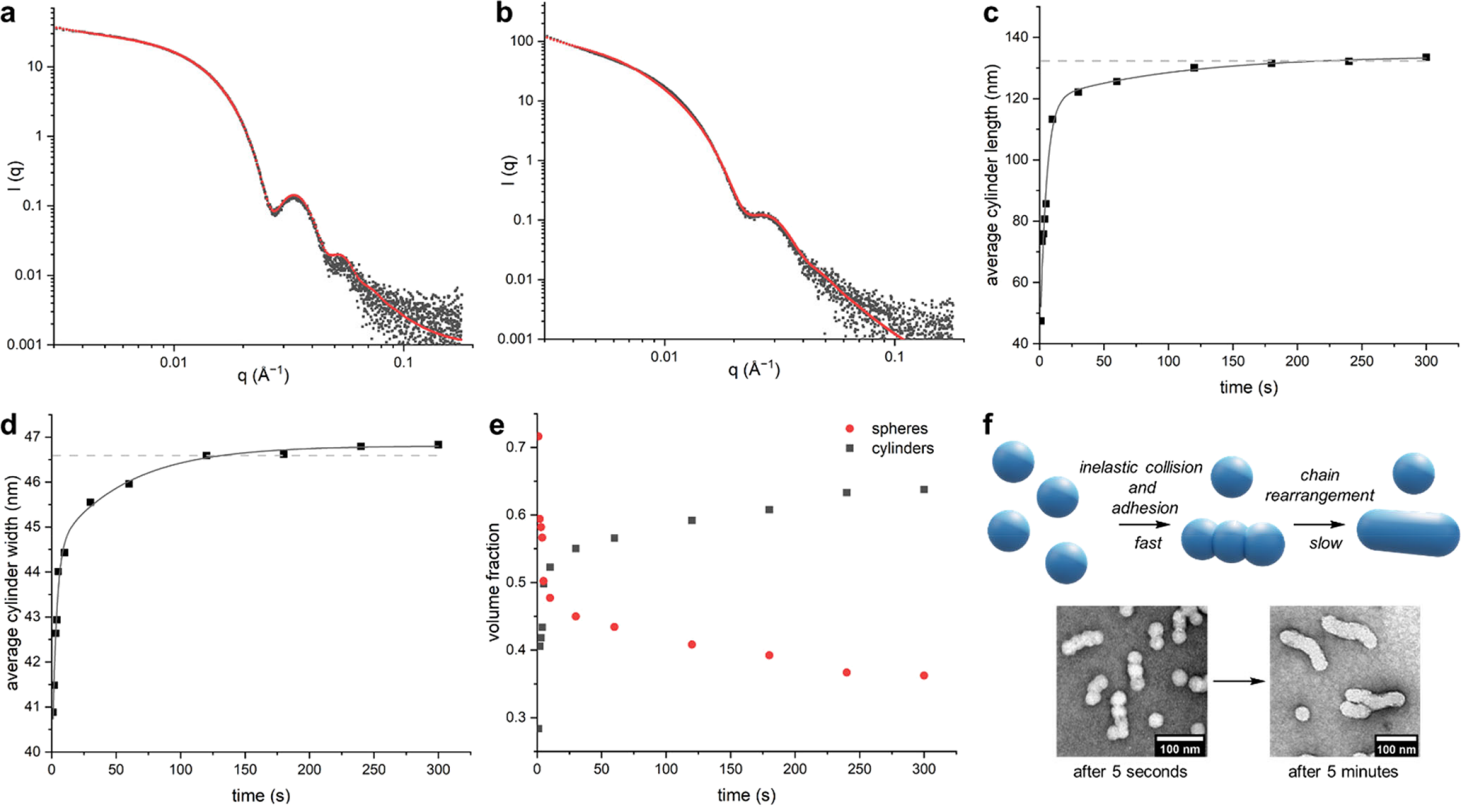
Mechanistic analysis of fusion of **P1_200_** particles by SAXS. (a) Static SAXS data of unfused **P1_200_** particles fitted to a spherical micelle model (red line). (b) Static SAXS data of fused **P1_200_** particles fitted to a cylindrical micelle model (red line). (c) Evolution of cylinder length over time during *in situ* SAXS analysis. The dashed gray line corresponds to the cylinder length from modeling of the static measurement in part (b). Line added to guide the eye. (d) Evolution of cylinder width over time during *in situ* SAXS analysis. The dashed gray line corresponds to the cylinder width from modeling of the static measurement in part (b). Line added to guide the eye. (e) Evolution in volume fraction of spheres [unfused particles] and cylinders [fused particles] during *in situ* SAXS analysis. Values obtained from weighting of models to give the best fit. (f) Proposed two-step fusion mechanism with dry-state TEM images of particles quenched after 5 s and 5 min at pH 12. Dry-state TEM samples were stained with 1% uranyl acetate solution prior to imaging.

Further complementary analysis of fused and unfused **P1_200_** particles was obtained by small-angle X-ray scattering (SAXS; Supporting Information, Section S10). As for previous SAXS analysis of polymersomes obtained by ROMPISA, data obtained for unfused particles at pH 2 were best fitted using a spherical micelle model^78^ (i.e., with a solid polymer core) rather than a vesicle model^79^ (Figure 4a). This is due to the thick membrane being of the order of the overall particle radius, and thus, the model is insensitive to such a small lumen (a few nanometers in diameter).^50^ The overall volume-weighted mean diameter of these particles was determined by SAXS to be 46 nm, which lies between the number-weighted diameter measured by TEM (36 nm) and intensity-weighted diameter determined by DLS analysis (49 nm). The mean aggregation number, *N*_agg_, was found to be 306.^80^

The SAXS data of fused **P1**_200_ particles at pH 12 fitted best to a cylindrical micelle model (Figure 4b). The mean length of particles was 132 nm, and the mean width was 46.6 nm. The mean aggregation number, *N*_agg_, was found to be 1900, implying that, on average, a tube is formed of six fused particles.

Due to the fast onset of fusion (solution turbidity increased within seconds of application of the pH trigger), the process could not be followed by conventional light scattering techniques, such as DLS analysis. Fusion of **P1_200_** was instead probed by time-resolved SAXS analysis using a stopped-flow apparatus to initiate fusion while monitoring using a synchrotron light source.^80−84^ This is the first example of studying particles formed by ROMPISA using time-resolved SAXS. Pre-formed **P1_200_** in PB2 was rapidly mixed with NaOH/THF solution and flowed into the measurement capillary within 10 ms. Scattering data from the resultant solution was acquired at 1 s intervals (500 ms exposure) for 10 min. No significant change in scattering was observed after this time. The first scattering pattern (taken 1 s after mixing) and the pattern taken at 5 min matched well with static SAXS patterns of unfused and fused **P1_200_** particles, respectively (Supporting Information, Figures S37 and S38), where fusion had been initiated by standard pipette mixing. This confirms that the *in situ* SAXS experiment proceeded analogously to conventional laboratory experiments and thus provides an accurate picture of the fusion process.

Time-resolved SAXS data between 1 and 300 s were fitted to a linear combination of the spherical micelle and a cylindrical micelle model previously employed for the static data (see the Supporting Information). Parameters for the unfused spherical particles present at pH 2 were not modified when accounting for their presence on fitting data from the time-resolved experiment: the dimensions of these initial particles remained unchanged throughout the experiment, in line with TEM analysis. Satisfactory fits were obtained for all SAXS patterns using this approach, and it was deduced that the cylinders formed increased in both length (Figure 4c) and width over time (Figure 4d). The volume fraction of cylinders also increased with time (Figure 4e), reflecting the progression of the fusion process. Importantly, the final dimensions of the cylindrical particles (mean length = 133 nm, mean width = 46.8 nm) are in close agreement with those obtained from the static measurement (mean length = 132 nm, mean width = 46.6 nm).

The cylinder length evolved more quickly than cylinder width, which implies a two-step mechanism for fusion: (i) spherical polymersomes rapidly combine to give elongated particles; (ii) these then undergo a slower structural rearrangement to give the final fused product. Presumably, the second step occurs as polymer chains rearrange to minimize the particle surface area.^64^

To further probe the fusion mechanism, two aliquots of **P1_200_** particles undergoing fusion at pH 12 were quenched back to pH 2 after 5 s and 5 min (Supporting Information, Section S8). The aliquots exhibited near-identical DLS data (Supporting Information, Figure S28). Smooth tubes were observed by TEM imaging of particles quenched after 5 min, suggesting that fusion was complete (Figure 4f). However, segmented tubes were observed by TEM imaging of particles quenched after 5 s, suggesting incomplete fusion. This provides further evidence that fusion occurs via rapid adhesion of spherical polymersomes followed by a slower structural rearrangement.

**Figure 5:**
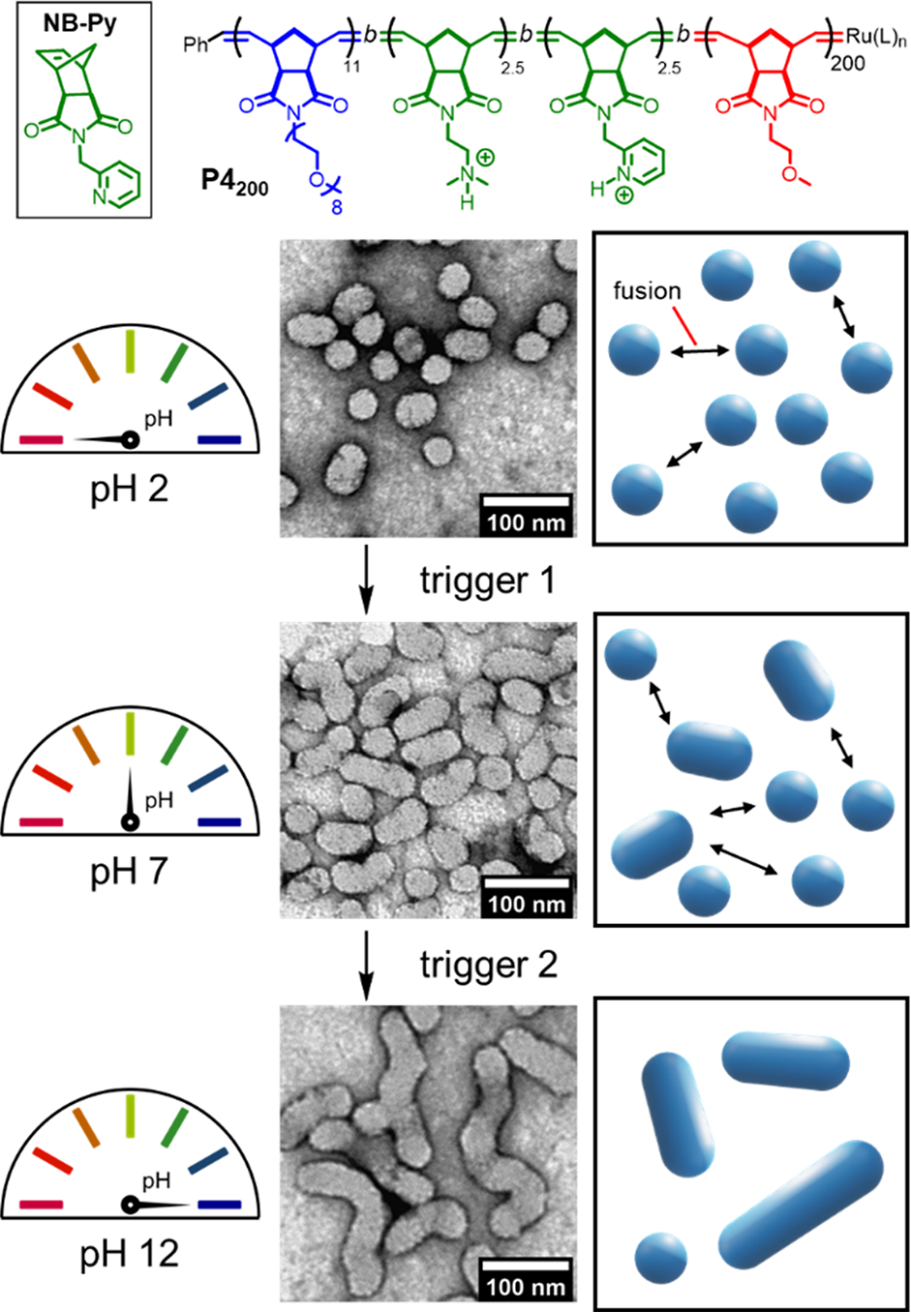
Multistep triggered fusion with tetrablock copolymer **P4_200_** containing two pH-responsive blocks. The first trigger (switching pH from 2 to 7) deprotonates the P(**NB-Py**) block, resulting in partial fusion. The second trigger deprotonates the P(**NB-amine**) block, resulting in complete fusion. Dry-state TEM samples were stained with 1% uranyl acetate solution prior to imaging.

Finally, the fusion of polymers containing two different pH-responsive monomers was studied. Using a combination of monomers with differing p*K*_a_ values should allow fusion to be controlled over a series of triggers, giving greater temporal control of the process. A tetrablock copolymer **P4_200_**, P(**NB-PEG**)_11_-*b*-P(**NB-amine**)_2.5_-*b*-P(**NB-Py**)_2.5_-*b*-P(**NB-MEG**)_200_ (Figure 5 and Supporting Information, Section S9), for which half of the **NB-amine** content of **P1_200_** had been replaced with pyridine containing monomer **NB-Py**, was synthesized via ROMPISA at pH 2. Protonated P(**NB-Py**·H)^+^ should exhibit a p*K*_a_ several units lower than P(**NB-amine**·H)^+^, meaning that it will be deprotonated at a lower pH value.^85^

Particles formed from **P4_200_** had similar dimensions to **P1_200_** particles—with an average length of 43 nm, as judged by dry-state TEM (Supporting Information, Figure S33). A small degree of spontaneous fusion occurred at pH 2, showing that P(**NB-Py**·H)^+^ provided a lower barrier to fusion than P(**NB-amine**·H)^+^. The first trigger for fusion was adjusting the pH from 2 to 7, which resulted in an increased degree of particle fusion, causing the average particle length to increase to 55 nm (Supporting Information, Figure S35). The second trigger further increased the pH to 12, which prompted complete fusion to give particles with an average length of 127 nm (Supporting Information, Figure S36). Thus, the release of membrane tension could be achieved over multiple steps. By incorporating a broader range of responsive monomers, it should be possible to trigger a series of polymersome fusion using combinations of different stimuli.

## Conclusions

The triggered fusion of polymersomes synthesized by ROMPISA has been demonstrated. By identifying suitable polymer structures, it was possible to synthesize unfused polymersomes in an out-of-equilibrium state. The free energy contained as membrane tension in these particles was used to drive particle fusion when triggered by a pH change. The microstructure of the corona was crucial for determining fusion outcome and allowed release of membrane tension to occur over time in a stepwise manner. The process described herein mimics protein-assisted triggering of phospholipid-based membranes found in biological cells, where two membranes are held in an out-of-equilibrium state until a trigger promotes their fusion. This process is essential for intercellular communication and the regulation of many biological phenomena. The trigger mechanism permits temporal control over the fusion process. This in turn allows membrane fusion to regulate and be integrated with metabolic sequences. This work provides an important advance in the development of such temporally controlled processes in non-biological media.

The use of kinetic control to generate out-of-equilibrium states is being increasingly exploited to endow chemical systems with emergent functions, such as directional motion,^86−88^ transient assembly,^89−92^ and concentration gradient formation.^93−96^ However, the application of this approach to nanoscale systems derived from covalent polymers remains largely unexplored.^66,67,97−103^ Here, we demonstrate that assembled glassy polymers can form an out-of-equilibrium assembly that can be used as a transient store of energy. This would not be possible if polymer assembly occurred under thermodynamic control. Controlled dissipation of excess free energy permitted temporal control over polymersome morphology. The wide variety of reported assemblies derived from covalent polymers suggests that there is much to be gained by further investigating how they can be used to produce functional nanotechnology that is formed in the out-of-equilibrium regime. Future work will focus on controlling the selectivity of fusion. Release of free energy could be used to overcome the entropic penalty that arises when fusion is targeted between two polymersome populations (i.e., crossed fusion).^104,105^ The work presented here therefore lays the foundation for developing networks of out-of-equilibrium polymersomes capable of communicating via fundamental processes such as particle fusion.^106,107^ It is clear that developing new mechanisms for temporally controlling the dynamics of polymersomes is essential to advance this area.

## Supporting Information Available

Experimental procedures, synthesis, and characterization data of monomers, polymers, and nanoparticles (NMR, GPC, DLS, TEM, DSC and SAXS) (PDF)

## Abbreviations

*b*: block copolymer
DLS: dynamic light scattering
DP: degree of polymerization
GPC: gel permeation chromatography
NMR: nuclear magnetic resonance spectroscopy
SAXS: small-angle X-ray scattering
PB: phosphate-buffered solution
*r*: random copolymer
ROMPISA: ring-opening metathesis polymerization-induced self-assembly
rt.: room temperature
(cryo-)TEM: (cryogenic-) transmission electron microscopy
THF: tetrahydrofuran
