# Supplementary material for: Triggered Polymersome Fusion
Source: J Am Chem Soc. 2023 Mar 6;145(10):5824–33. doi: 10.1021/jacs.2c13049 (PMC10021019; doi:10.1021/jacs.2c13049)
Supplement: Supplementary file 1 — ja2c13049_si_001.pdf [file ja2c13049_si_001.pdf]

Triggered Polymersome Fusion

Supporting Information

Stephen D. P. Fielden,*[1] Matthew J. Derry,[2] Alisha J. Miller,[1] Paul D. Topham,[2] Rachel K. O'Reilly*[1]

[1]School of Chemistry, University of Birmingham, Edgbaston, Birmingham B15 2TT, UK

[2]Aston Advanced Materials Research Centre, Aston University, Birmingham B4 7ET, UK

*email: s.fielden@bham.ac.uk; r.oreilly@bham.ac.uk

Table of Contents

1. General information and abbreviations S3

1.1 Materials S3

1.2 Characterization Techniques S3

2. Evaluation of P(**NB-amine**)$_5$ hydrophobicity S5

3. Synthesis of monomers S6

3.1 Synthesis of **NB-PEG** via **S1** S6

3.2 Synthesis of **NB-NR$_4$** S10

3.3 Synthesis of **NB-py** S12

4. Synthesis and characterization of P(**NB-PEG**)$_{11}$-*b*-P(**NB-amine**)$_5$-*b*-P(**NB-MEG**)$_{200/300}$, **P1$_{200}$** and **P1$_{300}$** S14

5. Synthesis and characterization of P(**NB-PEG**)$_{11}$-*r*-P(**NB-amine**)$_5$-*b*-P(**NB-MEG**)$_{200/300}$, **P2$_{200}$** and **P2$_{300}$** S20

6. Triggered fusion of **P1$_{200}$** and **P2$_{200}$** S25

7. Synthesis of P(**NB-PEG)**$_{11}$-*b*-P(**NB-NR$_4$**)$_5$-*b*-P(**NB-MEG**)$_{200}$, **P3$_{200}$** S29

8. Interrupted fusion experiments S34

9. Synthesis and characterization of P(**NB-PEG**)$_{11}$-*b*-P(**NB-amine**)$_{2.5}$-*b*-P(**NB-py**)$_{2.5}$-*b*-P(**NB-MEG**)$_{200}$, **P4$_{200}$** S36

10. SAXS analysis of the fusion of **P1$_{200}$** particles S42

10.1 SAXS Modelling S42

10.2 Comparison of static and *in situ* SAXS analysis S48

10.3 Representative example of modelled *in situ* SAXS data S50

10.4 Synthesis of P(**NB-MEG**)$_{200}$ S51

11. References S53

1. General information and abbreviations

1.1 Materials

Unless stated otherwise, reagents were obtained from commercial sources and used without purification. Tetrahydrofuran (THF) (HPLC grade) was purchased from VWR Chemicals and was purified via passage through a column of neutral alumina prior to use. Formvar-carbon coated (300 mesh) and lacey-carbon coated (400 mesh) copper grids were purchased from EM Resolutions.

Flash column chromatography was carried out using silica (particle size 40–63 µm) as the stationary phase. TLC was performed on precoated silica gel plates and visualized using short wave ultraviolet light in combination with standard laboratory stains (basic potassium permanganate and iodine vapour).

G3 (modified Grubbs 3rd generation catalyst),[1] **NB-amine**,[2] **NB-MEG**[2] and exo-5-norbornene imide[2] were prepared as previously described.

1.2 Characterization Techniques

NMR Spectroscopy. ^{1}H NMR and ^{13}C NMR spectra were recorded at 300 MHz or 400 MHz on a Bruker DPX-300 or a Bruker DPX-400 spectrometer in $CDCl_3$, $(CD_3)_2SO$ or D_2O. Chemical shifts of protons are reported as δ in parts per million (ppm) and are relative to solvent residual peaks ($CDCl_3$ δ = 7.26 ppm, $(CD_3)_2SO$ δ = 2.50 ppm, D_2O δ = 4.79 ppm). All ^{1}H resonances are reported to the nearest 0.01 ppm. The multiplicity of ^{1}H signals are indicated as: s = singlet; d = doublet; t = triplet; q = quartet; m = multiplet; or combinations of thereof. Coupling constants (J) are quoted in Hz and reported to the nearest 0.1 Hz. Where appropriate, averages of the signals from peaks displaying multiplicity were used to calculate the value of the coupling constant. ^{13}C NMR spectra were recorded on the same spectrometer with the central resonance of the solvent peak as the internal reference ($CDCl_3$ δ = 77.16 ppm). All ^{13}C resonances are reported to the nearest 0.01 ppm.

High-Resolution Mass Spectrometry. HRMS spectra were recorded by the MS Analytical Facility Service at the University of Birmingham on a Waters Xevo G2-XS QTof Quadrupole Time-of-Flight mass spectrometer.

Gel permeation chromatography. Gel permeation chromatography (GPC) analysis was performed on a system composed of an Agilent 1260 Infinity II LC system equipped with an Agilent guard column (PLGel 5 µM, 50 × 7.5 mm) and two Agilent Mixed-C columns (PLGel 5 µM, 300 × 7.5 mm). The mobile phase used was either DMF (HPLC grade) containing 5 mM NH_4BF_4 at 50 °C with a flow rate of 1.0 mL min^{-1} (poly(methyl methacrylate) (PMMA) standards used for calibration), or THF (HPLC grade) containing 2% v/v NEt_3 at 40 °C with a flow rate of 1.0 mL min^{-1} (poly(methyl methacrylate) (PMMA) standards used for calibration). Number average molecular weight (M_n), weight average molecular weight (M_w) and dispersity ($Đ = M_w/M_n$) were determined using either Wyatt ASTRA v7.1.3 or Agilent GPC/SEC software.

Dynamic Light Scattering. Hydrodynamic diameters (D_h) and size distributions (PD) of nano-objects were determined by dynamic light scattering (DLS) using a Malvern Zetasizer Nano ZS with a 4 mW He-Ne 633 nm laser module operating at 25 °C. Samples were diluted with PB2 to give a final polymer concentration of 0.01 wt%. Measurements were carried out at an angle of 173° (back scattering), and results were analyzed using Malvern DTS v7.03 software. All determinations were repeated four times

with at least 10 measurements recorded for each run. D_h values were calculated using the Stokes-Einstein equation where particles are assumed to be spherical, while for anisotropic particles DLS was used to indicate a change in average particle size and obtain dispersity information.

Transmission Electron Microscopy. Dry-state stained transmission electron microscopy (TEM) imaging was performed on a JEOL JEM-1400 microscope operating at an acceleration voltage of 80 kV. All dry-state samples were diluted with PB2 to an appropriate analysis concentration and then deposited onto formvar-coated grids. After roughly 1 min, excess sample was blotted from the grid and the grid was then stained with an aqueous 1 wt% uranyl acetate (UA) solution for 1 min prior to blotting, drying and microscopic analysis. Cryogenic transmission electron microscopy (cryo-TEM) imaging was performed on a JEOL JEM-2100Plus microscope operating at an acceleration voltage of 200 kV. Samples for cryo-TEM analysis were prepared, after dilution with PB2, by depositing 8 µL of sample onto a lacey carbon grid followed by blotting for approximately five seconds. The grid was then plunged into a pool of liquid ethane, cooled using liquid nitrogen, to vitrify the sample. Transfer into a pre-cooled cryo-TEM holder was performed under liquid nitrogen temperatures prior to microscopic analysis. Images were analyzed using ImageJ.

Differential Scanning Calorimetry. Determination of the glass transition temperatures (T_g) was performed using a Mettler Toledo DSC 3 differential scanning calorimeter by heating the sample from 25 °C to 200 °C at a rate of 10 °C/min for two heating/cooling cycles. The T_g was determined from the inflection point in the second heating cycle of DSC. Collected data were processed using STARe software.

Small-angle X-ray scattering. SAXS patterns were recorded at a synchrotron source (Diamond Light Source, station I22, Didcot, UK; Experiment ID SM28511) using monochromatic X-ray radiation (X-ray wavelength λ = 1.00 Å, with scattering vector q ranging from 0.0017 to 0.17 $Å^{-1}$, where $q = 4\pi \sin\theta/\lambda$ and θ is one-half of the scattering angle) and a 2D Pilatus 2M pixel detector (Dectris, Switzerland). All static SAXS measurements were performed on 1.0% w/w copolymer dispersions in 2.0 mm glass capillaries. Time-resolved experiments were performed using a BioLogic SFM-400 stopped-flow mixing system equipped with an umbilical connector, mixing unit and observation cell containing a 1.0 mm glass capillary, without delay lines: a 1.0% w/w polymersome dispersion in pH2 water and aqueous NaOH were loaded into two separate 10 mL syringes before 200 µL of each solution was flowed into the observation cell at 1.0 mL s^{-1} so that a final solution pH of 12 and a copolymer concentration of 0.5% w/w was obtained on mixing. Scattering data were reduced and normalized, with glassy carbon being used for the absolute intensity calibration, utilizing standard routines available at the beamline[3] and further analyzed (background subtraction and data modelling) using Irena SAS macros for Igor Pro.[4]

2. Evaluation of P(NB-amine)$_5$ hydrophobicity

LogP_{oct} Analysis. Octanol-water partition coefficients (LogP_{oct}) for P(**NB-amine**)$_5$ were calculated in Materials Studio 2020,[5] using an atom-based approach (ALogP98 method)[6] for a molecular model containing C, H, N, and O atoms.

Surface Area Analysis. The octanol-water partition coefficient (LogP_{oct}) was normalized by solvent accessible surface area (SA) using Materials Studio 2020. First, the oligomer was subjected to a Geometry Optimization procedure using the Forcite Molecular Dynamics (MD) module with a COMPASS II force field. The force field contains information on important parameters, like preferred bond lengths, bond angles, torsion angles, partial charges, and van der Waals radii that influence the conformation.[7] To minimize energy and determine a preferred conformation, this simulation ran until the energy of the oligomer decreased below predetermined convergence criteria (1×10^{-4} kcal mol^{-1} energy convergence, 0.005 kcal mol^{-1}/Å force convergence, and 5×10^{-5} Å displacement convergence). Second, the SA value represents the Connolly surface area created by an algorithm that rolls a ball over the surface of the oligomer. To ensure the SA values are meaningful in the context of octanol-water partition coefficients (LogP_{oct}), the probe had a 1.4 Å radius to match the size of a water molecule. Third, the oligomer was annealed for 200 cycles using a sinusoidal temperature ramp (300 – 700 K) to maximize variability in SA values.

Model. P(**NB-amine**)$_5$ containing 1:1 cis/trans alkene bonds.

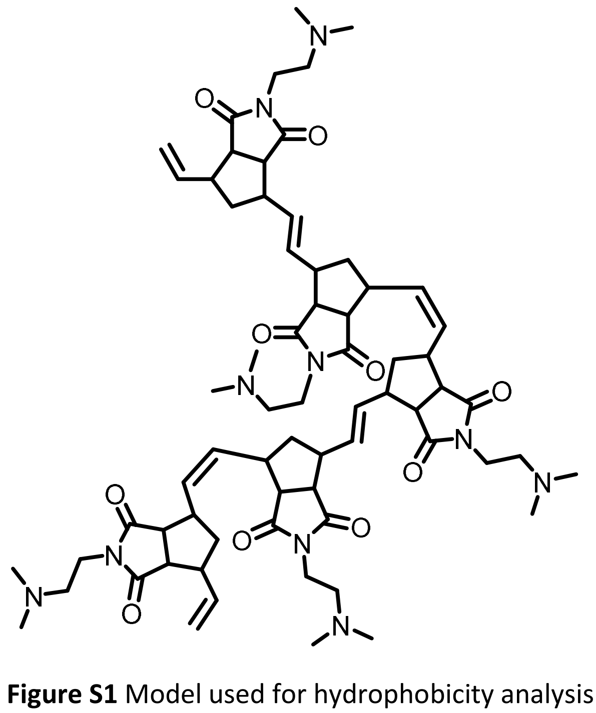

Figure S1 Model used for hydrophobicity analysis.

3. Synthesis of monomers

3.1 Synthesis of NB-PEG via S1

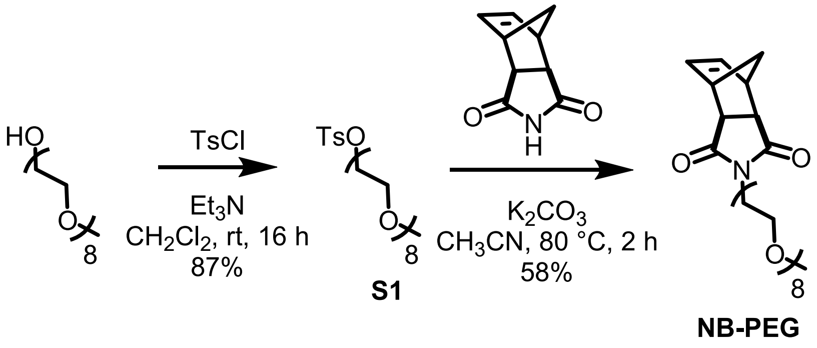

Scheme S1 Synthesis of **NB-PEG** from **S1**.

Synthesis of S1

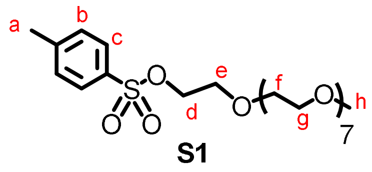

To a solution of methoxypolyethylene glycol (M_n = 350) (1.0 g, 2.9 mmol, 1.0 eq.) and p-toluenesulfonyl chloride (709 mg, 3.7 mmol, 1.3 eq.) in CH_2Cl_2 (25 mL) was added triethylamine (596 mL, 4.3 mmol, 1.5 eq.). The resulting solution was stirred for 16 h. The solvent was then removed under reduced pressure. The residue was purified by flash column chromatography (SiO_2, CH_2Cl_2 to 95:5 CH_2Cl_2/MeOH) to yield **S1** (1.28 g, 2.5 mmol, 87 %) as a colorless oil.

^{1}H NMR (400 MHz, $CDCl_3$, 298 K): δ 7.88 – 7.62 (m, 2H, H_c), 7.29 (d, J = 8.1 Hz, 2H, H_b), 4.14 – 4.06 (m, 2H, H_d), 3.66 – 3.46 (m, 26H, $H_{e,f,g}$), 3.31 (d, J = 1.2 Hz, 3H,H_h), 2.39 (s, 3H, H_a).

^{13}C NMR (101 MHz, $CDCl_3$, 298 K) δ 144.89, 133.13, 129.93, 128.09, 72.04, 70.86, 70.80 – 70.54 (m), 69.35, 68.79, 59.14, 21.75.

HRMS (ESI$^+$) Calculated for $C_{24}H_{42}O_{11}SNa$ $[M+Na]^+$ 561.2346, found 561.2347.

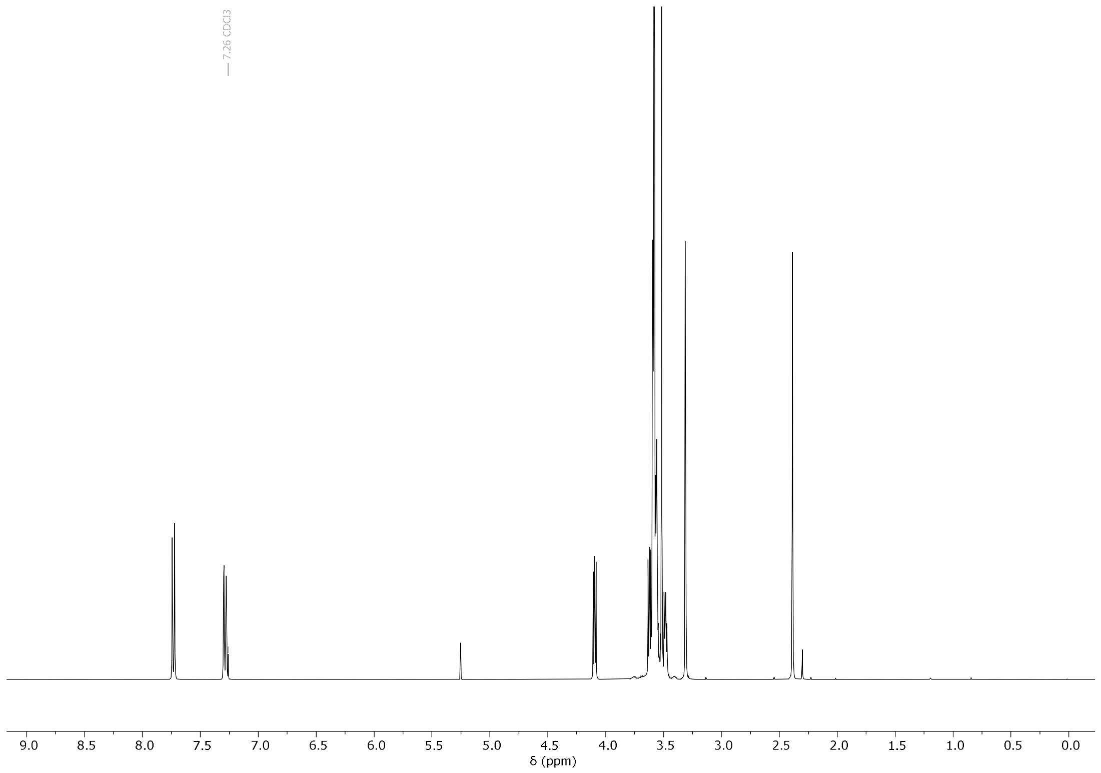

Spectrum S1 ^{1}H NMR (400 MHz, $CDCl_3$, 298 K) of **S1**

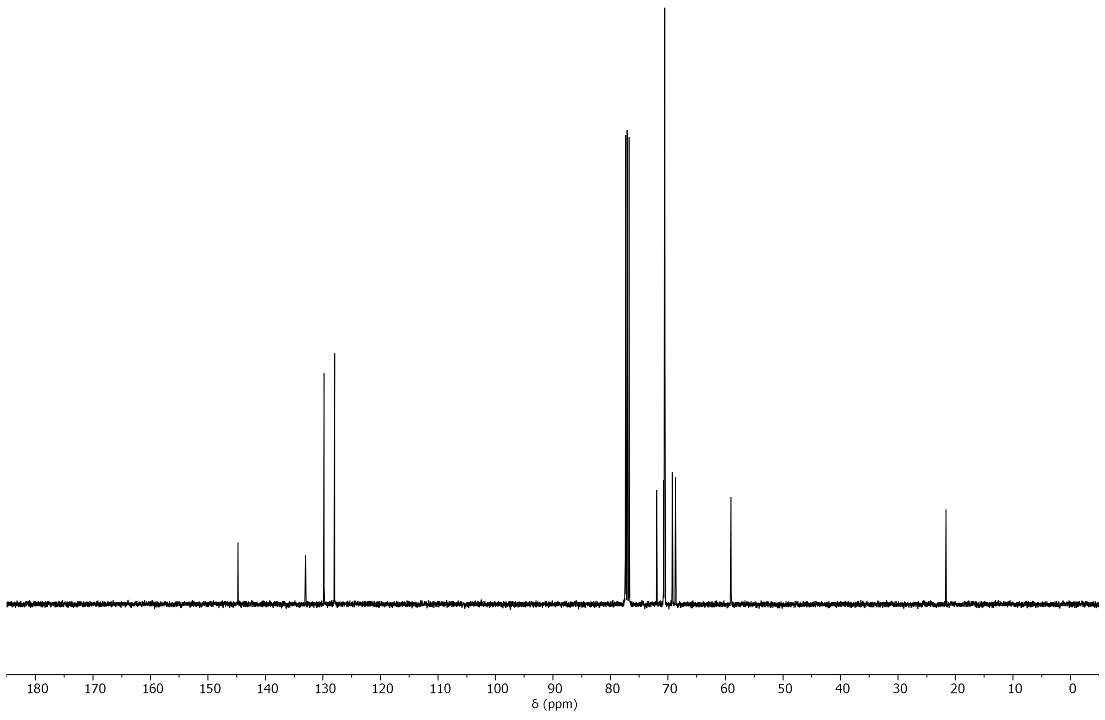

Spectrum S2 ^{13}C NMR (101 MHz, $CDCl_3$, 298 K) of **S1**

Synthesis of NB-PEG

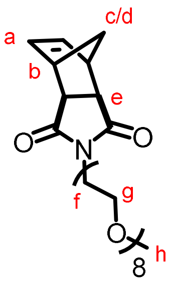

NB-PEG

To a solution of **S1** (840 g, 1.7 mmol, 1.0 eq.) and exo-5-norbornene imide (328 mg, 2.0 mmol, 1.2 eq.) in CH_3CN (3 mL) was added K_2CO_3 (275 mg, 2.0 mmol, 1.2 eq.). The resulting suspension was heated to 80 °C for two hours and then cooled. The solvent was then removed under reduced pressure. The residue was purified by flash column chromatography (SiO_2, CH_2Cl_2 to 95:5 CH_2Cl_2/MeOH) to yield **NB-PEG** (540 mg, 1.0 mmol, 58 %) as a colorless oil.

^{1}H NMR (400 MHz, $CDCl_3$, 400 K) δ 6.26 (t, J = 1.9 Hz, 2H, H_a), 3.77 – 3.48 (m, 27H, H_{f+g}), 3.36 (s, 3H, H_h), 3.30 – 3.19 (m, 2H, H_b), 2.66 (d, J = 1.4 Hz, 2H, H_e), 1.46 (dt, J = 9.9, 1.6 Hz, 1H, $H_{c/d}$), 1.35 (dt, J = 9.8, 1.6 Hz, 1H, $H_{c/d}$).

^{13}C NMR (101 MHz, $CDCl_3$, 298 K) δ 178.12, 137.96, 72.07, 71.14 – 70.33 (m), 70.00, 67.02, 59.17, 47.95, 45.41, 42.85, 37.86.

HRMS (ESI$^+$) Calculated for $C_{26}H_{43}NO_{10}Na$ $[M+Na]^+$ 552.2784, found 552.2785.

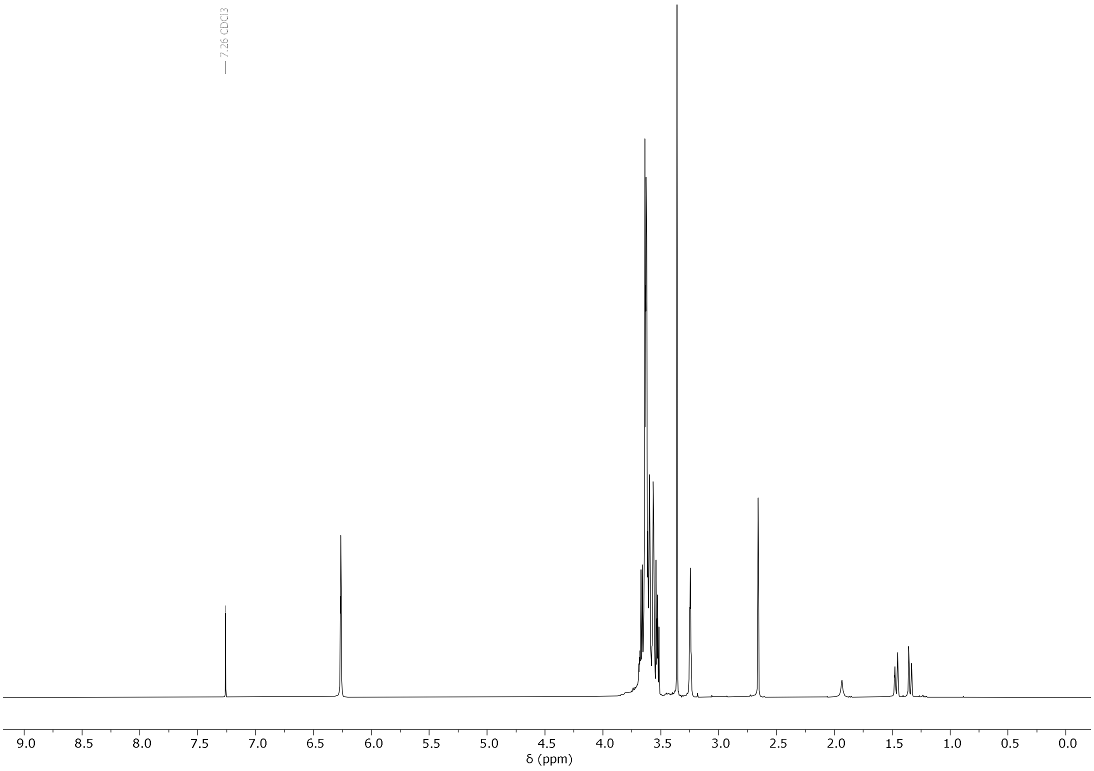

Spectrum S3 ^{1}H NMR (400 MHz, $CDCl_3$, 298 K) of **NB-PEG**

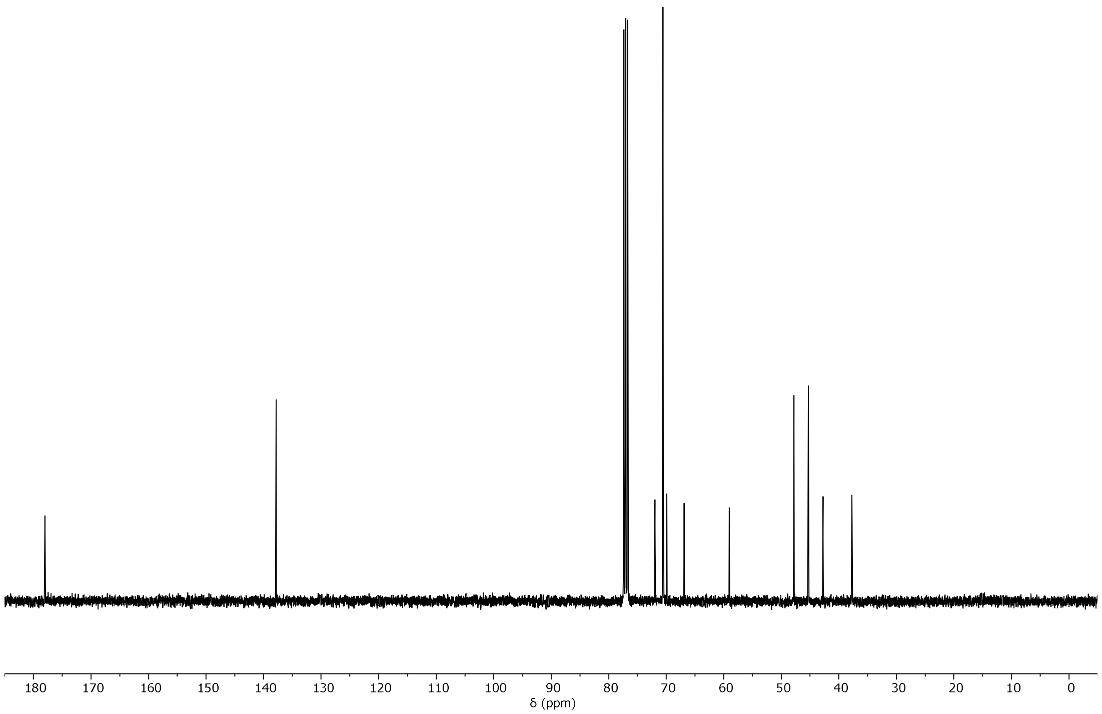

Spectrum S4 ^{13}C NMR (101 MHz, $CDCl_3$, 298 K) of **NB-PEG**

3.2 Synthesis of NB-NR$_4$

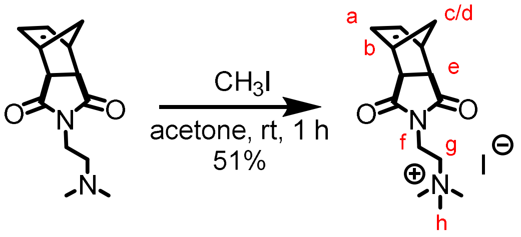

To a stirring solution of **NB-amine** (400 mg, 1.7 mmol, 1.0 eq.) in acetone (2.0 mL) was added iodomethane (2.0 mL, large excess), causing immediate precipitation. After five minutes the precipitate was filtered, washed with acetone (10 mL) and dried to afford **NB-NR$_4$** (434 mg, 0.87 mmol, 51 %) as a colourless solid.

^{1}H NMR (400 MHz, D_2O, 298 K) δ 6.38 (t, J = 1.7 Hz, 2H, H_a), 4.01 (t, J = 7.5 Hz, 2H, H_f), 3.56 (td, J = 7.4, 1.5 Hz, 2H, H_g), 3.30 – 3.25 (m, 2H, H_b), 3.24 (d, J = 1.5 Hz, 9H, H_h), 2.92 (d, J = 1.5 Hz, 2H, H_e), 1.55 (dd, J = 10.2, 1.6 Hz, 1H, $H_{c/d}$), 1.22 (d, J = 10.2 Hz, 1H, $H_{c/d}$).

^{13}C NMR (101 MHz, D_2O, 298 K) δ 180.55, 137.80, 61.60, 53.23, 47.99, 44.99, 42.29, 32.25.

HRMS (ESI$^+$) Calculated for $C_{14}H_{21}N_2O_2$ [M-I]$^+$ 249.1603, found 249.1608.

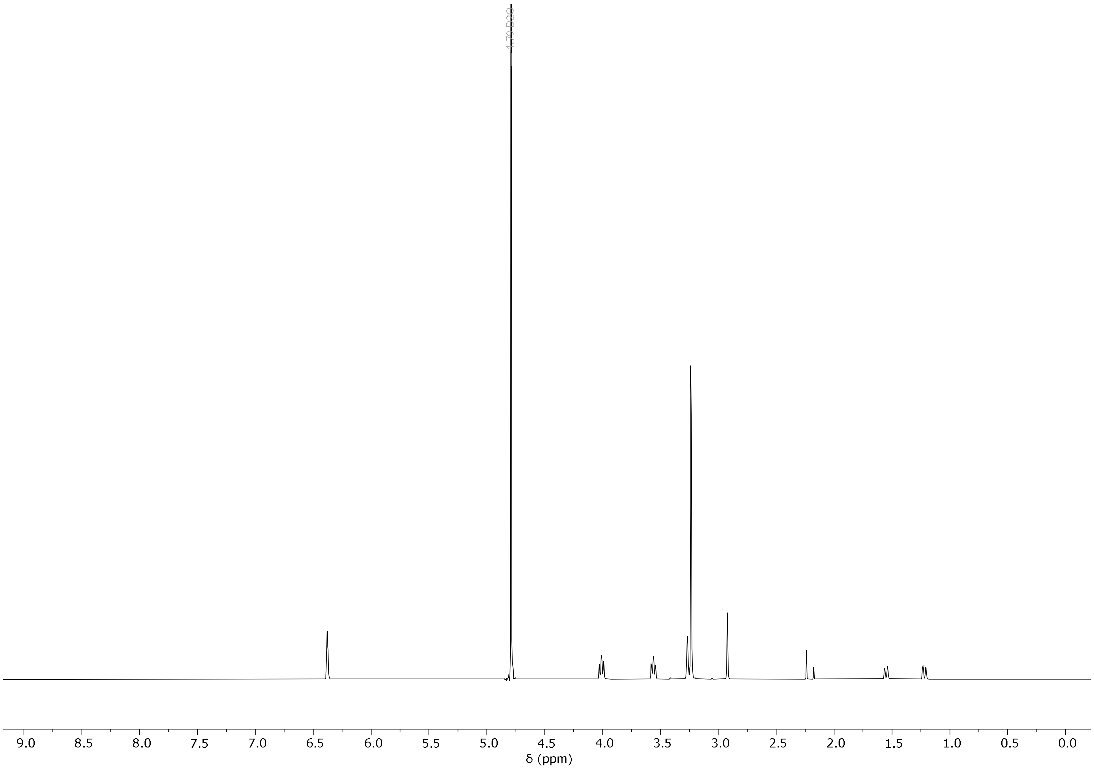

Spectrum S5 ^{1}H NMR (400 MHz, D_2O, 298 K) of **NB-NR$_4$**

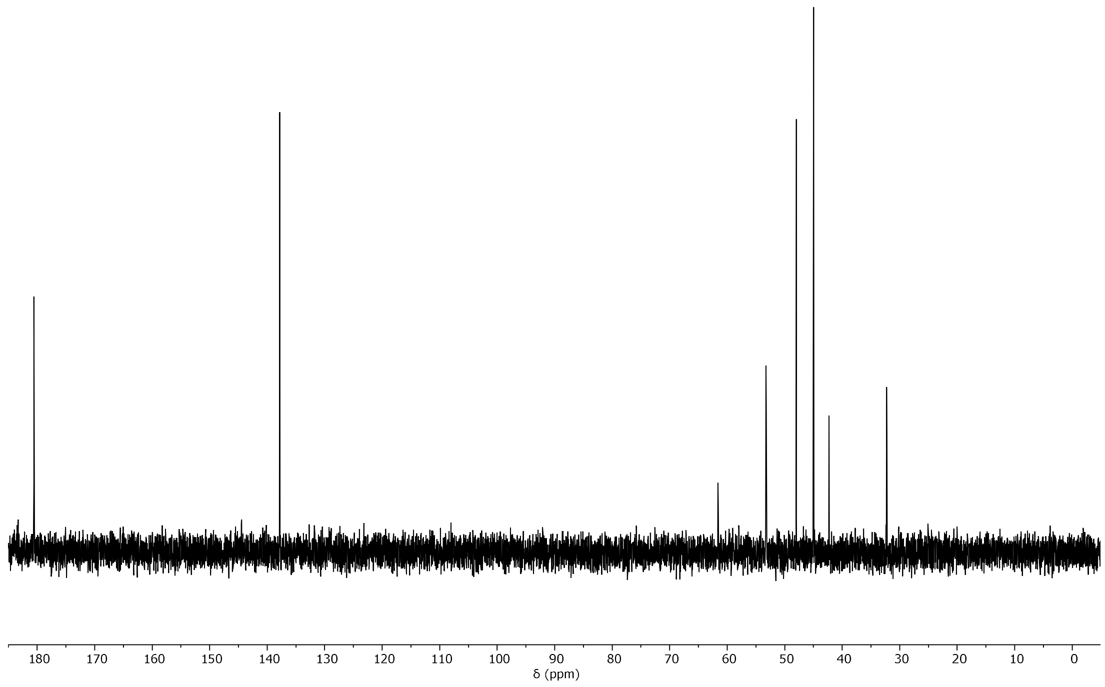

Spectrum S6 ^{13}C NMR (101 MHz, D_2O, 298 K) of **NB-NR$_4$**

3.3 Synthesis of NB-py

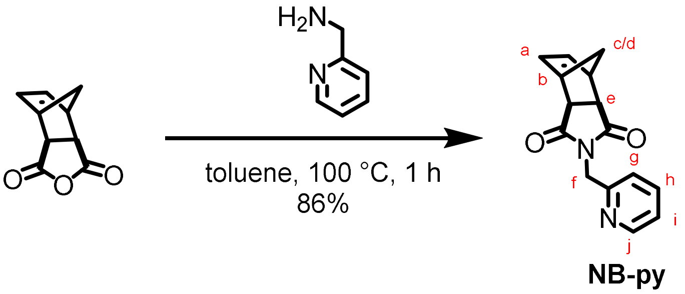

A stirring mixture of cis-5-Norbornene-exo-2,3-dicarboxylic anhydride (332 mg, 2.0 mmol, 1.0 eq.) and 2-(aminomethyl)pyridine (259 mg, 2.4 mmol, 1.2 eq.) in toluene (10 mL) was heated to 100 °C for one hour. The resulting solution was cooled and concentrated under reduced pressure. The residue was purified by flash column chromatography (SiO_2, EtOAc/hexane 1:4 to 2:3) to afford **NB-py** (440 mg, 1.73 mmol, 86%) as a colourless solid.

^{1}H NMR (400 MHz, $CDCl_3$, 298 K) δ 8.49 (ddd, *J* = 4.9, 1.8, 1.0 Hz, 1H, H_j), 7.62 (td, *J* = 7.7, 1.8 Hz, 1H, H_h), 7.25 (d, *J* = 8.1 Hz, 1H, H_g), 7.15 (ddd, *J* = 7.6, 4.9, 1.1 Hz, 1H, H_i), 6.29 (t, *J* = 1.9 Hz, 2H, H_a), 4.78 (s, 2H, H_f), 3.31 (t, *J* = 1.8 Hz, 2H, H, H_b), 2.76 (d, *J* = 1.4 Hz, 2H, H_e), 1.68 (dt, *J* = 9.8, 1.5 Hz, 1H, $H_{c/d}$), 1.48 (dt, *J* = 9.9, 1.6 Hz, 1H, $H_{c/d}$).

^{13}C NMR (101 MHz, $CDCl_3$, 298 K) δ 177.98, 154.72, 149.69, 138.16, 136.69, 122.60, 122.11, 48.13, 45.51, 43.59, 43.03.

HRMS Calculated for $C_{15}H_{15}N_2O_2$ $[M+H]^+$ 255.1134, found 255.1136.

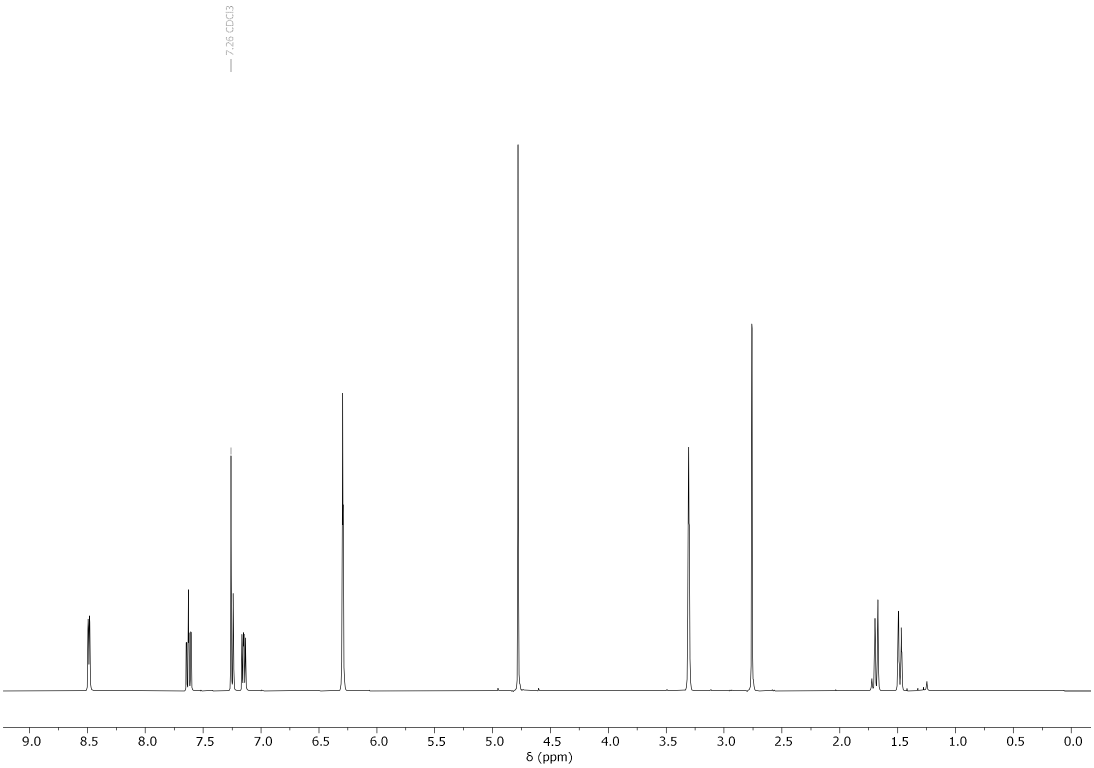

Spectrum S7 ^{1}H NMR (400 MHz, $CDCl_3$, 298 K) of **NB-py**

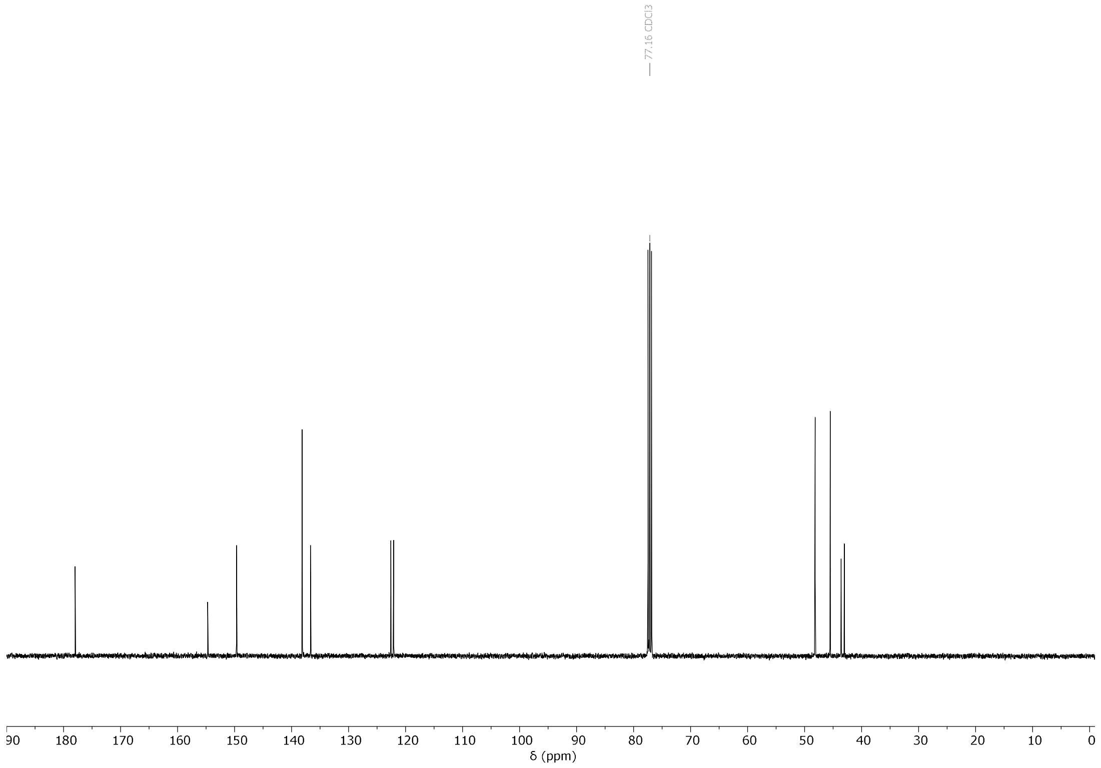

Spectrum S8 ^{13}C NMR (101 MHz, $CDCl_3$, 298 K) of **NB-py**

4. Synthesis and characterization of P(NB-PEG)$_{11}$-*b*-P(NB-amine)$_5$-*b*-P(NB-MEG)$_{200/300}$, P1$_{200}$ and P1$_{300}$

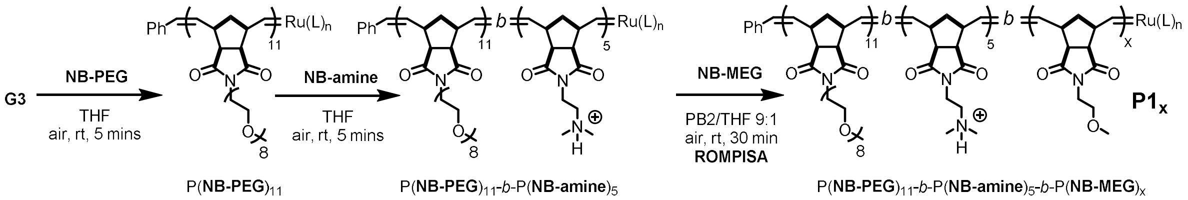

Scheme S2 Synthesis of **P1$_{200/300}$**.

A solution of **NB-PEG** (31.2 mg, 59 µmol, 13 eq.) in 530 µL of filtered THF was rapidly added to a solution of **G3** (3.8 mg, 5.2 µmol, 1.2 eq.) in 120 µL of THF contained within a glass vial equipped with a stirrer bar. The resulting solution was stirred rapidly for five minutes. After this time a 100 µL aliquot was removed for GPC analysis, leaving 1.0 eq. of P(**NB-PEG**)$_{11}$ in 550 µL of THF. To this solution was added **NB-amine** (5.2 mg, 22 µmol, 5.0 eq.) in 450 µL THF. The resulting solution was stirred for a further five minutes to give P(**NB-PEG**)$_{11}$-*b*-P(**NB-amine**)$_5$ macroinitiator (final concentration = 4.4 µmol/mL).

Two aliquots (50 µL, 0.22 µmol, for **P1$_{200}$** and 33 µL, 0.15 µmol, for **P1$_{300}$**) of the resulting solution of P(**NB-PEG**)$_{11}$-*b*-P(**NB-amine**)$_5$ in THF were dispensed into 2 mL glass vials containing a stirrer bar. Filtered THF was added to give 100 µL in total in each vial. A solution of **NB-MEG** (10 mg, 45 µmol, 200 eq. for **P1$_{200}$** and 300 eq. for **P1$_{300}$**) in 0.9 mL of acidic phosphate buffer (pH = 2, PB2, final solids concentration = 1 wt%) was added rapidly to each vial. The resulting solution was thoroughly mixed by drawing up the entire volume into the pipette tip and ejecting the liquid back into the vial three times. The ROMPISA polymerizations were stirred at 300 rpm for 30 minutes to give P(**NB-PEG**)$_{11}$-*b*-P(**NB-amine**)$_5$-*b*-P(**NB-MEG**)$_{200/300}$ (**P1$_{200}$** and **P1$_{300}$** respectively). These were analyzed by ^{1}H NMR, GPC, DLS, TEM and SAXS (for **P1$_{200}$** only, section 10). DSC was performed on freeze dried **P1$_{200}$** (section 6).

Characterization Summary

Polymer	$M_{n,theo}$ /kDa	$M_{n,GPC}$ /kDa	$Đ_{GPC}$	$Z_{avg,DLS}$ /nm	PD_{DLS}	L_{TEM} /nm
P(**NB-PEG**)$_{11}$	6.5	6.7	1.11	n/a	n/a	n/a
P(**NB-PEG**)$_{11}$-*b*-P(**NB-amine**)$_5$	7.7	8.1	1.11	n/a	n/a	n/a
**P1$_{200}$ @ pH 2**	52	52	1.09	49	0.09	36±6
**P1$_{300}$ @ pH 2**	74	69	1.09	98	0.17	97±47

^{1}H NMR

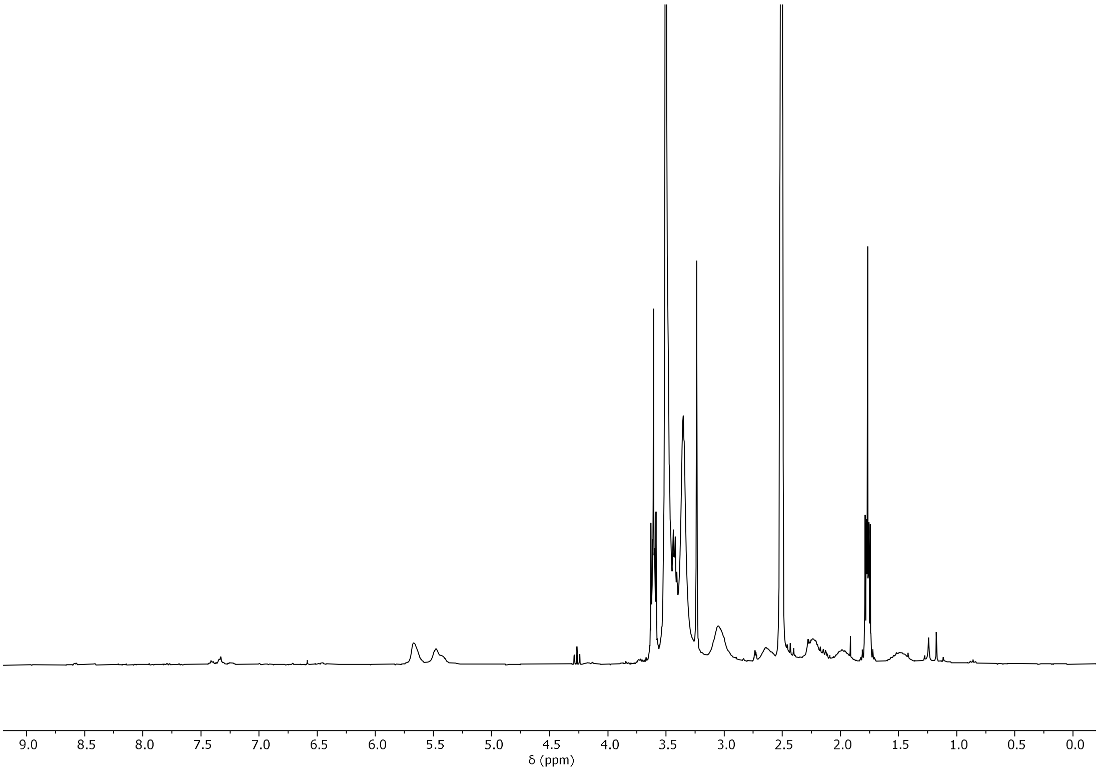

Spectrum S9 ^{1}H NMR (300 MHz, $(CD_3)_2SO$, 300 K) of P(**NB-PEG**)$_{11}$-*b*-P(**NB-amine**)$_5$ + THF

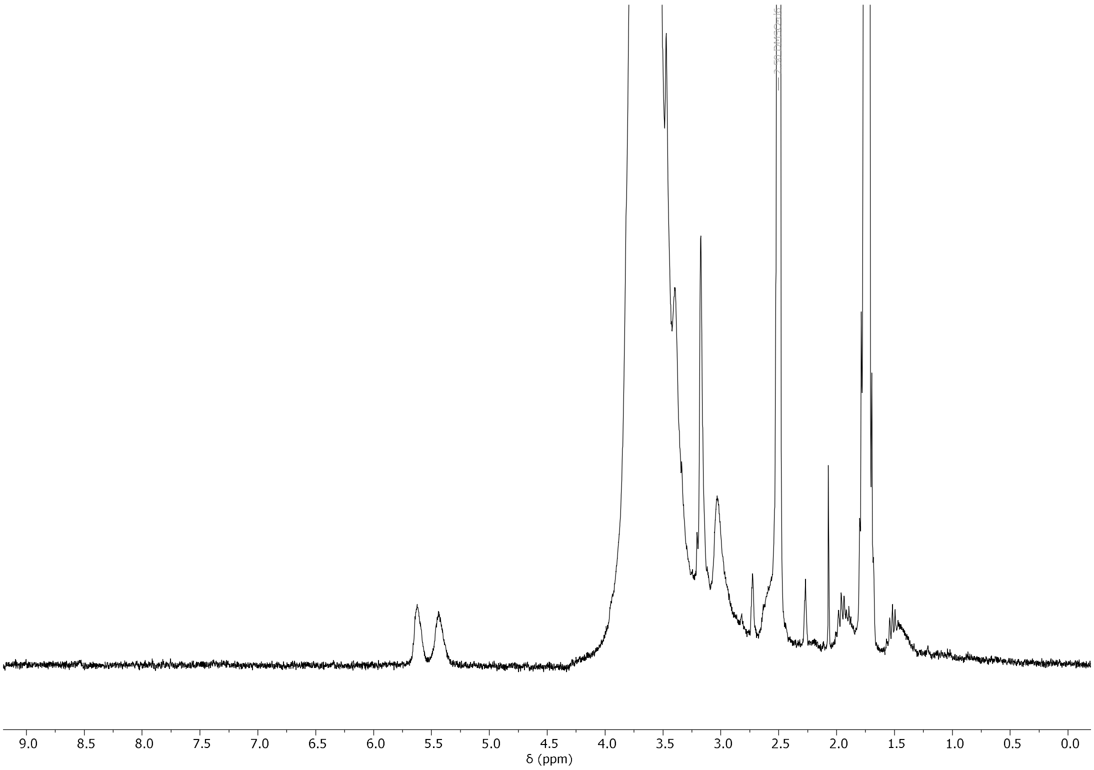

Spectrum S10 ^{1}H NMR (300 MHz, $(CD_3)_2SO$, 300 K) of $\mathbf{P1_{200}}$ + THF/PB2

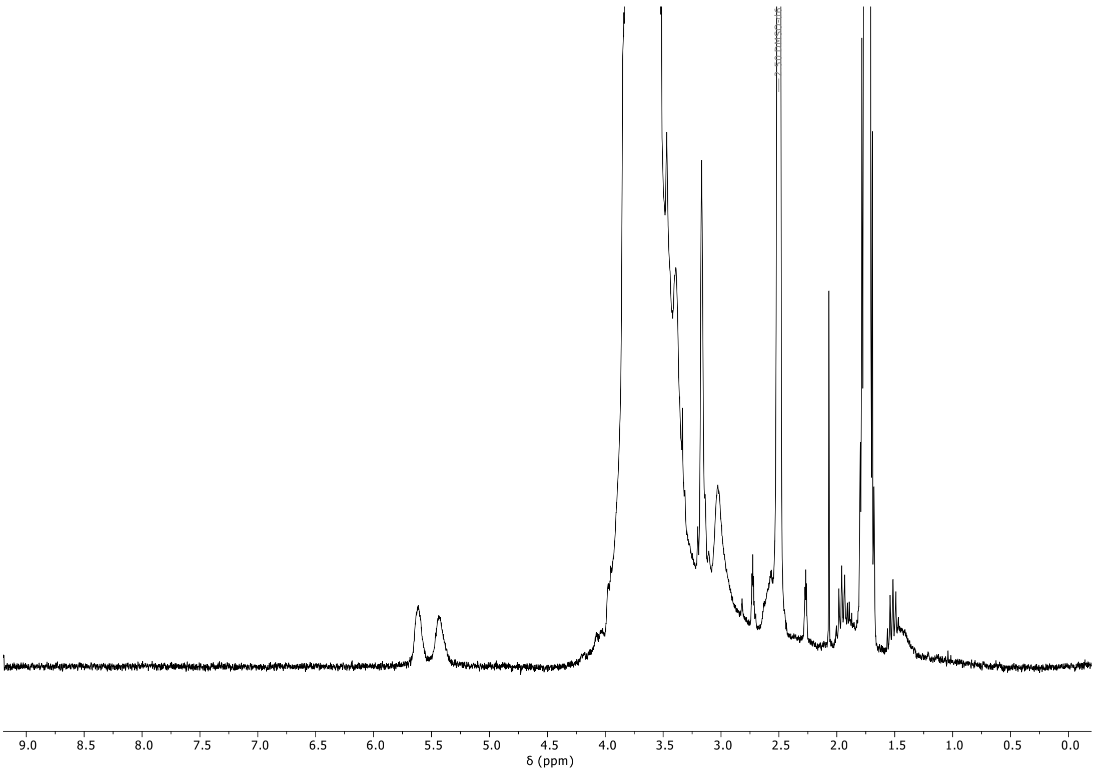

Spectrum S11 ^{1}H NMR (300 MHz, $(CD_3)_2SO$, 300 K) of **P1$_{300}$** + THF/PB2

GPC

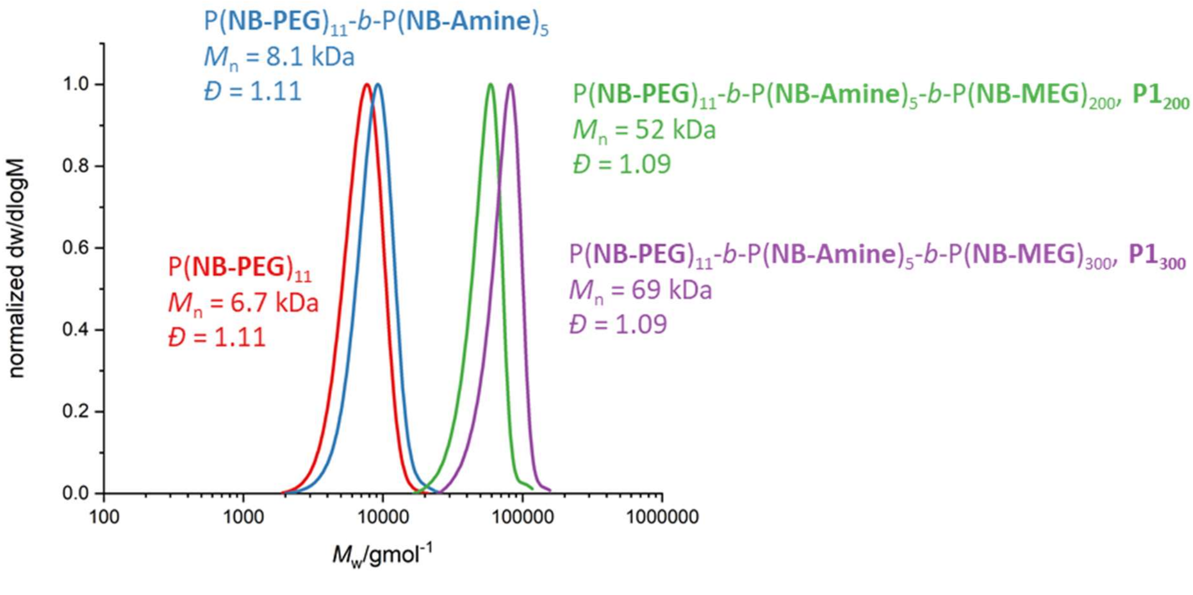

Figure S2 Normalized GPC trace (THF eluent, PMMA standards) of **P1$_{200}$/P1$_{300}$** and intermediate polymers.

Characterization of $P1_{200}$ and $P1_{300}$ particles at pH 2

DLS

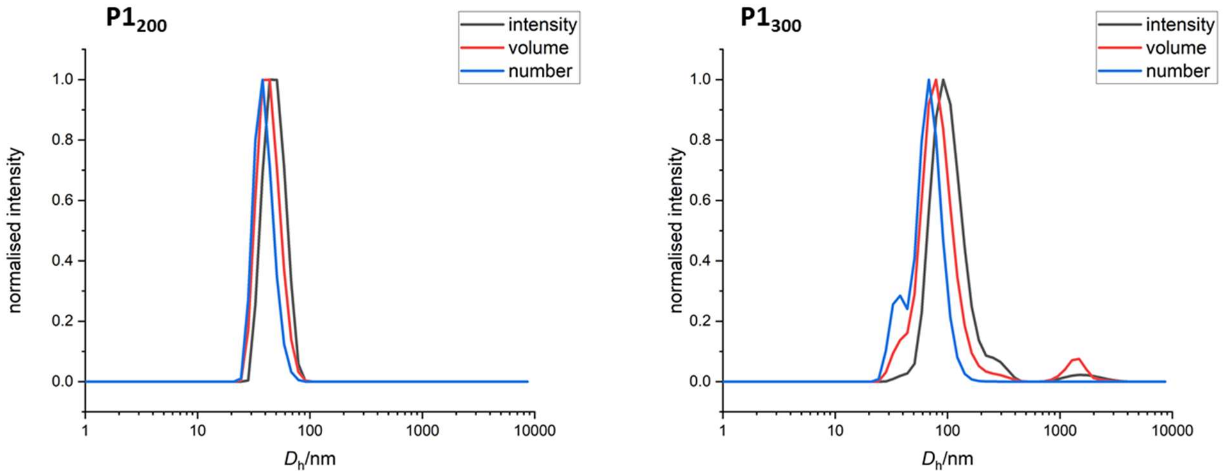

Figure S3 DLS traces of $\mathbf{P1_{200}}$ and $\mathbf{P1_{300}}$ particles as synthesized at pH 2

Dry-state TEM

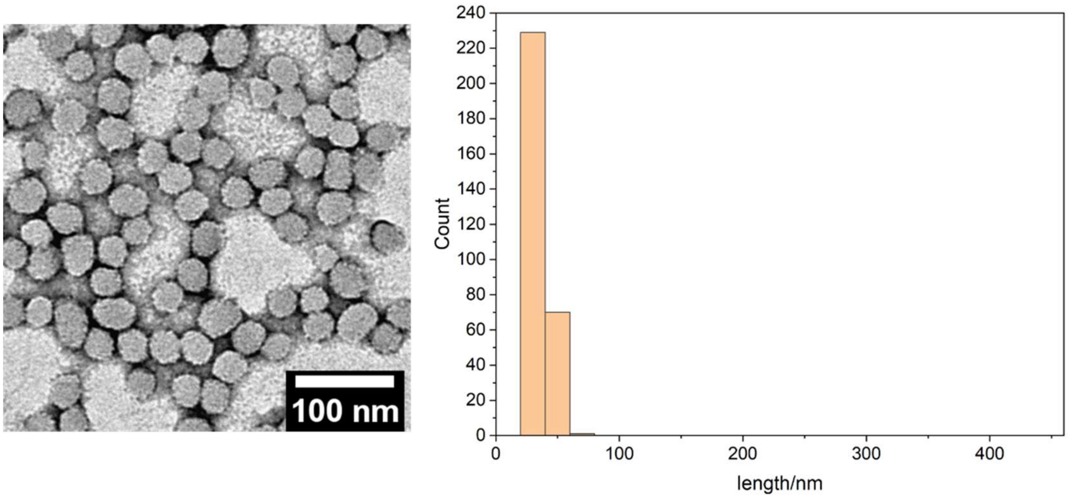

Figure S4 Dry-state TEM image and histogram (300 particles analyzed) of $\mathbf{P1_{200}}$ particles as synthesized at pH 2.

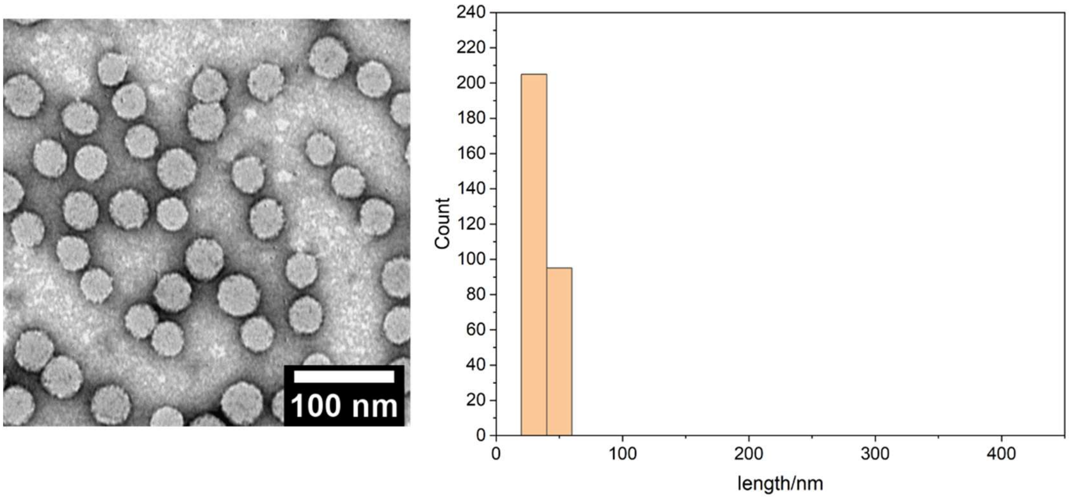

Figure S5 Dry-state TEM image and histogram (300 particles analyzed) of **P1$_{200}$** particles **aged for three months** at pH 2.

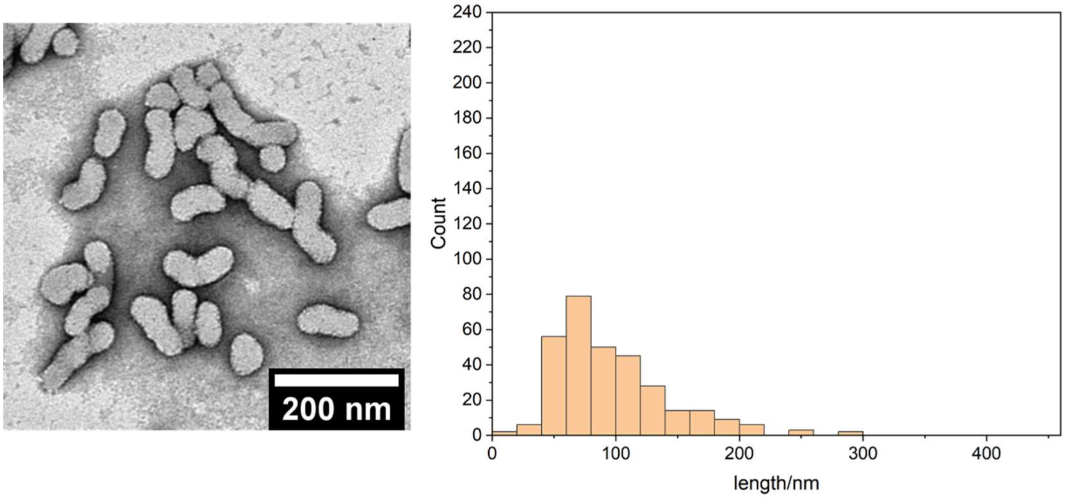

Figure S6 Dry-state TEM image and histogram (300 particles analyzed) of **P1$_{300}$** particles as synthesized at pH 2.

Cryo-TEM

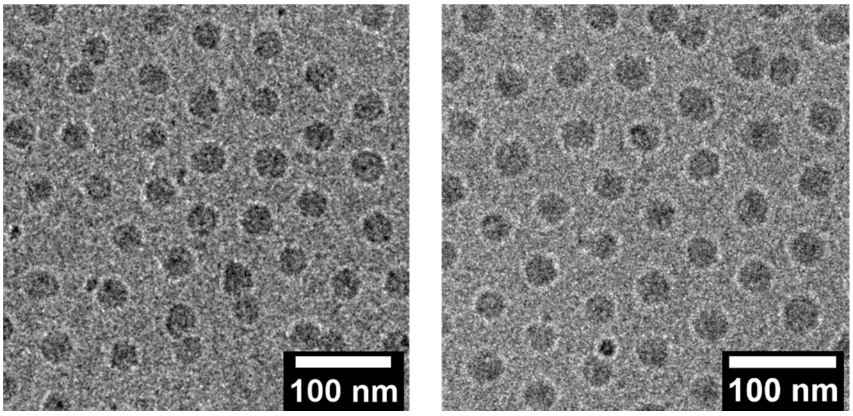

Figure S7 Cryo-TEM images of **P1$_{200}$** particles as synthesized at pH 2.

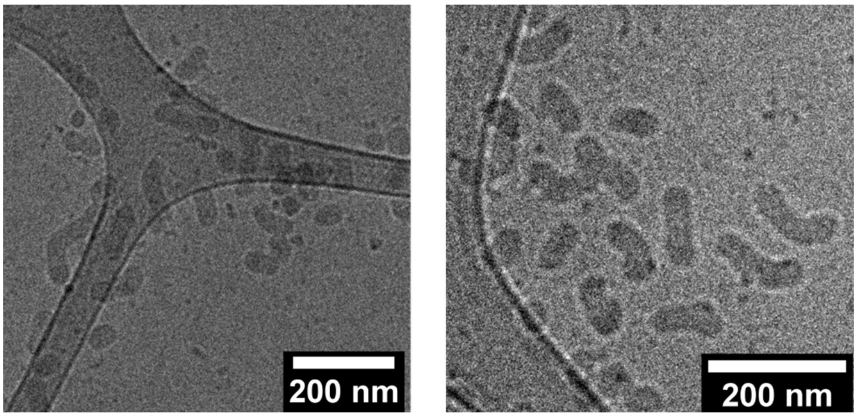

Figure S8 Cryo-TEM images of **P1$_{300}$** particles as synthesized at pH 2.

5. Synthesis and characterization of P(NB-PEG)$_{11}$-*r*-P(NB-amine)$_5$-*b*-P(NB-MEG)$_{200/300}$, P2$_{200}$ and P2$_{300}$

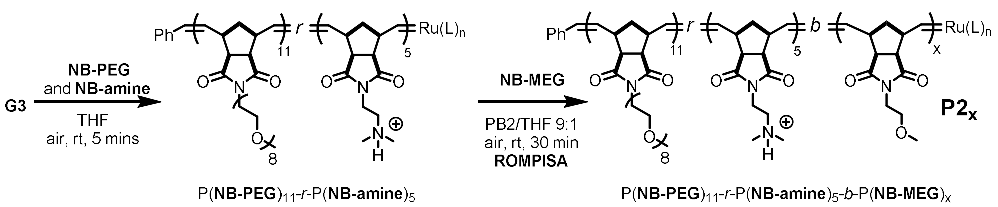

Scheme S3 Synthesis of **P2$_{200/300}$**.

A solution of **NB-PEG** (26.4 mg, 50 µmol, 11 eq.) and **NB-amine** (5.2 mg, 22 µmol, 5.0 eq.) in 900 µL of filtered THF was rapidly added to a solution of **G3** (3.2 mg, 4.4 µmol, 1.0 eq.) in 100 µL of THF contained within a glass vial equipped with a stirrer bar. The resulting solution was stirred rapidly for five minutes to give P(**NB-PEG**)$_{11}$-*r*-P(**NB-amine**)$_5$ macroinitiator (final concentration = 4.4 µmol/mL).

Two aliquots (50 µL, 0.22 µmol, for **P2$_{200}$** and 33 µL, 0.15 µmol, for **P2$_{300}$**) of the resulting solution of P(**NB-PEG**)$_{11}$-*r*-P(**NB-amine**)$_5$ in THF were dispensed into 2 mL glass vials containing a stirrer bar. Filtered THF was added to give 100 µL in total in each vial. A solution of **NB-MEG** (10 mg, 45 µmol, 200 eq. for **P2$_{200}$** and 300 eq. for **P2$_{300}$**) in 0.9 mL of acidic phosphate buffer (pH = 2, PB2, final solids concentration = 1 wt%) was added rapidly to each vial. The resulting solution was thoroughly mixed by drawing up the entire volume into the pipette tip and ejecting the liquid back into the vial three times. The ROMPISA polymerizations were stirred at 300 rpm for 30 minutes to give P(**NB-PEG**)$_{11}$-*r*-P(**NB-amine**)$_5$-*b*-P(**NB-MEG**)$_{200/300}$ (**P2$_{200}$** and **P2$_{300}$** respectively). These were analyzed by ^{1}H NMR, GPC, DLS and TEM. DSC was performed on freeze dried **P2$_{200}$** (section 6).

Characterization Summary

Polymer	$M_{n,theo}$ /kDa	$M_{n,GPC}$ /kDa	$Đ_{GPC}$	$Z_{avg,DLS}$ /nm	PD_{DLS}	L_{TEM} /nm
P(**NB-PEG**)$_{11}$-r-P(**NB-amine**)$_5$	7.7	8.3	1.11	n/a	n/a	n/a
**P2$_{200}$ @ pH 2**	52	50	1.08	47	0.10	37±6
**P2$_{300}$ @ pH 2**	74	66	1.10	80	0.07	81±33

^{1}H NMR

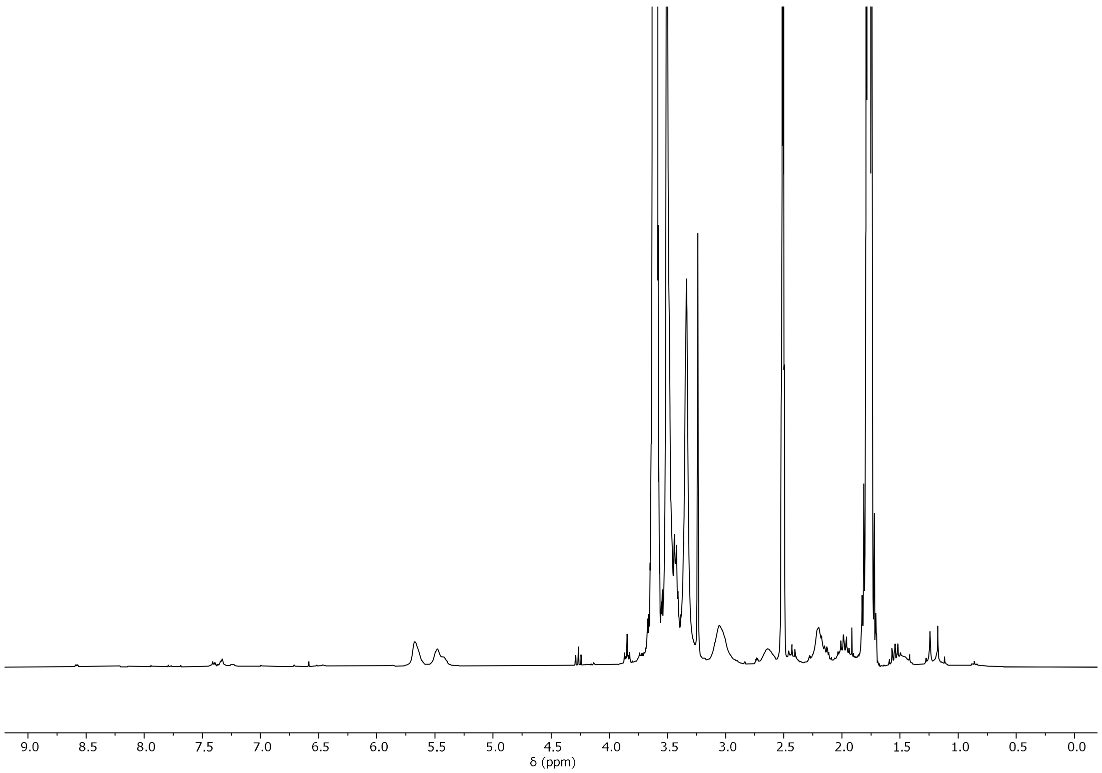

Spectrum S12 ^{1}H NMR (300 MHz, $(CD_3)_2SO$, 300 K) of P(**NB-PEG**)$_{11}$-*r*-P(**NB-amine**)$_5$ + THF

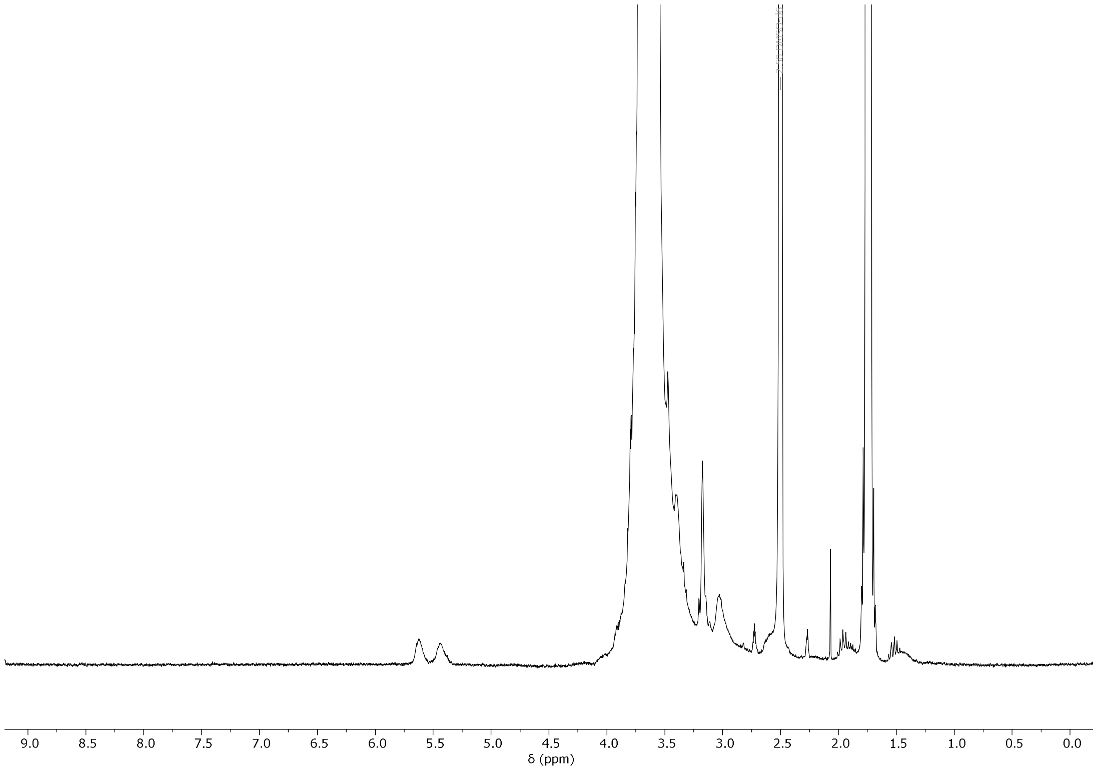

Spectrum S13 Partial ^{1}H NMR (300 MHz, $(CD_3)_2SO$, 300 K) of **P2$_{200}$** + THF/PB2

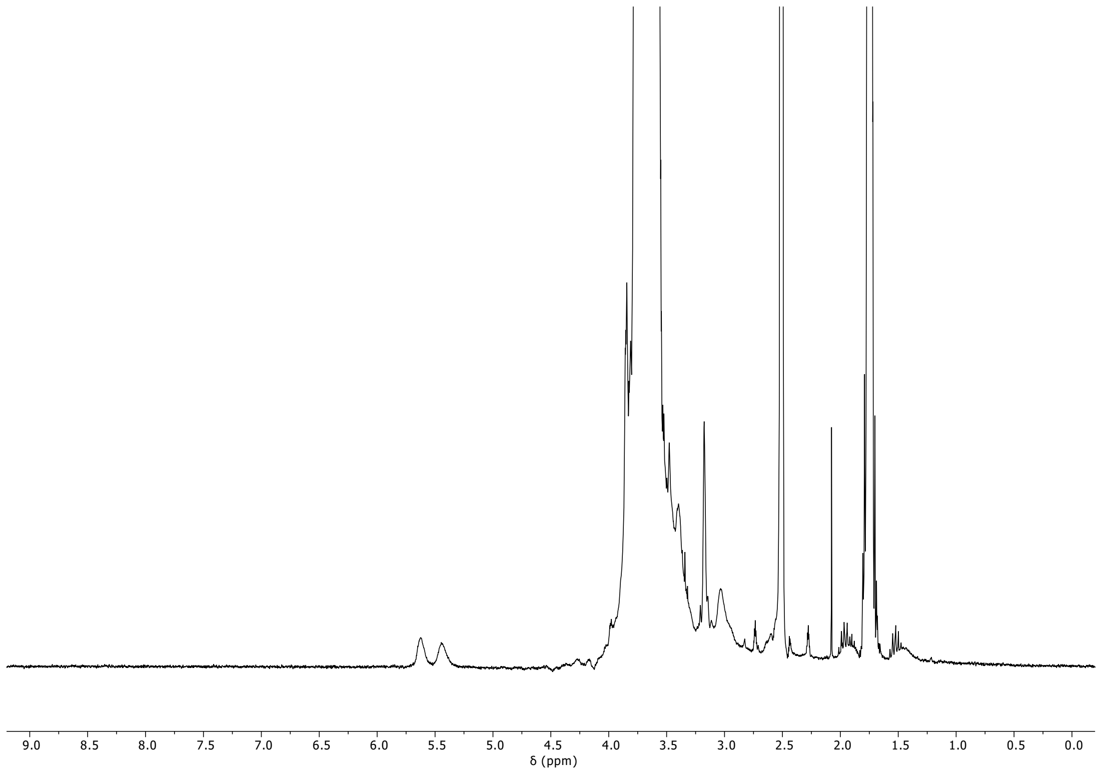

Spectrum S14 Partial ^{1}H NMR (300 MHz, $(CD_3)_2SO$, 300 K) of **$P2_{300}$** + THF/PB2

GPC

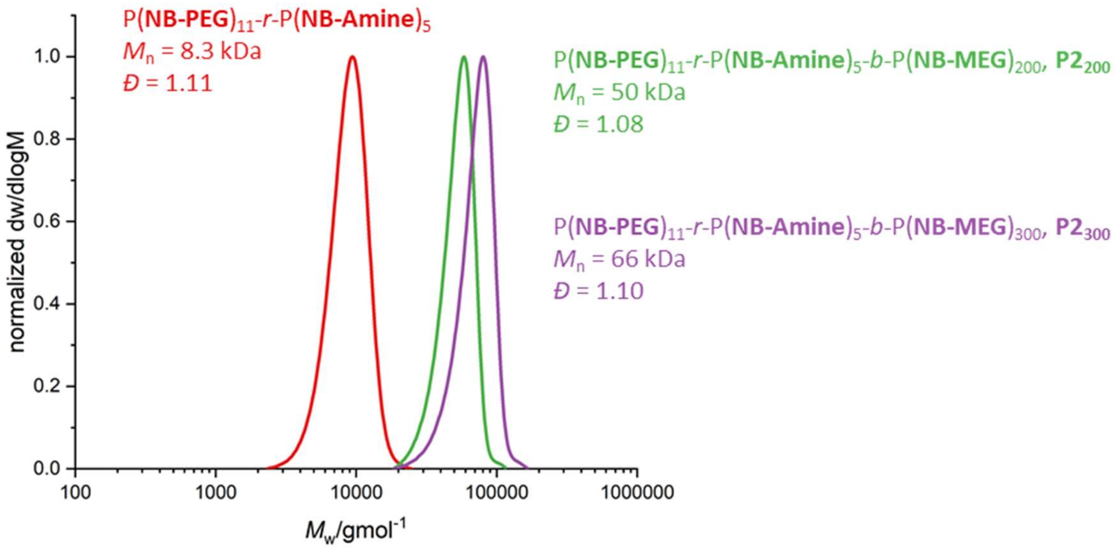

Figure S9 Normalized GPC trace (THF eluent, PMMA standards) of **$P2_{200}$/$P2_{300}$** and intermediate polymer.

Characterization of $P2_{200}$ and $P2_{300}$ particles at pH 2

DLS

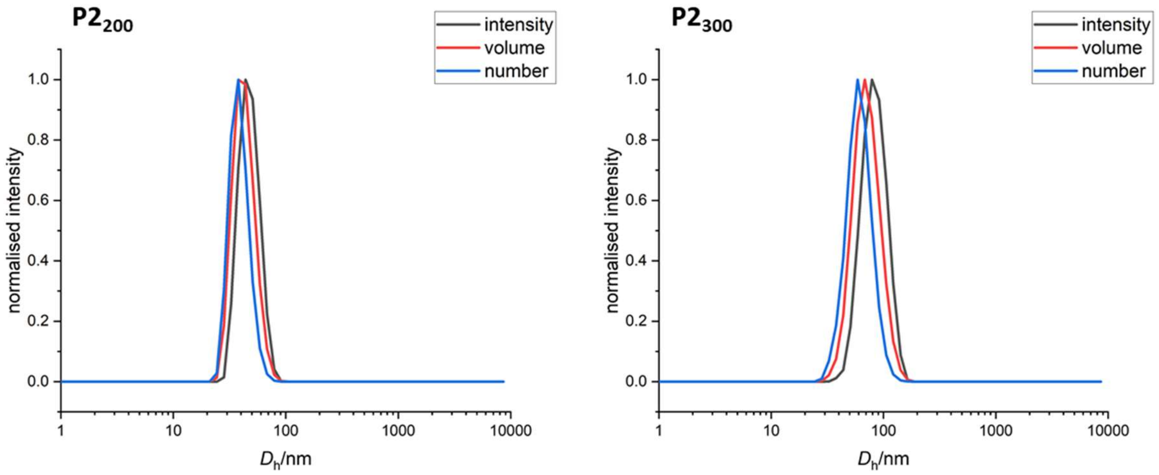

Figure S10 DLS traces of $\mathbf{P2_{200}}$ and $\mathbf{P2_{300}}$ particles as synthesized at pH 2.

Dry-state TEM

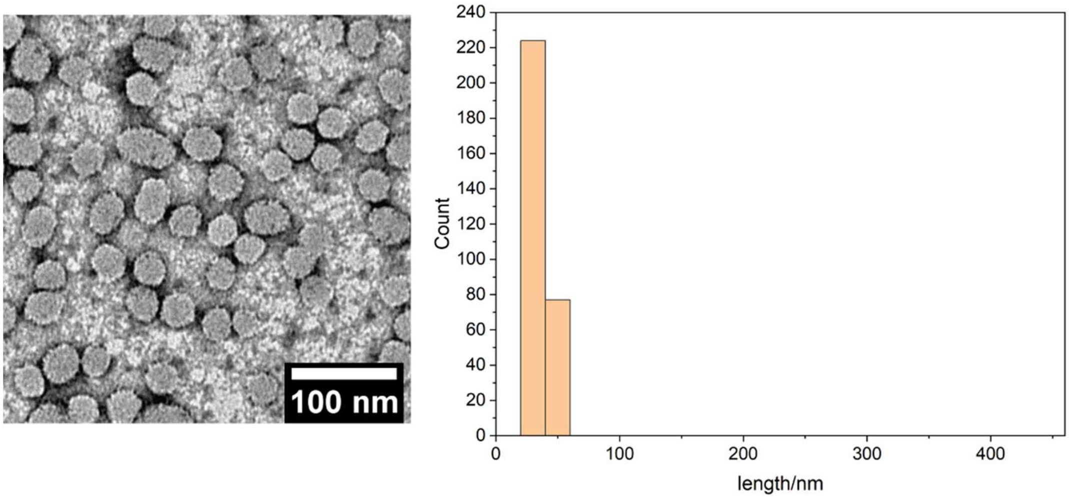

Figure S11 Dry-state TEM image and histogram (300 particles analyzed) of $\mathbf{P2_{200}}$ particles as synthesized at pH 2.

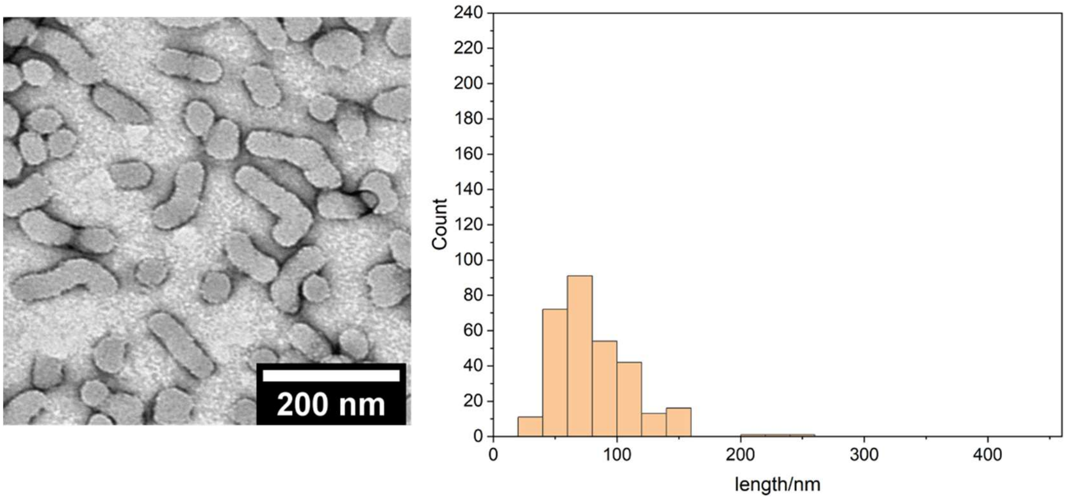

Figure S12 Dry-state TEM image and histogram (300 particles analyzed) of **P2$_{300}$** particles as synthesized at pH 2.

Cryo-TEM

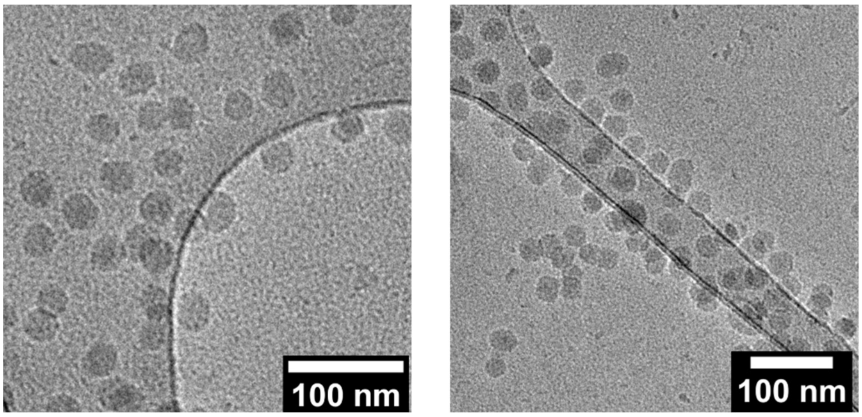

Figure S13 Cryo-TEM images of **P2$_{200}$** particles as synthesized at pH 2.

6. Triggered fusion of P1$_{200}$ and P2$_{200}$

To 100 µL solution of **P1$_{200}$** or **P2$_{200}$** (as synthesized, 10 vol% THF in PB2) was rapidly added 300 µL of NaOH solution (100 mM, 10 vol% THF). The resulting solution was thoroughly mixed by drawing up the entire volume into the pipette tip and ejecting the liquid back into the vial three times. The resulting solution was analyzed by GPC, DLS, TEM and SAXS (for **P1$_{200}$** only, see section 10).

Characterization Summary

Polymer	$Z_{avg,DLS}$ /nm	PD_{DLS}	L_{TEM} /nm
**P1$_{200}$** @ pH 12	166	0.18	99±63
**P2$_{200}$** @ pH 12	303	0.24	aggregation

GPC

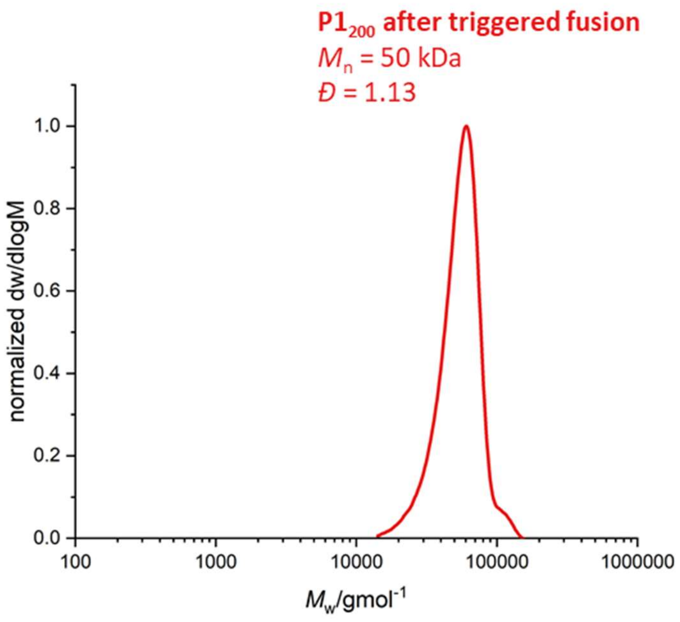

Figure S14 Normalized GPC trace (THF eluent, PMMA standards) of **P1$_{200}$** after triggered fusion.

DLS

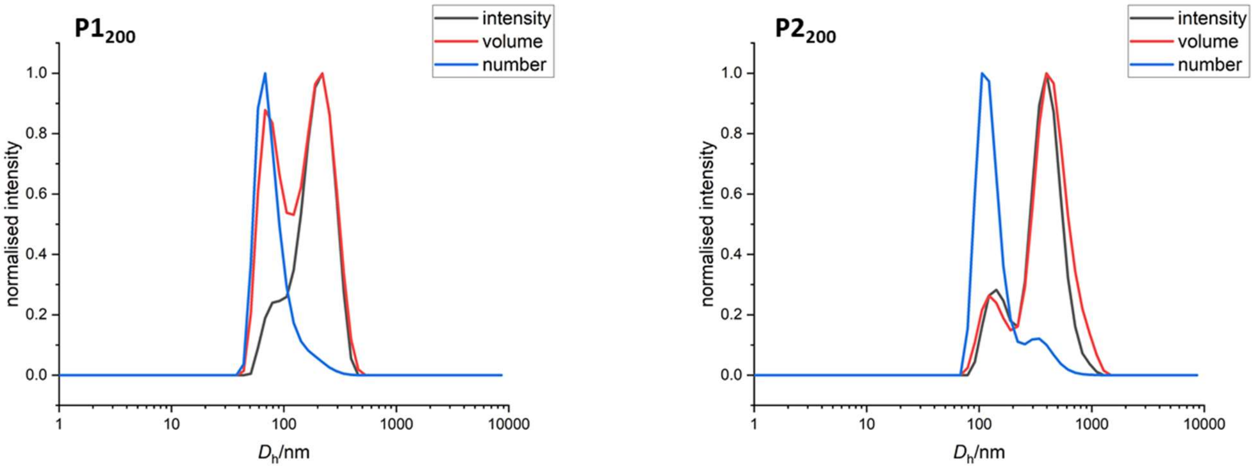

Figure S15 DLS traces of **P1$_{200}$** and **P2$_{200}$** after triggered fusion.

Dry-state TEM

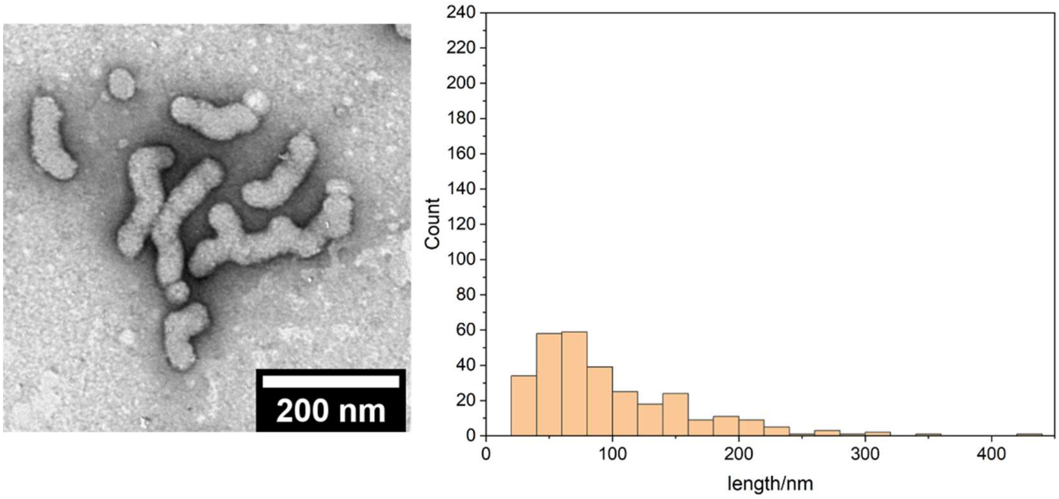

Figure S16 Dry-state TEM image and histogram (300 particles analyzed) of **P1$_{200}$** after triggered fusion.

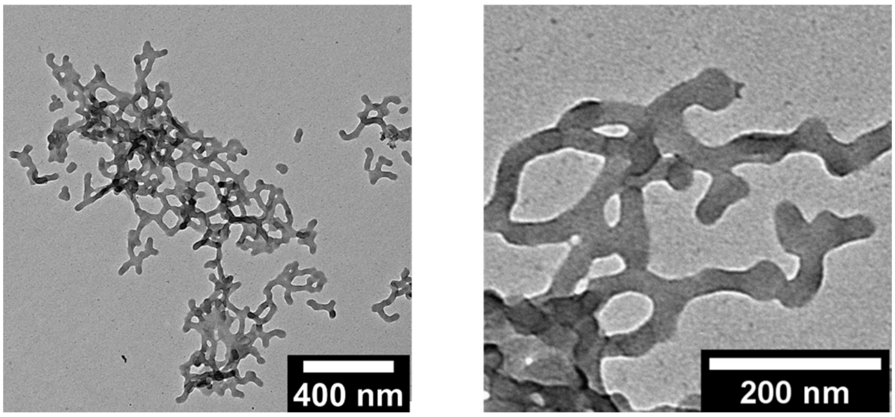

Figure S17 Dry-state TEM images of **P2$_{200}$** particles after triggered fusion.

Cryo-TEM

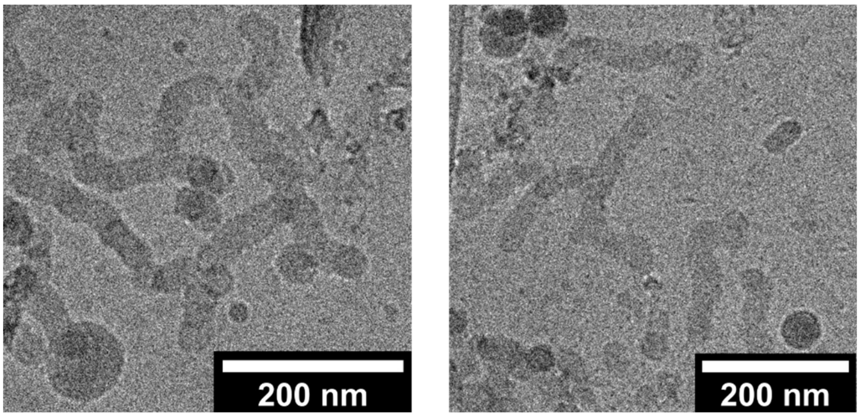

Figure S18 Cryo-TEM images of **P1**$_{200}$ particles after triggered fusion.

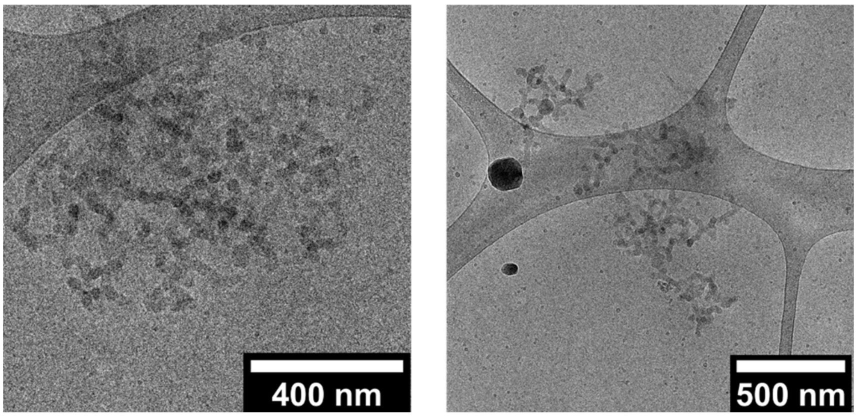

Figure S19 Cryo-TEM images of **P2**$_{200}$ particles after triggered fusion.

DSC

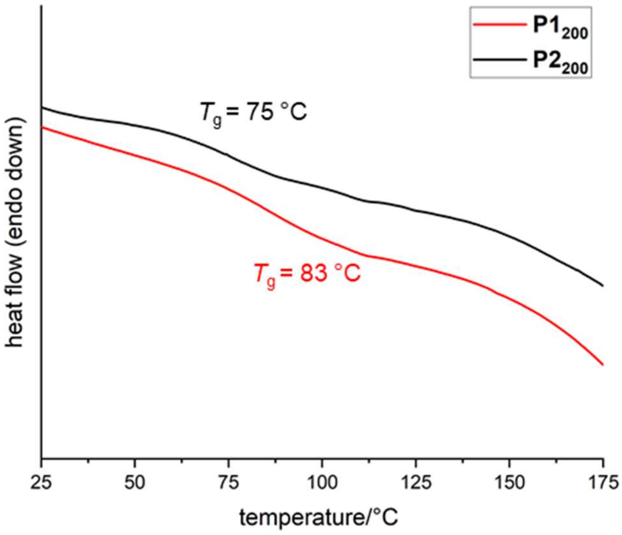

Figure S20 DSC thermogram of **P1**$_{200}$ and **P2**$_{200}$

Reacidification of fused $\mathbf{P1_{200}}$

To 100 µL solution of fused $\mathbf{P1_{200}}$ (0.25 wt%, fusion triggered as above) at pH 12 was added 300 µL of PB2 solution (100 mM, 10 vol% THF) to readjust the pH to 2. After five minutes the resulting solution was analyzed by DLS and TEM.

Polymer	$Z_{avg,DLS}$ /nm	PD_{DLS}	L_{TEM} /nm
$\mathbf{P1_{200}}$ reacidified	208	0.25	94±51

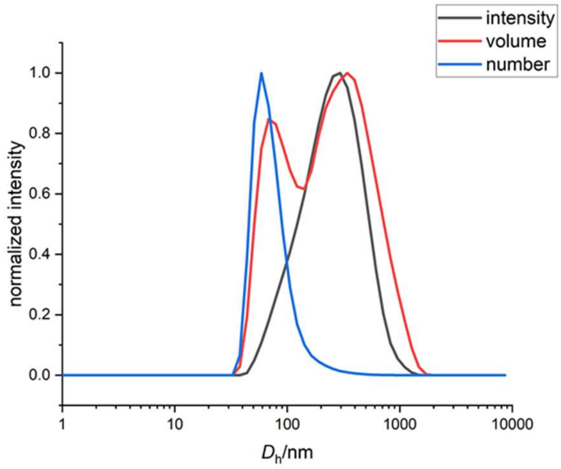

Figure S21 DLS traces of $\mathbf{P1_{200}}$ after reacidification.

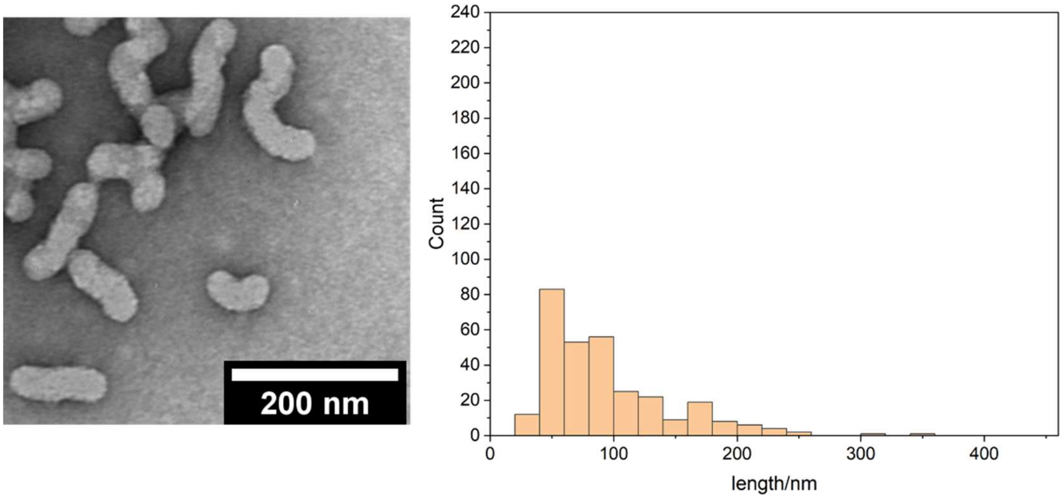

Figure S22 Dry-state TEM images of $\mathbf{P1_{200}}$ after reacidification.

7. Synthesis of P(NB-PEG)$_{11}$-*b*-P(NB-NR$_4$)$_5$-*b*-P(NB-MEG)$_{200}$, P3$_{200}$

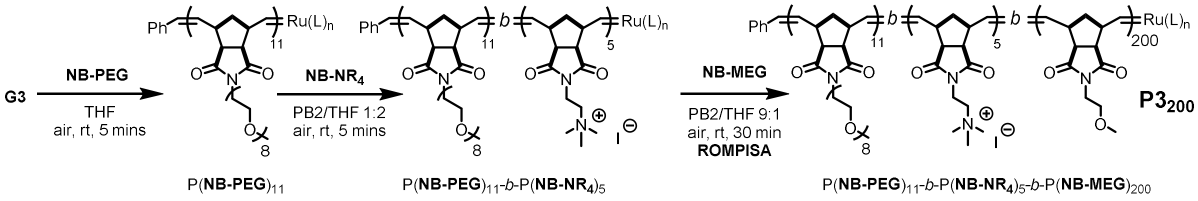

Scheme S4 Synthesis of **P3$_{200}$**.

A solution of **NB-PEG** (31.2 mg, 59 µmol, 13 eq.) in 530 µL of filtered THF was rapidly added to a solution of **G3** (3.8 mg, 5.2 µmol, 1.2 eq.) in 120 µL of THF contained within a glass vial equipped with a stirrer bar. The resulting solution was stirred rapidly for five minutes. After this time a 100 µL aliquot was removed for GPC analysis, leaving 1.0 eq. of P(**NB-PEG**)$_{11}$ in 550 µL of THF. To this solution was added **NB-NR$_4$** (8.4 mg, 22 µmol, 5.0 eq.) in 450 µL of acidic phosphate buffer (pH = 2, PB2). The resulting solution was stirred for a further five minutes to give P(**NB-PEG**)$_{11}$-*b*-P(**NB-NR$_4$**)$_5$ macroinitiator (final concentration = 4.4 µmol/mL).

An aliquot (50 µL, 0.22 µmol) of the resulting solution of P(**NB-PEG**)$_{11}$-*b*-P(**NB-NR$_4$**)$_5$ was dispensed into 2 mL glass vials containing a stirrer bar. Filtered THF and PB2 were added to give 100 µL of each solvent in total. A solution of **NB-MEG** (10 mg, 45 µmol, 200 eq.) in 0.8 mL of acidic phosphate buffer (pH = 2, PB2, final solids concentration = 1 wt%) was added rapidly. The resulting solution was thoroughly mixed by drawing up the entire volume into the pipette tip and ejecting the liquid back into the vial. The ROMPISA polymerizations were stirred at 300 rpm for 30 minutes to give P(**NB-PEG**)$_{11}$-*b*-P(**NB-NR$_4$**)$_5$-*b*-P(**NB-MEG**)$_{200}$, **P2$_{200}$**. This was analyzed by ^{1}H NMR, GPC, DLS and TEM.

Characterization Summary

Polymer	$M_{n,theo}$ /kDa	$M_{n,GPC}$ /kDa	$Đ_{GPC}$	$Z_{avg,DLS}$ /nm	PD_{DLS}	L_{TEM} /nm
P(**NB-PEG**)$_{11}$	6.6	7.3	1.21	n/a	n/a	n/a
P(**NB-PEG**)$_{11}$-*b*-P(**NB- NR$_4$**)$_5$	8.4	15	1.30	n/a	n/a	n/a
**P3$_{200}$ @ pH 2**	52	64	1.31	57	0.09	42±9
**P3$_{200}$ @ pH 12**	n/a	n/a	n/a	58	0.06	46±10

^{1}H NMR

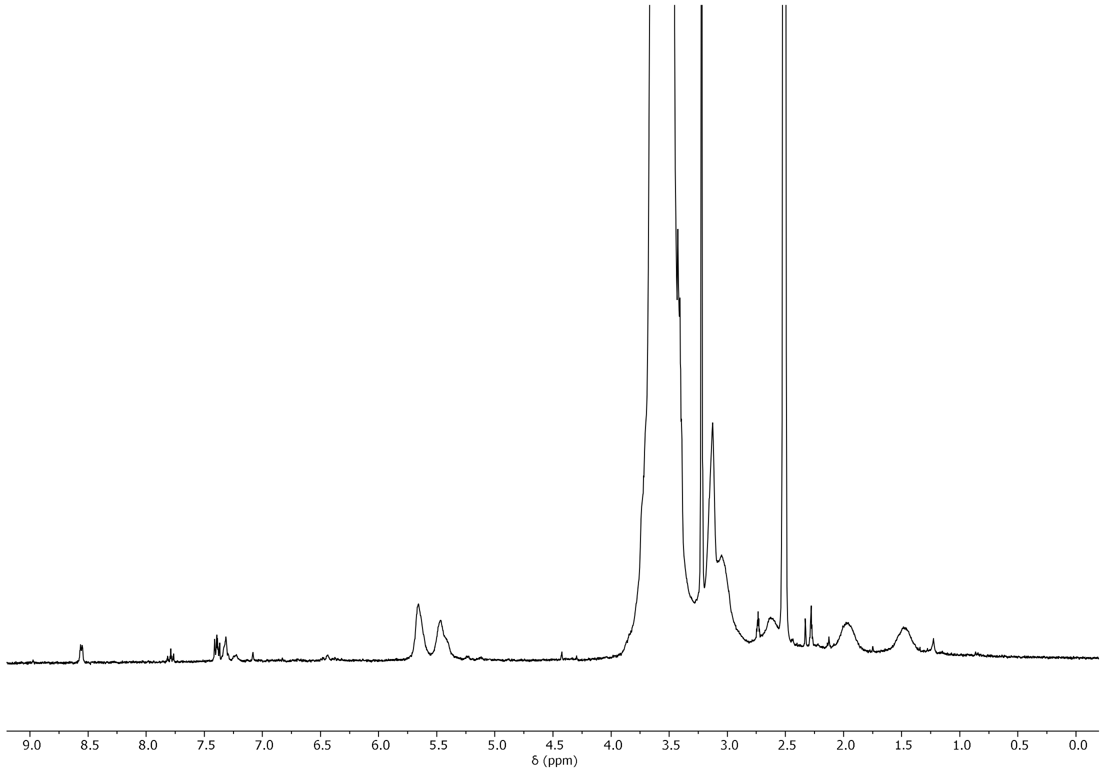

Spectrum S15 Partial ^{1}H NMR (300 MHz, $(CD_3)_2SO$, 300 K) of P(**NB-PEG**)$_{11}$-*b*-P(**NB- NR$_4$**)$_5$ + THF

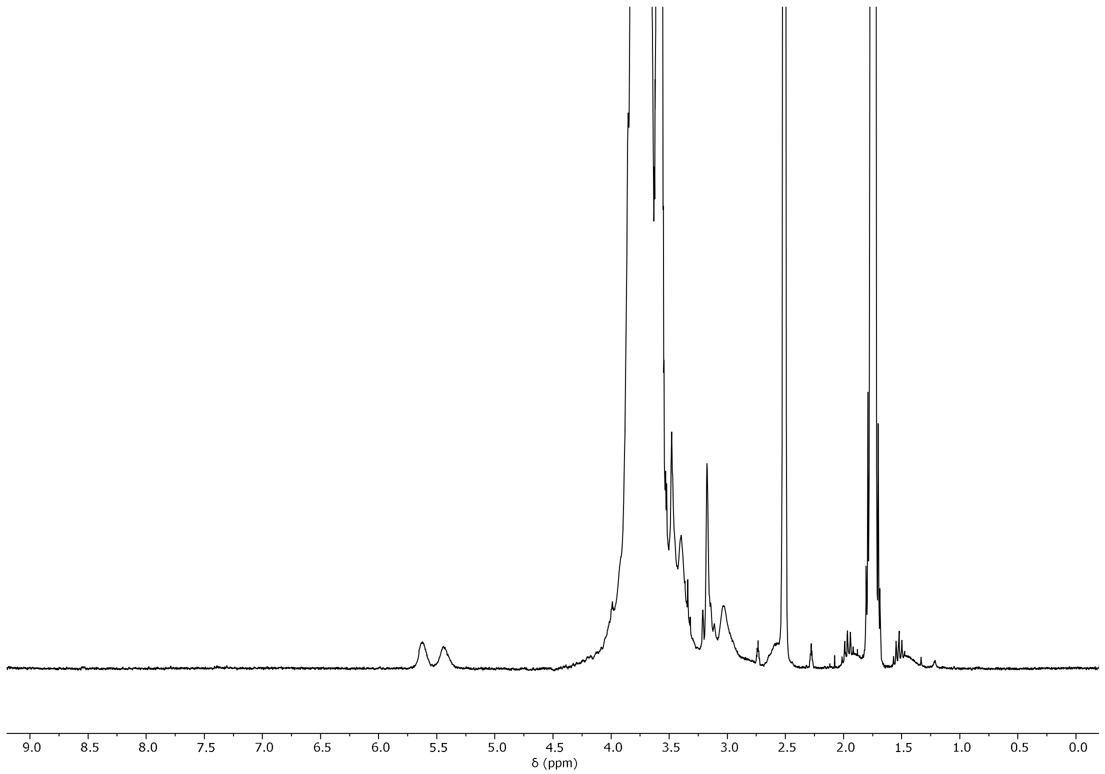

Spectrum S16 ^{1}H NMR (300 MHz, $(CD_3)_2SO$, 300 K) of **P3$_{200}$** + PB2/THF

GPC

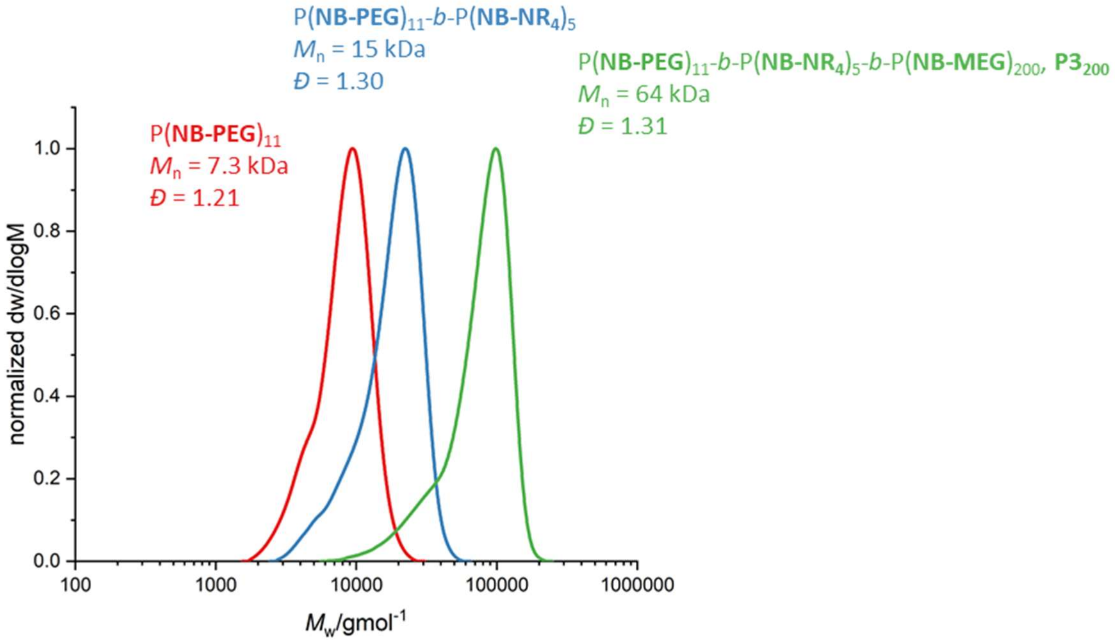

Figure S23 Normalized GPC trace (DMF eluent, PMMA standards) of $\mathbf{P3_{200}}$ and intermediate polymers.

DLS

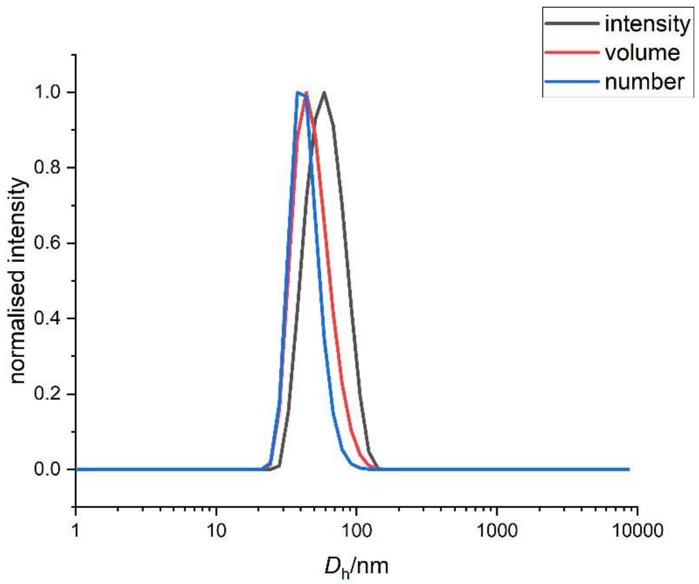

Figure S24 DLS traces of $\mathbf{P3_{200}}$ particles as synthesized at pH 2.

Dry-state TEM

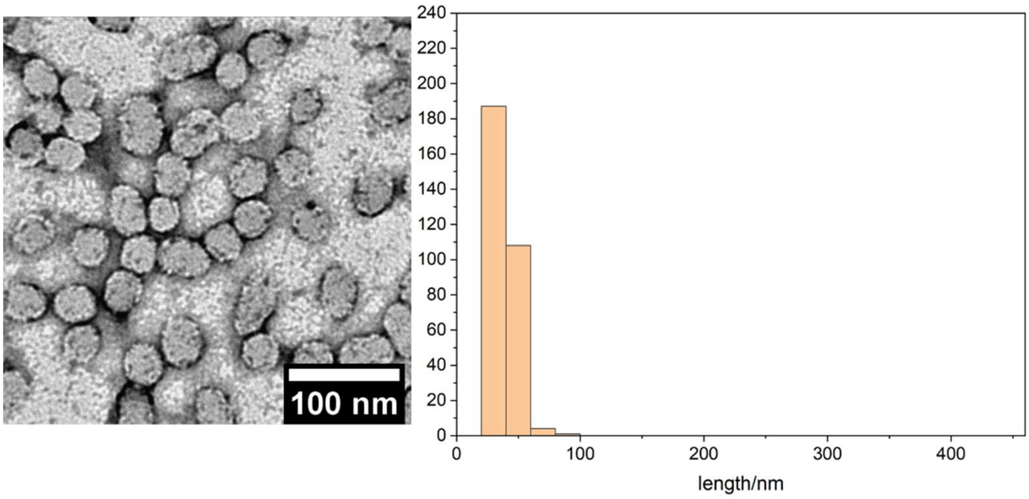

Figure S25 Dry-state TEM image and histogram (300 particles analyzed) of $\mathbf{P3_{200}}$ as synthesised at pH 2.

Attempted triggered fusion of $P3_{200}$

To 100 µL solution of $\mathbf{P3_{200}}$ (as synthesized, 10 vol% THF in PB2) was rapidly added 300 µL of NaOH solution (100 mM, 10 vol% THF). The resulting solution was thoroughly mixed by drawing up the entire volume into the pipette tip and ejecting the liquid back into the vial three times. The resulting solution was analyzed by DLS and TEM.

DLS

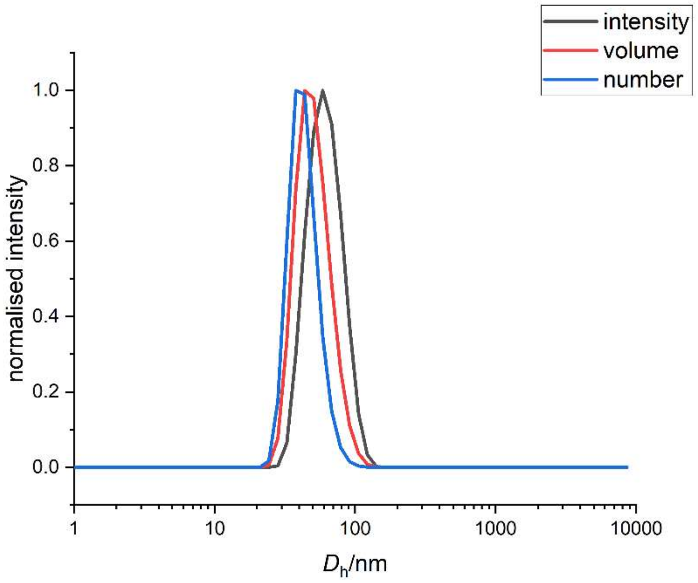

Figure S26 DLS traces of $\mathbf{P3_{200}}$ particles as synthesized after attempted triggered fusion.

.

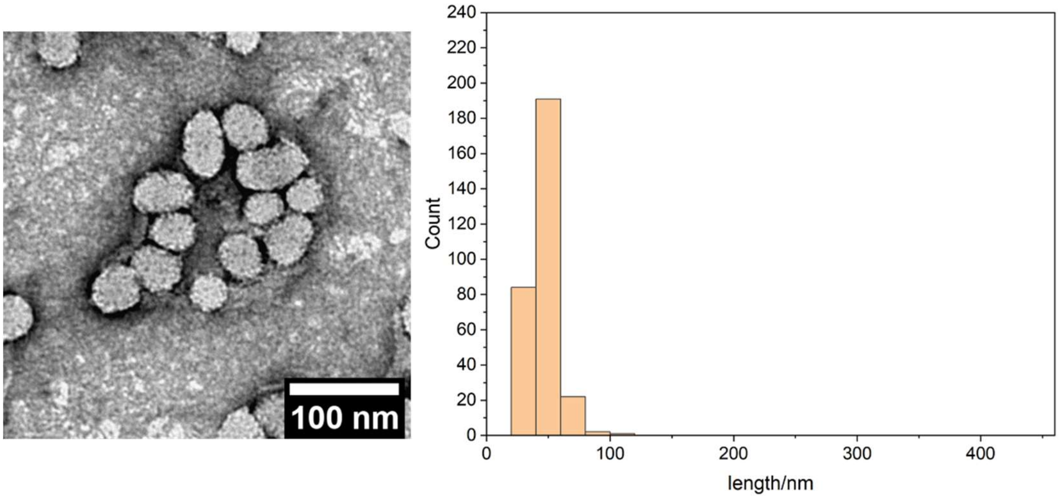

Figure S27 Dry-state TEM image and histogram (300 particles analyzed) of **P3$_{200}$** after attempted triggered fusion.

8. Interrupted fusion experiments

Fusion of **P1$_{200}$** particles was triggered as detailed in section 6. 40 µL aliquots were taken at five seconds and five minutes and immediately quenched in 960 µL PB2 solution (i.e. diluted to the concentration used for DLS analysis and TEM grid preparation) and analyzed.

Characterization Summary

nanoparticle	$Z_{avg,DLS}$ /nm	PD_{DLS}	L_{TEM} /nm
**P1$_{200}$** @ 5 seconds	172	0.25	84±55
**P1$_{300}$** @ 5 minutes	177	0.23	87±59

DLS

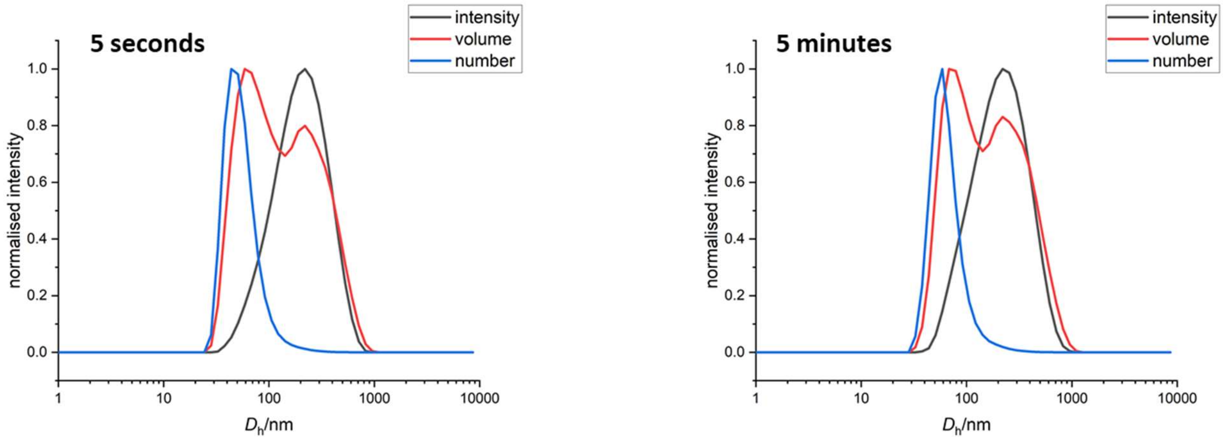

Figure S28 DLS plot of **P1$_{200}$** particles quenched five seconds and five minutes after pH trigger.

Dry-state TEM

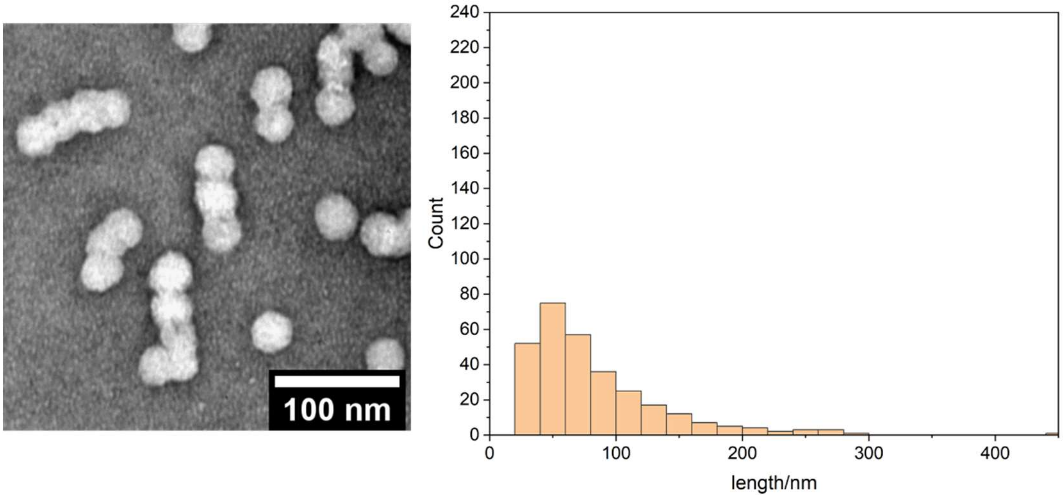

Figure S29 Dry-state TEM image and histogram (300 particles analyzed) of **P1$_{200}$** quenched after five seconds of fusion.

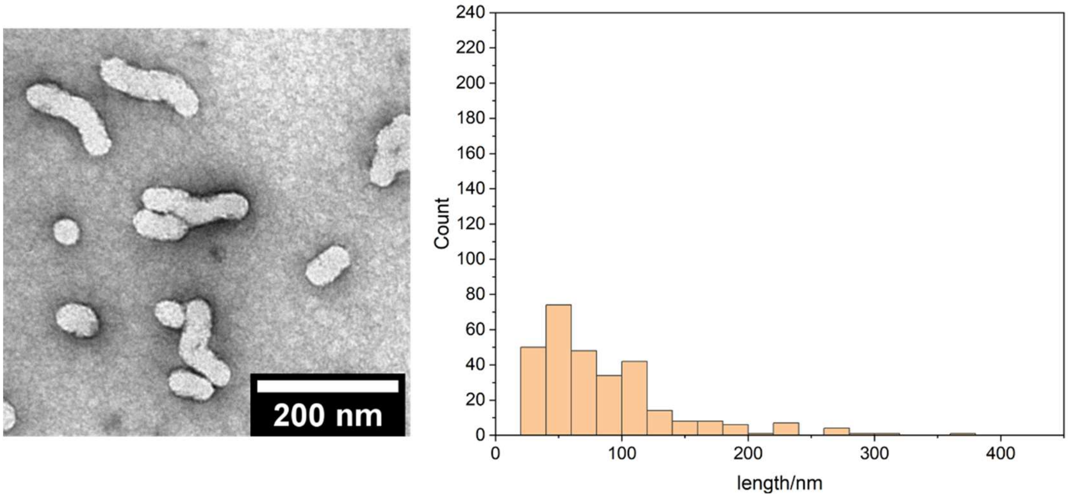

Figure S30 Dry-state TEM image and histogram (300 particles analyzed) of **P1$_{200}$** quenched after five minutes of fusion.

9. Synthesis and characterization of P(**NB-PEG**)$_{11}$-*b*-P(**NB-amine**)$_{2.5}$-*b*-P(**NB-py**)$_{2.5}$-*b*-P(**NB-MEG**)$_{200}$, P4$_{200}$

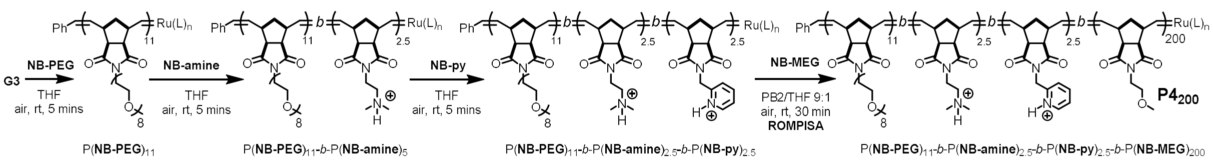

Scheme S5 Synthesis of **P4$_{200}$**.

A solution of **NB-PEG** (31.2 mg, 59 µmol, 13 eq.) in 530 µL of filtered THF was rapidly added to a solution of **G3** (3.8 mg, 5.2 µmol, 1.2 eq.) in 120 µL of THF contained within a glass vial equipped with a stirrer bar. The resulting solution was stirred rapidly for five minutes to give P(**NB-PEG**)$_{11}$. After this time a 100 µL aliquot was removed for GPC analysis, leaving 1.0 eq. of P(**NB-PEG**)$_{11}$ in 550 µL of THF. To this solution was added **NB-amine** (2.6 mg, 11 µmol, 2.5 eq.) in 450 µL THF. The resulting solution was stirred for a further five minutes to give P(**NB-PEG**)$_{11}$-*b*-P(**NB-amine**)$_{2.5}$. After this time a 100 µL aliquot was removed for GPC analysis, leaving 0.9 eq. of P(**NB-PEG**)$_{11}$-*b*-P(**NB-amine**)$_{2.5}$ in 900 µL of THF. To this solution was added **NB-py** (2.5 mg, 11 µmol, 2.3 eq.) in 450 µL THF. The resulting solution was stirred for a further five minutes to give P(**NB-PEG**)$_{11}$-*b*-P(**NB-amine**)$_{2.5}$-*b*-P(**NB-Py**)$_{2.5}$ macroinitiator (final concentration = 3.3 µmol/mL).

An aliquot (68 µL, 0.22 µmol) of the resulting solution of (**NB-PEG**)$_{11}$-*b*-P(**NB-Amine**)$_{2.5}$-*b*-P(**NB-py**)$_{2.5}$ in THF was dispensed into a 2 mL glass vial containing a stirrer bar. Filtered THF and PB2 was added to give 100 µL in total of each. A solution of **NB-MEG** (10 mg, 45 µmol) in 0.8 mL of acidic phosphate buffer (pH = 2, PB2, final solids concentration = 1 wt%) was added rapidly. The resulting solution was thoroughly mixed by drawing up the entire volume into the pipette tip and ejecting the liquid back into the vial. The ROMPISA polymerizations were stirred at 300 rpm for 30 minutes to give P(**NB-PEG**)$_{11}$-*b*-P(**NB-amine**)$_{2.5}$-*b*-P(**NB-py**)$_{2.5}$ *b*-P(**NB-MEG**)$_{200}$ (**P4$_{200}$**). This was analyzed by ^{1}H NMR, GPC, DLS and TEM.

Characterization Summary

Polymer	$M_{n,theo}$ /kDa	$M_{n,GPC}$ /kDa	$Đ_{GPC}$	$Z_{avg,DLS}$ /nm	PD_{DLS}	L_{TEM} /nm
P(**NB-PEG**)$_{11}$	6.6	6.9	1.13	n/a	n/a	n/a
P(**NB-PEG**)$_{11}$-*b*-P(**NB-amine**)$_{2.5}$	7.2	7.6	1.13	n/a	n/a	n/a
P(**NB-PEG**)$_{11}$-*b*-P(**NB-amine**)$_{2.5}$-*b*-P(**NB-py**)$_{2.5}$	7.8	8.5	1.11	n/a	n/a	n/a
**P4$_{200}$** @ pH 2	52	52	1.11	53	0.07	43±10

^{1}H NMR

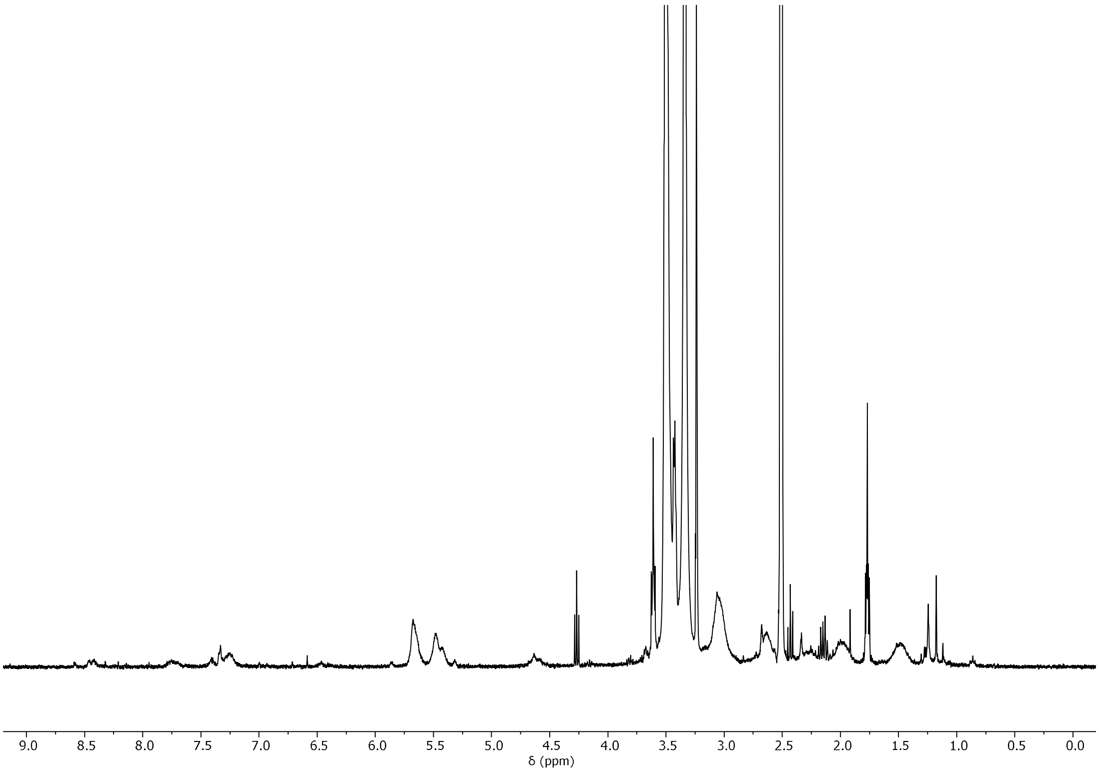

Spectrum S17 ^{1}H NMR (300 MHz, $(CD_3)_2SO$, 300 K) of P(**NB-PEG**)$_{11}$-*b*-P(**NB-amine**)$_{2.5}$-*b*-P(**NB-py**)$_{2.5}$ + THF

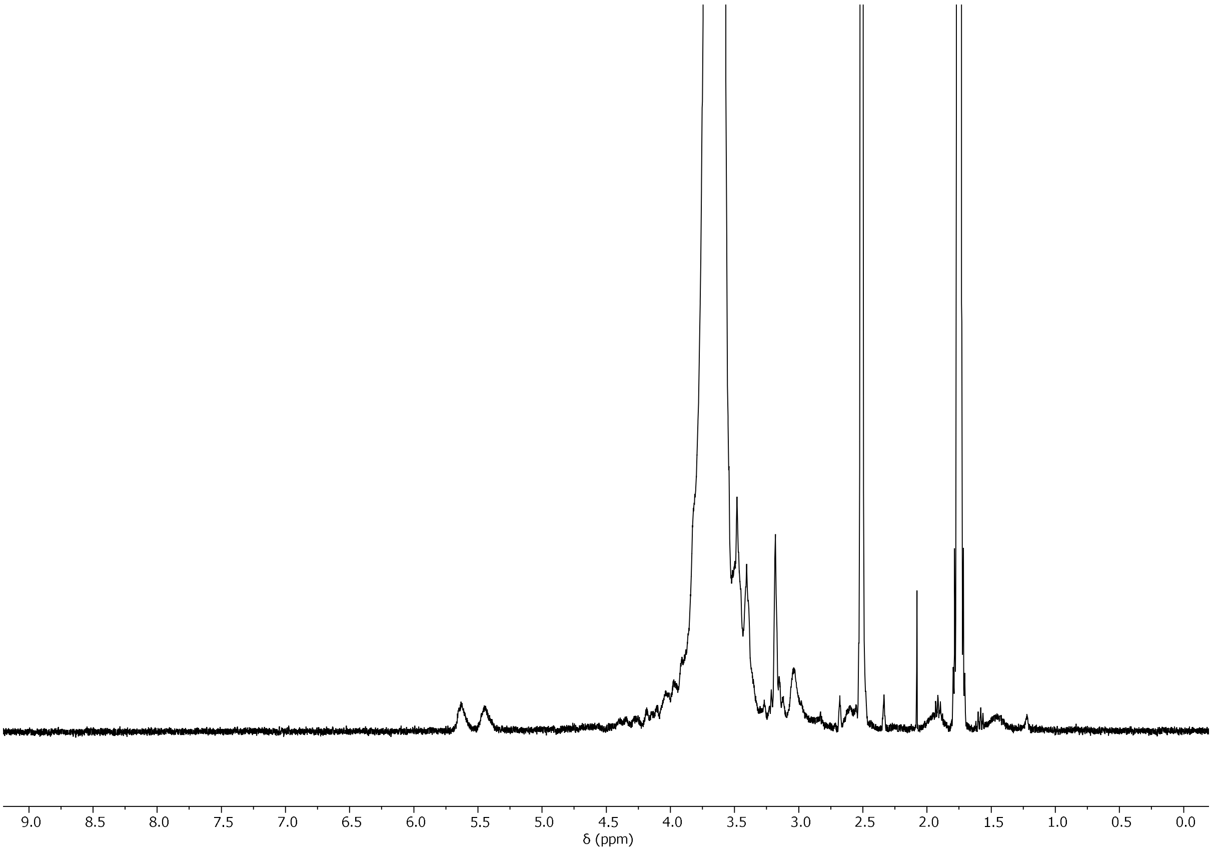

Spectrum S18 ^{1}H NMR (300 MHz, $(CD_3)_2SO$, 300 K) of **P4$_{200}$** + THF/PB2

GPC

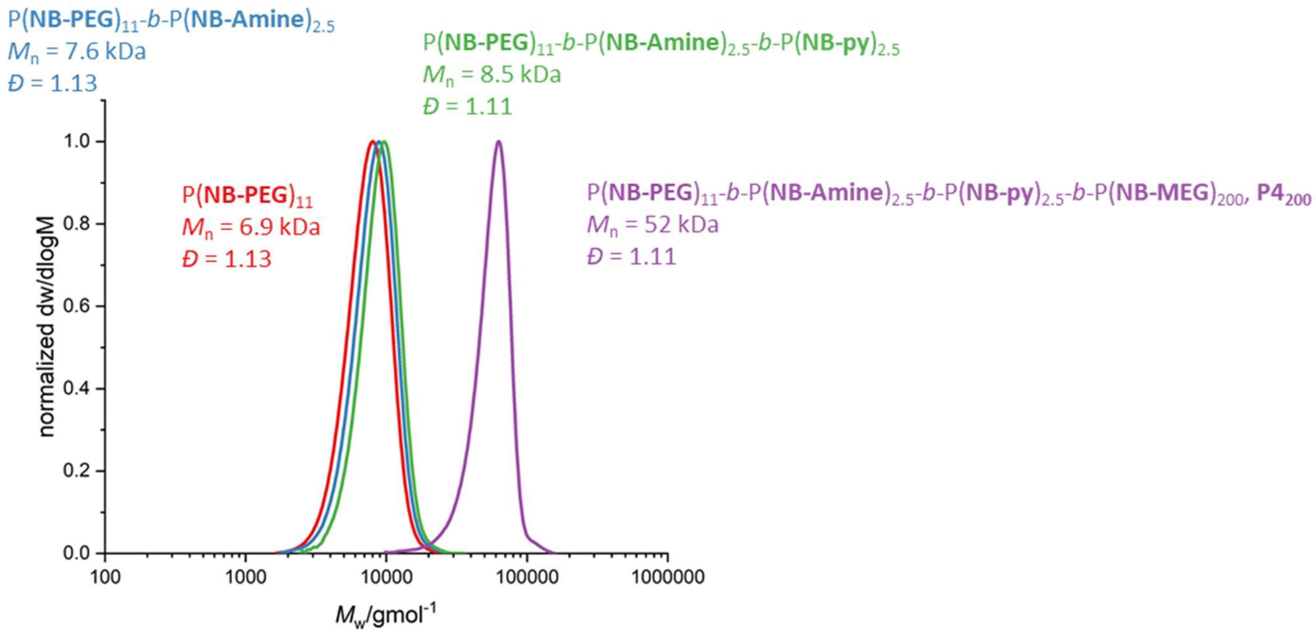

Figure S31 Normalized GPC trace (THF eluent, PMMA standards) of **$P4_{200}$** and intermediate polymers.

DLS

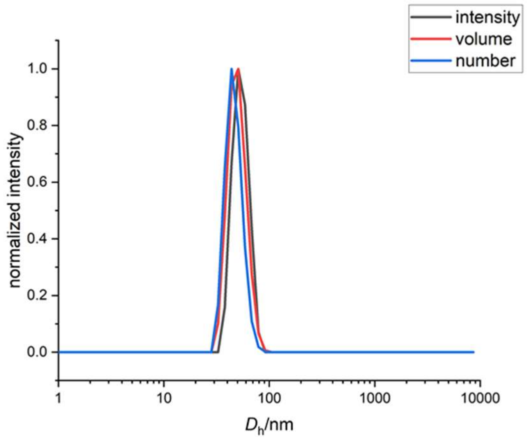

Figure S32 DLS traces of **$P4_{200}$** particles as synthesized at pH 2.

Dry State TEM

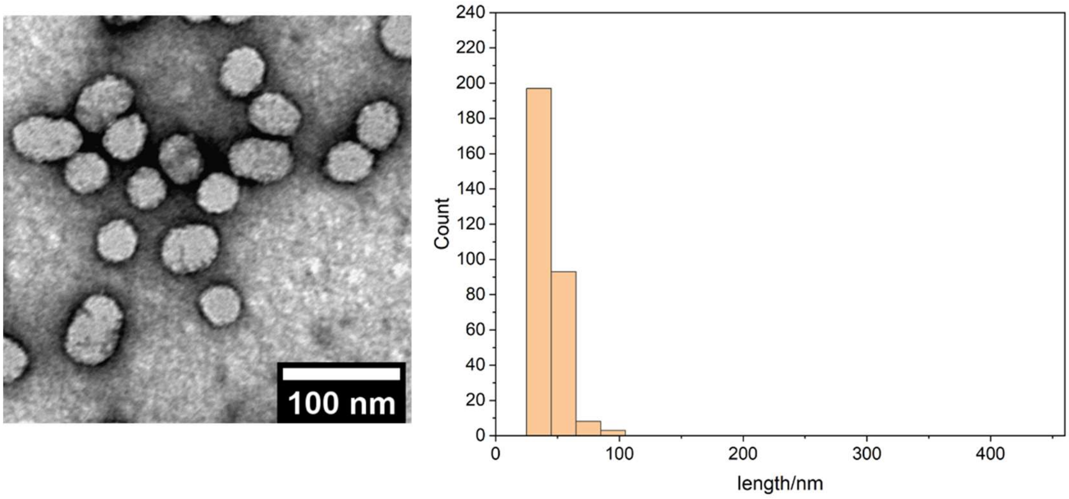

Figure S33 Dry-state TEM image and histogram (300 particles analyzed) of $\mathbf{P4_{200}}$ particles as synthesized at pH 2.

Triggered fusion of P(NB-PEG)$_{11}$-*b*-P(NB-amine)$_{2.5}$-*b*-P(NB-py)$_{2.5}$-*b*-P(NB-MEG)$_{200}$, P4$_{200}$

To 100 µL solution of **P4$_{200}$** (as synthesized, 10 vol% THF in PB2) was rapidly added 100 µL of NaOH solution (100 mM, 10 vol% THF in deionised water) to give a pH 7 solution. The resulting solution was thoroughly mixed by drawing up the entire volume into the pipette tip and ejecting the liquid back into the vial three times. A 20 µL aliquot was taken for analysis by TEM and DLS. A further 200 µL of NaOH solution was added to adjust the remaining solution to pH 12. The resulting solution was analyzed by DLS and TEM.

Characterization Summary

nanoparticle	$Z_{avg,DLS}$ /nm	PD_{DLS}	L_{TEM} /nm
**P4$_{200}$** @ pH 7	62	0.10	55±19
**P1$_{300}$** @ pH 12	117	0.15	127±83

DLS

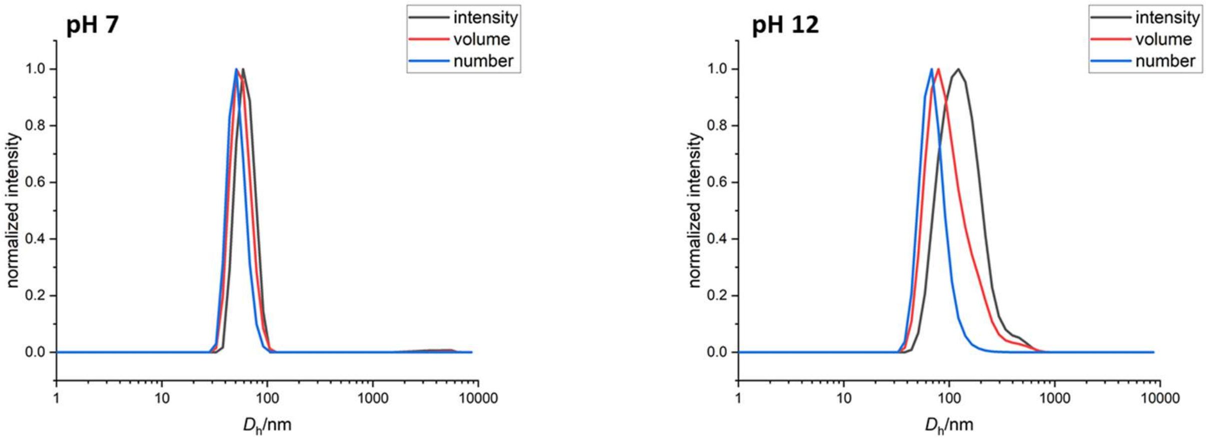

Figure S34 DLS traces of **P4$_{200}$** particles after triggered fusion to pH 7 and pH 12.

Dry-state TEM

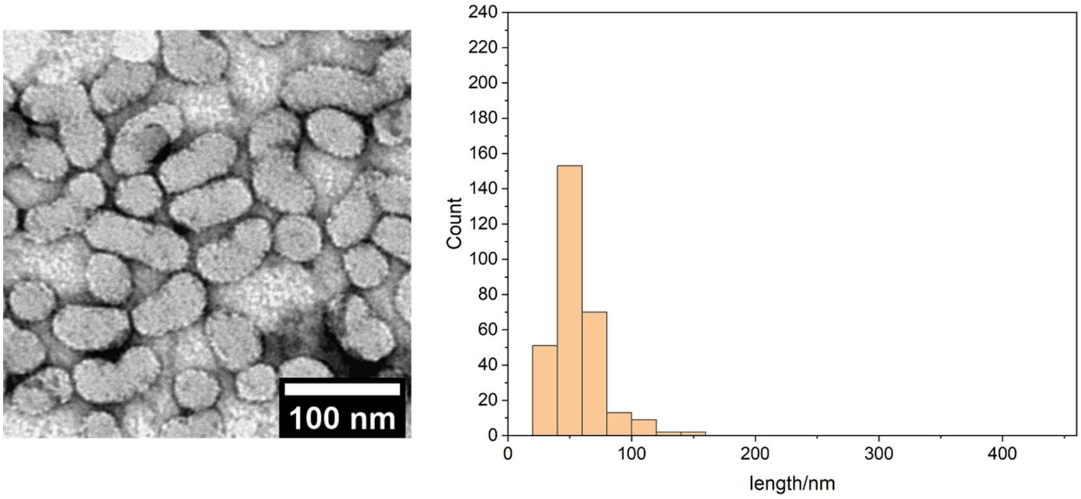

Figure S35 Dry-state TEM image and histogram (300 particles analysed) of **P4$_{200}$** particles after pH 7 trigger.

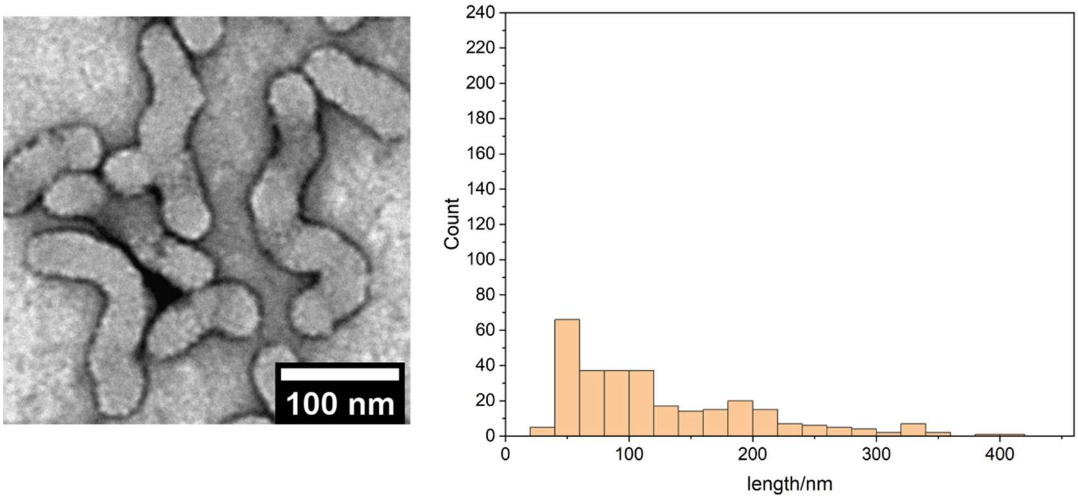

Figure S36 Dry-state TEM image and histogram (300 particles analysed) of **P4$_{200}$** particles after pH 12 trigger.

10. SAXS analysis of the fusion of $P1_{200}$ particles

10.1 SAXS Modelling

Programming tools within the Irena SAS macros for Igor Pro were used to model experimental SAXS data.[7] Models used to fit SAXS data in this work were previously developed by Pedersen *et al.*[8]

In general, the intensity of X-rays scattered by a dispersion of nano-objects [as represented by the scattering cross-section per unit sample volume, $\frac{d\Sigma}{d\Omega}(q)$] can be expressed as:

$$\frac{d\Sigma}{d\Omega}(q) = NS(q)\int_0^\infty \dots \int_0^\infty F(q, r_1, \dots, r_k)^2 \Psi(r_1, \dots, r_k) dr_1, \dots, dr_k \qquad \text{(S1)}$$

where $F(q, r_1, \dots, r_k)$ is the form factor, $r_1, \dots, r_k$ is a set of *k* parameters describing the nano-object structural morphology, $\Psi(r_1, \dots, r_k)$ is the distribution function, *S*(*q*) is the structure factor and *N* is the number density of nano-objects per unit volume expressed as:

$$N = \frac{\varphi}{\int_0^\infty \dots \int_0^\infty V(r_1, \dots, r_k)\Psi(r_1, \dots, r_k) dr_1, \dots, dr_k} \qquad \text{(S2)}$$

where $V(r_1, \dots, r_k)$ is the nano-object volume and φ is the volume fraction of the nano-objects within the dispersion. It is assumed that *S*(*q*) = 1 at the sufficiently low copolymer concentrations used in this study (≤1.0% w/w).

For this study, experimental SAXS data required fitting to either the spherical micelle model (e.g. static data obtained at pH2), cylindrical micelle (e.g. static data obtained at pH12), or a combination of the two (e.g. all time-resolved data). Additionally, optimum fits often required the use a power law relationship (herein described as a unified fit) and/or a low-intensity background to adequately fit low-*q* and high-*q* data, respectively. Thus, the intensity of scattering at a given *q* vector, $I(q)$, is expressed as:

$$I(q) = \frac{d\Sigma}{d\Omega}(q)_{\text{sphere}} + \frac{d\Sigma}{d\Omega}(q)_{\text{cylinder}} + Bq^{-P} + background \qquad \text{(S3)}$$

where $\frac{d\Sigma}{d\Omega}(q)_{\text{sphere}}$ is the form factor for spherical micelles, $\frac{d\Sigma}{d\Omega}(q)_{\text{cylinder}}$ is the form factor for cylindrical micelles, and terms B, P and $background$ are constants.

Spherical micelle model

The spherical micelle form factor for Equation S1, which contributes to $\frac{d\Sigma}{d\Omega}(q)_{\text{sphere}}$, is given by:

$$F_{\text{s}_{\text{mic}}}(q) = N_{\text{s}}^2\beta_{\text{s}}^2 A_{\text{s}}^2(q, R_{\text{s}}) + N_{\text{s}}\beta_{\text{c}}^2 F_{\text{c}}(q, R_{\text{g}}) + N_{\text{s}}(N_{\text{s}} - 1)\beta_{\text{c}}^2 A_{\text{c}}^2(q) + 2N_{\text{s}}^2\beta_{\text{s}}\beta_{\text{c}} A_{\text{s}}(q, R_{\text{s}}) A_{\text{c}}(q) \quad \text{(S4)}$$

where R_{s} is the volume-average sphere core radius and R_{g} is the radius of gyration of the coronal steric stabilizer block (in this case, P(**NB-PEG**), which was fixed at 32.6 Å for data fits). The X-ray scattering length contrasts for the core and corona blocks are given by $\beta_{\text{s}} = V_{\text{s}}(\xi_{\text{s}} - \xi_{\text{sol}})$ and $\beta_{\text{c}} = V_{\text{c}}(\xi_{\text{c}} - \xi_{\text{sol}})$ respectively. Here, ξ_{s}, ξ_{c} and ξ_{sol} are the X-ray scattering length densities of the core block ($\xi_{\text{P}(\mathbf{NB-MEG})}$= 10.77 x 10^{10} cm^{-2}), corona block ($\xi_{\text{P(NB-PEG)}}$= 10.31 x 10^{10} cm^{-2}) and water (ξ_{sol}= 9.42 x 10^{10} cm^{-2}), respectively. V_{s} and V_{c} are the volumes of the core block ($V_{\text{P(NB-MEG)}}$ = 61750 $Å^3$) and the corona block ($V_{\text{P(NB-PEG)}}$ = 8600 $Å^3$), respectively. Values for V_{s} and V_{c} were calculated using $V = \frac{M_{\text{n}_{\text{pol}}}}{N_{\text{A}}\rho}$ taking the solid-state homopolymer densities of P(**NB-MEG**) determined by helium pycnometry ($\rho_{\text{P}(\mathbf{NB-MEG})}$ = 1.19 g cm^{-3}) and P(**NB-PEG**), which was taken to equal that of PEG ($\rho_{\text{P}(\mathbf{NB-PEG})}$ = 1.13 g cm^{-3}),[9] where $M_{\text{n}_{\text{pol}}}$ is the number-average molecular weight of each polymer block determined by ^{1}H NMR spectroscopy. The sphere form factor amplitude is used for the amplitude of the core self-term:

$$A_{\text{c}}(q, R_{\text{s}}) = \Phi(qR_{\text{s}})\exp\left(-\frac{q^2\sigma^2}{2}\right) \quad \text{(S5)}$$

where $\Phi(qR_{\text{s}}) = \frac{3[\sin(qR_{\text{s}}) - qR_{\text{s}}\cos(qR_{\text{s}})]}{(qR_{\text{s}})^3}$. A sigmoidal interface between the two blocks was assumed for the spherical micelle form factor (Equation S4). This is described by the exponent term with a width σ accounting for a decaying scattering length density at the micellar interface. This σ value was fixed at 2.2 during fitting.

The form factor amplitude of the spherical micelle corona is:

$$A_{\text{c}}(q) = \frac{\int_{R_{\text{s}}}^{R_{\text{s}}+2s} \mu_c(r)\frac{\sin(qr)}{qr} r^2 dr}{\int_{R_{\text{s}}}^{R_{\text{s}}+2s} \mu_c(r) r^2 dr} exp\left(-\frac{q^2\sigma^2}{2}\right) \quad \text{(S6)}$$

The radial profile, $\mu_c(r)$, can be expressed by a linear combination of two cubic b splines, with two fitting parameters s and a corresponding to the width of the profile and the weight coefficient respectively. This information can be found elsewhere,[10,11] as can the approximate integrated form of Equation S5. The self-correlation term for the coronal block is given by the Debye function:

$$F_{\mathrm{c}}(q,R_{\mathrm{g}}) = \frac{2\left[\exp(-q^2 {R_{\mathrm{g}}}^2) - 1 + q^2 {R_{\mathrm{g}}}^2\right]}{q^4 {R_{\mathrm{g}}}^4} \tag{S7}$$

where R_{g} is the radius of gyration of the P(NB-PEG) coronal block. The aggregation number, N_s, of the spherical micelle is given by:

$$N_{\mathrm{s}} = (1 - x_{\mathrm{sol}}) \frac{\frac{4}{3}\pi {R_{\mathrm{s}}}^3}{V_{\mathrm{s}}} \tag{S8}$$

where x_{sol} is the volume fraction of solvent within the P(NB-MEG) micelle cores, which was found to be zero in all cases. A polydispersity for one parameter (R_{s}) is assumed for the micelle model, which is described by a Gaussian distribution. Thus, the polydispersity function in Equation S1 can be represented as:

$$\Psi(r_1) = \frac{1}{\sqrt{2\pi {\sigma_{R_{\mathrm{s}}}}^2}} exp\left(-\frac{(r_1 - R_{\mathrm{s}})^2}{2{\sigma_{R_{\mathrm{s}}}}^2}\right) \tag{S9}$$

where $\sigma_{R_{\mathrm{s}}}$ is the standard deviation for R_{s}. In accordance with Equation S2, the number density per unit volume for the micelle model is expressed as:

$$N = \frac{\varphi_{\mathrm{s}}}{\int_0^\infty V(r_1)\Psi(r_1)dr_1} \tag{S10}$$

where φ_{s} is the total volume fraction of copolymer in the spherical micelles and $V(r_1)$ is the total volume of copolymer within a spherical micelle $[V(r_1) = (V_{\mathrm{s}} + V_{\mathrm{c}})N_{\mathrm{s}}(r_1)]$.

Cylindrical micelle model

The cylindrical micelle form factor for Equation S1 is given by:

$$F_{\mathrm{cyl}}(q) = {N_{\mathrm{cyl}}}^2 {\beta_{\mathrm{s}}}^2 F_{\mathrm{sw}}(q) + N_{\mathrm{w}} {\beta_{\mathrm{c}}}^2 F_{\mathrm{c}}(q,R_{\mathrm{g}}) + N_{\mathrm{cyl}}(N_{\mathrm{cyl}} - 1){\beta_{\mathrm{c}}}^2 S_{\mathrm{cc}}(q) + 2{N_{\mathrm{cyl}}}^2 \beta_{\mathrm{s}}\beta_{\mathrm{c}} S_{\mathrm{sc}}(q) \tag{S11}$$

where all the parameters are the same as those described in the spherical micelle model (Equation S4), unless stated otherwise.

The self-correlation term for the cylinder core cross-sectional volume-average radius R_{cyl} is:

$$F_{\mathrm{cyl}}(q) = F_{\mathrm{cyl}}(q, L_{\mathrm{cyl}}, b_{\mathrm{cyl}}) {A_{\mathrm{CS}_{\mathrm{cyl}}}}^{2}(q, R_{\mathrm{cyl}}) \qquad \text{(S12)}$$

where

$${A_{\mathrm{CS}_{\mathrm{cyl}}}}^{2}(q, R_{\mathrm{cyl}}) = \left[2\frac{J_1(qR_{\mathrm{cyl}})}{qR_{\mathrm{cyl}}}\right]^2 \qquad \text{(S13)}$$

and J_1 is the first-order Bessel function of the first kind, and a form factor $F_{\mathrm{cyl}}(q, L_{\mathrm{cyl}}, b_{\mathrm{cyl}})$ for self-avoiding semi-flexible chains represents the cylindrical micelles, where b_{cyl} is the Kuhn length and L_{cyl} is the mean contour length. In all cases when applying the cylindrical micelle model, b_{cyl} was found to equal the value of L_{cyl}, indicating the presence of rigid cylinders. A complete expression for the chain form factor can be found elsewhere.[12]

The mean aggregation number of the cylindrical micelle, N_{cyl}, is given by:

$$N_{\mathrm{cyl}} = (1 - x_{\mathrm{sol}})\frac{\pi {R_{\mathrm{cyl}}}^{2} L_{\mathrm{cyl}}}{V_{\mathrm{s}}} \qquad \text{(S14)}$$

Again, x_{sol} was found to be zero in all cases. The possible presence of semi-spherical caps at both ends of each worm is neglected in this form factor.

A polydispersity for one parameter (R_{cyl}) is assumed for the cylindrical micelle model, which is described by a Gaussian distribution. Thus, the polydispersity function in Equation S1 can be represented as:

$$\Psi(r_1) = \frac{1}{\sqrt{2\pi {\sigma_{R_{\mathrm{cyl}}}}^{2}}} exp\left(-\frac{(r_1 - R_{\mathrm{cyl}})^2}{2{\sigma_{R_{\mathrm{cyl}}}}^{2}}\right) \qquad \text{(S15)}$$

where $\sigma_{R_{\mathrm{cyl}}}$ is the standard deviation for R_{cyl}. In accordance with Equation S2, the number density per unit volume for the worm-like micelle model is expressed as:

$$N = \frac{\varphi_{\mathrm{cyl}}}{\int_0^{\infty} V(r_1)\Psi(r_1)dr_1} \qquad \text{(S16)}$$

where φ_{cyl} is the total volume fraction of copolymer in the cylindrical micelles and $V(r_1)$ is the total volume of copolymer in a cylindrical micelle $[V(r_1) = (V_{\mathrm{s}} + V_{\mathrm{c}})N_{\mathrm{cyl}}(r_1)]$.

Table S1. Summary of variable fitting parameters for all SAXS data analyzed in this work.

	Sample	Spherical micelle model			Cylindrical micelle model				Power law		$background$
		φ_s	R_s (Å)	σ_{R_s} (Å)	φ_{cyl}	R_{cyl} (Å)	$\sigma_{R_{cyl}}$ (Å)	L_{cyl} (Å)	B	P	
**Static**	pH2	0.0200	165	16.9	0	0	0	0	0.0342	1.04	0
	pH12	0	0	0	0.00679	168	24.8	1320	0.0000367	2.52	0
**Time-resolved**	1 s	0.0112	165	16.9	0.00445	139	20.3	474	0.00184	1.74	0.0171
	2 s	0.00815	165	16.9	0.00556	142	22.8	735	0.426	0.992	0.0113
	3 s	0.00820	165	16.9	0.00589	148	25.7	758	1.07	0.913	0.0128
	4 s	0.00770	165	16.9	0.00589	150	22.1	807	0.120	1.27	0.0103
	5 s	0.00660	165	16.9	0.00655	155	21.8	857	0.0125	1.67	0.0124
	10 s	0.00621	165	16.9	0.00680	157	25.4	1130	0.000661	2.16	0.0133
	30 s	0.00547	165	16.9	0.00669	163	20.4	1220	0.000197	2.37	0.0103
	60 s	0.00548	165	16.9	0.00714	165	19.4	1260	0.000152	2.40	0.0116
	120 s	0.00498	165	16.9	0.00722	168	15.7	1300	0.0000716	2.53	0.00921
	180 s	0.00471	165	16.9	0.00729	168	16.8	1320	0.0000314	2.70	0.00806
	240 s	0.00469	165	16.9	0.00810	169	19.6	1320	0.0000217	2.75	0.00879
	300 s	0.00474	165	16.9	0.00835	169	17.2	1330	0.0000125	2.86	0.00732

Table S2. Summary of values calculated using SAXS fitting parameters outlined in Table S1.

	Sample	Mean sphere diameter, $\boldsymbol{D_s}$ (nm) $D_s = 2R_s + 4R_g$	Mean sphere aggregation number, $\boldsymbol{N_s}$ $N_s = \frac{\frac{4}{3}\pi {R_s}^3}{V_s}$	Mean cylinder cross-sectional diameter, $\boldsymbol{D_{cyl}}$ (nm) $D_{cyl} = 2R_{cyl} + 4R_g$	Mean cylinder length, $\boldsymbol{L_{cyl}}$ (nm)	Mean cylinder aggregation number, $\boldsymbol{N_{cyl}}$ $N_{cyl} = \frac{\pi {R_{cyl}}^2 L_{cyl}}{V_s}$	Mean cylinder aspect ratio $\frac{L_{cyl}}{D_{cyl}}$	Mean number of spheres fused to form a cylinder $\frac{N_{cyl}}{N_s}$	Volume fraction of cylinders present $\frac{\varphi_{cyl}}{\varphi_{cyl} + \varphi_s}$
**Static**	pH2	46.1	306	-	-	-	-	-	0
	pH12	-	-	46.6	132	1900	2.84	6.19	1
**Time-resolved**	1 s	46.1	306	40.9	47.4	468	1.16	1.53	0.284
	2 s	46.1	306	41.5	73.5	757	1.77	2.47	0.406
	3 s	46.1	306	42.6	75.8	845	1.78	2.76	0.418
	4 s	46.1	306	42.9	80.7	918	1.88	3.00	0.434
	5 s	46.1	306	44.0	85.7	1050	1.95	3.42	0.498
	10 s	46.1	306	44.4	113	1420	2.55	4.64	0.523
	30 s	46.1	306	45.6	122	1640	2.68	5.37	0.550
	60 s	46.1	306	46.0	126	1730	2.73	5.66	0.566
	120 s	46.1	306	46.6	130	1860	2.79	6.09	0.592
	180 s	46.1	306	46.6	132	1890	2.82	6.17	0.608
	240 s	46.1	306	46.8	132	1920	2.82	6.26	0.633
	300 s	46.1	306	46.8	133	1940	2.85	6.34	0.638

10.2 Comparison of static and *in situ* SAXS analysis

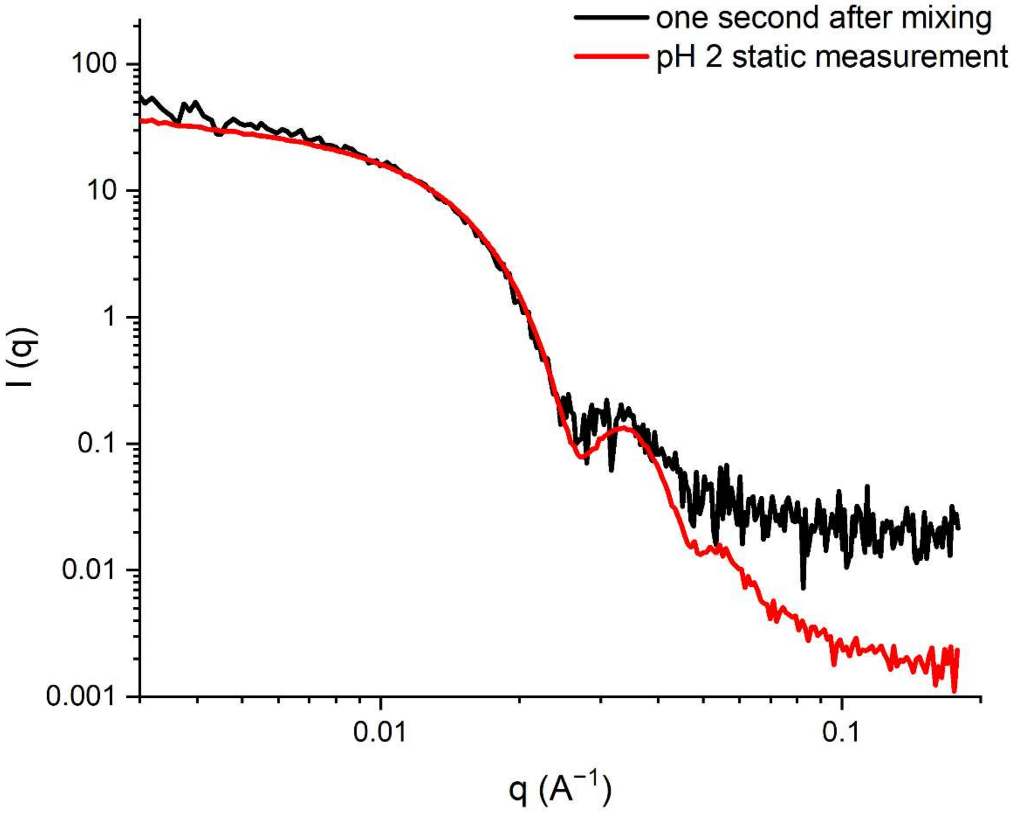

Figure S37 SAXS data of **P1$_{200}$** particles as synthesized at pH 2 (red line) and one second after mixing in stopped-flow capillary (black line).

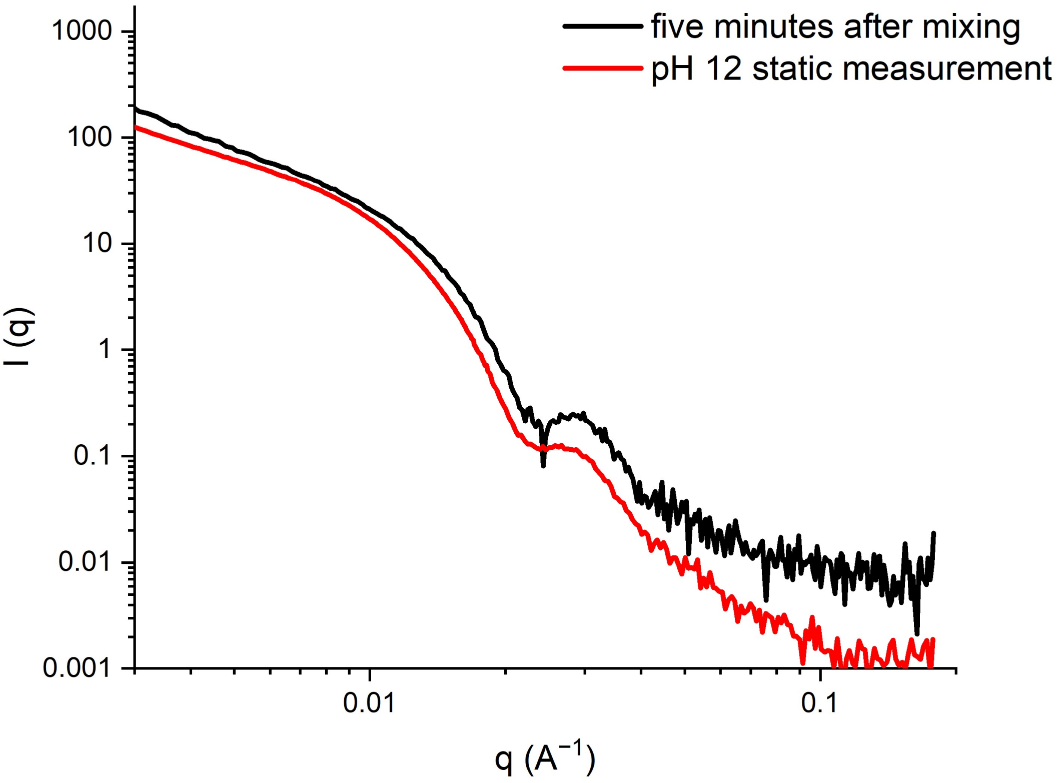

Figure S38 SAXS data of fused **P1**$_{200}$ particles at pH 12 produced by a conventional laboratory experiment (red line) and five minutes after mixing in stopped-flow capillary (black line).

10.3 Representative example of modelled *in situ* SAXS data

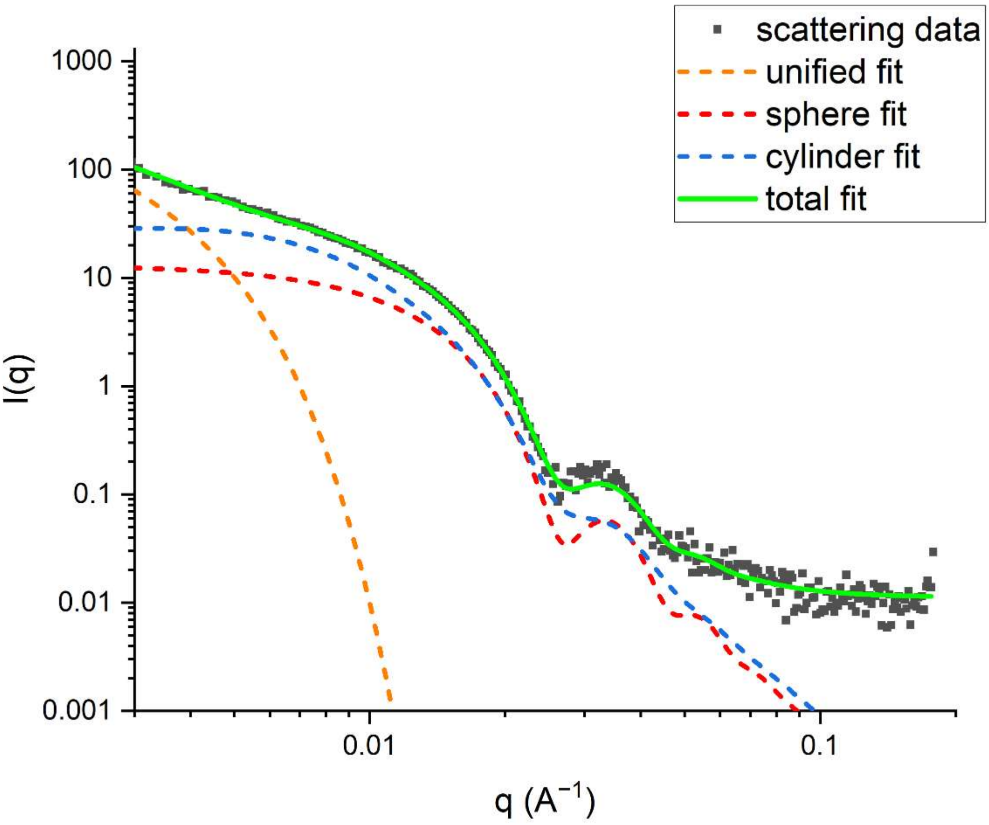

Figure S39 SAXS data of **P1$_{200}$** particles two seconds after mixing in stopped-flow capillary (black scatter). Total fit (green line) obtained from a combination of a spherical micelle model (dashed red line),[8] a cylindrical micelle model (dashed blue line)[8] and a unified fit (orange line).

10.4 Synthesis of P(NB-MEG)$_{200}$

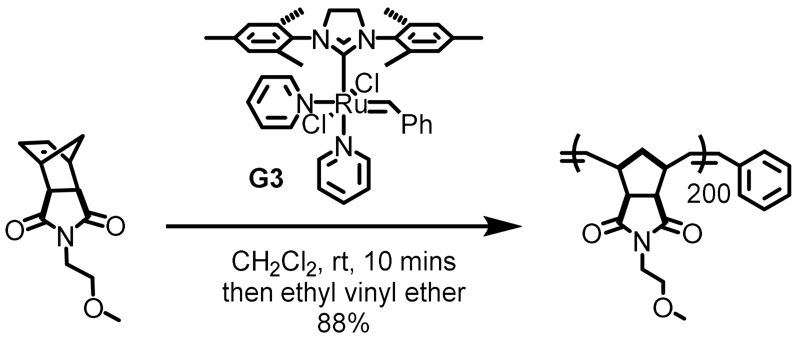

Scheme 6 Synthesis of P(**NB-MEG**)$_{200}$

A solution of **G3** (8.2 mg, 11 µmol, 1.0 eq.) in CH_2Cl_2 (1 mL) was rapidly added to a stirring solution of **NB-MEG** (496 mg, 2.2 mmol, 200 eq.) in CH_2Cl_2 (2 mL). The resulting solution was rapidly stirred for 10 minutes before ethyl vinyl ether (1 mL) was added. The solution was then diluted with ice cold Et_2O (200 mL) to induce precipitation. The precipitate was filtered, washed with Et_2O (100 mL) and dried under reduced pressure to afford P(**NB-MEG**)$_{200}$ (438 mg, 88%) as a colourless solid.

Characterization Summary

Polymer	$M_{n,theo}$/kDa	$M_{n,GPC}$/kDa	$Đ_{GPC}$
P(**NB-MEG**)$_{200}$	44	39	1.09

NMR

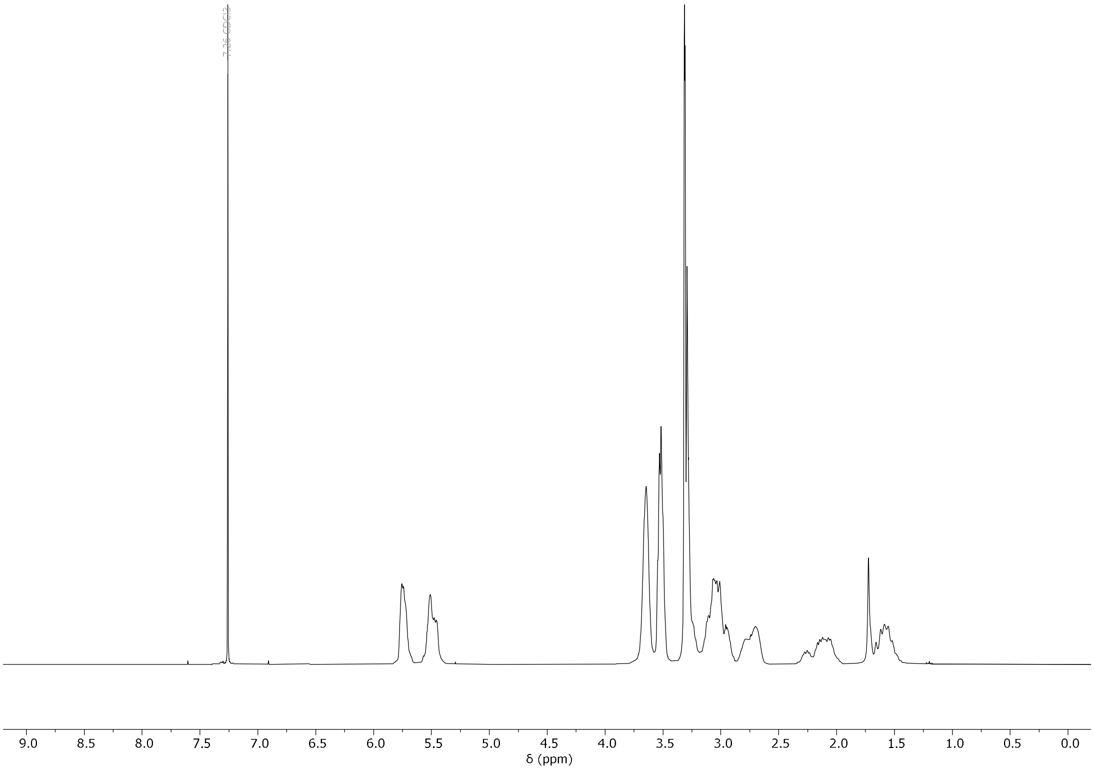

Spectrum S19 ^{1}H NMR (300 MHz, $CDCl_3$, 298 K) of P(**NB-MEG**)$_{200}$

GPC

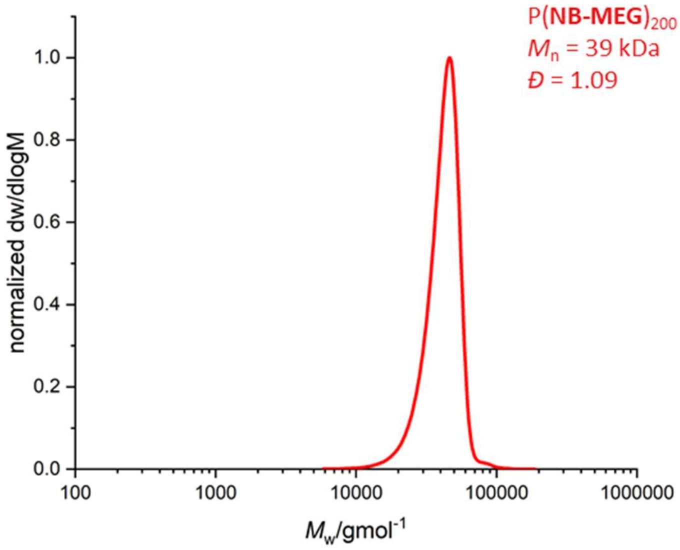

Figure S40 Normalized GPC trace (THF eluent, PMMA standards) of P(**NB-MEG**)$_{200}$

11. References

1. Radzinski, S. C.; Foster, J. C.; Scannelli, S. J.; Weaver, J. R.; Arrington, K. J.; Matson, J. B. Tapered Bottlebrush Polymers: Cone-Shaped Nanostructures by Sequential Addition of Macromonomers*. ACS Macro Lett.* **2017**, *6*, 1175–1179.
2. Varlas, S.; Foster, J. C.; Arkinstall, L. A.; Jones, J. R.; Keogh, R.; Mathers, R. T.; O'Reilly, R. K. Predicting Monomers for Use in Aqueous Ring-Opening Metathesis Polymerization-Induced Self-Assembly. *ACS Macro Lett.* **2019**, *8*, 466–472.
3. Filik, J.; Ashton, A. W.; Chang, P. C. Y.; Chater, P. A.; Day, S. J.; Drakopoulos, M.; Gerring, M. W.; Hart, M. L.; Magdysyuk, O. V.; Michalik, S.; Smith, A.; Tang, C. C.; Terrill, N. J.; Wharmby, M. T.; Wilhelm, H. Processing two-dimensional X-ray diffraction and small-angle scattering data in DAWN 2. *J. Appl. Crystallogr.* **2017**, *50*, 959–966.
4. J. Ilavsky and P. R. Jemian. Irena: tool suite for modeling and analysis of small-angle scattering. *J. Appl. Crystallogr.* **2009**, *42*, 347–353.
5. *Materials Studio 2020* Accelrys Software Inc.: San Diego, 2020
6. Ghose, A. K.; Viswanadhan, V. N.; Wendoloski, J. J. Prediction of Hydrophobic (Lipophilic) Properties of Small Organic Molecules Using Fragmental Methods: An Analysis of ALOGP and CLOGP Methods. *J. Phys. Chem. A* **1998**, *102*, 3762–3772.
7. Sun, H.; Jin, Z.; Yang, C. W.; Akkermans, R. L. C.; Robertson, S. H.; Spenley, N. A.; Miller, S.; Todd, S. M. COMPASS II: extended coverage for polymer and drug-like molecule databases. *J. Mol. Model.* **2016**, *22*, 47.
8. Pedersen, J. S. Form factors of block copolymer micelles with spherical, ellipsoidal and cylindrical cores. *J. Appl. Crystallogr.* **2000**, *33*, 637–640.
9. Sponchioni, M.; O'Brien, C. T.; Borchers, C.; Wang, E.; Rivolta, M. N.; Penfold, N. J. W.; Canton, I.; Armes, S. P. Probing the mechanism for hydrogel-based stasis induction in human pluripotent stem cells: is the chemical functionality of the hydrogel important? *Chem. Sci.* **2020**, *11*, 232-240.
10. Pedersen, J. S.; Gerstenberg, M. C. The Structure of P85 Pluronic Block Copolymer Micelles Determined by Small-Angle Neutron Scattering. *Colloids Surf. A Physicochem. Eng. Asp.* **2003**, *213*, 175–187.
11. Pedersen, J. S.; Svaneborg, C.; Almdal, K.; Hamley, I. W.; Young, R. N. A Small-Angle Neutron and X-Ray Contrast Variation Scattering Study of the Structure of Block Copolymer Micelles: Corona Shape and Excluded Volume Interactions. *Macromolecules* **2003**, *36*, 416–433.
12. Pedersen, J. S.; Schurtenberger, P. Scattering Functions of Semiflexible Polymers with and without Excluded Volume Effects. *Macromolecules* **1996**, *29*, 7602–7612.
